# Disentangling river and swamp buffalo genetic diversity: initial insights from the 1000 Buffalo Genomes Project

**DOI:** 10.1093/gigascience/giae053

**Published:** 2024-09-09

**Authors:** Paulene S Pineda, Ester B Flores, Lilian P Villamor, Connie Joyce M Parac, Mehar S Khatkar, Hien To Thu, Timothy P L Smith, Benjamin D Rosen, Paolo Ajmone-Marsan, Licia Colli, John L Williams, Wai Yee Low, 1000 Buffalo Genomes Consortium, Lloyd Low, Mehar Khatkar, Tong Chen, Hanh Thi Hong Nguyen, Humberto Tonhati, Gregório Miguel Ferreira de Camargo, Stefano Biffani, Jianlin Han, Yi Zhang, Mei Liu, Yang Zhou, Divier Antonio Agudelo Gómez, P Kumarasamy, Jaswinder Singh Bhatti, Manishi Mukesh, Dwi Sendi Priyono, Akhmad Dakhlan, Mahdi Mokhber, John Williams, Ajmone Marsan Paolo, Licia Colli, Mayra Gómez Carpio, Roberta Cimmino, Ali Raza Awan, Paulene S Pineda, Lilian P Villamor, Ester B Flores, Connie Joyce Parac, Rangsun Parnpai, Siri Tuk, M İhsan Soysal, Emel Özkan Unal, Raziye Isik, Zhihua Jiang, Ðỗ Ðụ’c Lụ’c, Nguyen Hoang Thinh

**Affiliations:** The Davies Research Centre, School of Animal and Veterinary Sciences, University of Adelaide, Roseworthy, SA 5371, Australia; Philippine Carabao Center National Headquarters and Genepool, Science City of Muñoz, Nueva Ecija 3120, Philippines; Philippine Carabao Center National Headquarters and Genepool, Science City of Muñoz, Nueva Ecija 3120, Philippines; Philippine Carabao Center National Headquarters and Genepool, Science City of Muñoz, Nueva Ecija 3120, Philippines; Philippine Carabao Center National Headquarters and Genepool, Science City of Muñoz, Nueva Ecija 3120, Philippines; The Davies Research Centre, School of Animal and Veterinary Sciences, University of Adelaide, Roseworthy, SA 5371, Australia; Norwegian University of Life Sciences: NMBU, Universitetstunet 3, 1430 Ås, Norway; U.S. Meat Animal Research Center, USDA-ARS, Clay Center, NE 68933, USA; Animal Genomics and Improvement Laboratory, USDA-ARS, Beltsville, MD 20705, USA; Department of Animal Science, Food and Nutrition, Università Cattolica del Sacro Cuore, 29122 Piacenza, Italy; Department of Animal Science, Food and Nutrition, Università Cattolica del Sacro Cuore, 29122 Piacenza, Italy; The Davies Research Centre, School of Animal and Veterinary Sciences, University of Adelaide, Roseworthy, SA 5371, Australia; Department of Animal Science, Food and Nutrition, Università Cattolica del Sacro Cuore, 29122 Piacenza, Italy; The Davies Research Centre, School of Animal and Veterinary Sciences, University of Adelaide, Roseworthy, SA 5371, Australia

**Keywords:** buffalo genomics, whole-genome sequencing, carabao, SNP panel, structural variants

## Abstract

More people in the world depend on water buffalo for their livelihoods than on any other domesticated animals, but its genetics is still not extensively explored. The 1000 Buffalo Genomes Project (1000BGP) provides genetic resources for global buffalo population study and tools to breed more sustainable and productive buffaloes. Here we report the most contiguous swamp buffalo genome assembly (PCC_UOA_SB_1v2) with substantial resolution of telomeric and centromeric repeats, ∼4-fold more contiguous than the existing reference river buffalo assembly and exceeding a recently published male swamp buffalo genome. This assembly was used along with the current reference to align 140 water buffalo short-read sequences and produce a public genetic resource with an average of ∼41 million single nucleotide polymorphisms per swamp and river buffalo genome. Comparison of the swamp and river buffalo sequences showed ∼1.5% genetic differences, and estimated divergence time occurred 3.1 million years ago (95% CI, 2.6–4.9). The open science model employed in the 1000BGP provides a key genomic resource and tools for a species with global economic relevance.

## Introduction

Water buffalo (*Bubalus bubalis*) produce milk and meat to support rural economies. The global buffalo population is ∼230 million, mainly found in Asia. Water buffalo are adapted to hot climates, are tolerant of diseases that are a barrier to farming cattle, and can thrive on low-quality fodder [1, 2]. More people worldwide depend on water buffalo for their livelihoods than any other domesticated animals [3]. There are 2 types of water buffalo, river and swamp, each considered a subspecies with its distinct geographical distribution and biological traits, differing in body size, draft capacity, and milk and meat production [2, 4]. Despite lower productivity, swamp buffaloes are vital livestock in resource-limited regions of the world due to their resilience and adaptability [5]. Swamp buffaloes have 48 chromosomes, while river buffaloes have 50 chromosomes, with chromosome 1 in swamp buffalo being homologous to chromosomes 9 and 4 in river buffalo [6, 7]. The 2 water buffalo types can interbreed, resulting in fertile cross-bred offspring with 49 chromosomes [7]. The ancestral origin of the water buffalo is generally recognized to be from wild water buffalo *Bubalus arnee*, which originated in mainland Southeast Asia and later expanded to the Indian subcontinent, eventually diverging into a river buffalo [8, 9]. The swamp buffalo underwent 2 migration events, expanding southward to Indonesia and northward toward China, where it eventually moved southward into the Philippines. A series of postdomestication events followed independently for both water buffalo types, involving importation, isolation, and cross-breeding, which resulted in the formation of different water buffalo breeds and introgression of the river genetics to some swamp buffalo populations [9].

High-quality reference genomes provide the foundation for applying genomics in agriculture to conservation and selective breeding to improve animal health and productivity. Several buffalo genome sequences have been published, including 3 long read-based genome assemblies for river buffalo [10–12] and 2 for swamp buffalo [12, 13]. However, highly repetitive regions, such as the tandem arrays in the centromere and telomere, continue to be a challenge in assembling the genome as the high repetition makes it difficult to piece the sequences together, resulting in a fragmented genome assembly [14]. Variant detection can be impacted by the quality and representativeness of the reference genome, highlighting the significance of a high-quality reference genome that correctly represents the population for accurate variant calling [15]. Ideally, a reference genome should be highly contiguous, span the telomeres and centromeres, contain no gaps, and have high accuracy [15, 16].

Most genomics studies on water buffalo have focused on river types, as they are the most abundant and are mostly utilized in well-developed countries [4]. Several independent studies have produced whole-genome sequencing (WGS) short-read data for both river and swamp buffaloes [8, 12, 17]. A 90K single nucleotide polymorphism (SNP) genotyping tool also exists for water buffaloes [18]. The SNP panel can be used for genetic diversity studies in swamp-type buffalo [19], but the SNPs were designed based on the river type and may not be suitable for use in genomic analysis on swamp-type buffalo. Molecular genetic information has been accumulating in river buffalo, but there are limited resources for the swamp type. Collating the existing data and generating additional whole-genome sequences that equally represent both types globally will expand the understanding of water buffalo genetics and facilitate sustainable farming of water buffaloes.

The 1000 Buffalo Genome Project (1000BGP) [20] is an international consortium formed in 2022, comprising 38 researchers who have previous works on water buffalo from 15 countries. The project aims to create high-quality reference genomes for both subspecies of water buffalo and coordinate sampling and WGS data of global buffalo breeds. These data were made publicly accessible and will be used for subsequent and downstream analyses.

Here we report the assembly and annotation of a swamp buffalo genome (PCC_UOA_SB_1v2), having the best contiguity and repeat resolution of any water buffalo assembly to date. Using this swamp reference along with the previously generated river reference genome (UOA_WB_1) [11], we aligned 140 samples to call SNPs for the first run of the 1000BGP. We identified 13 million SNPs in both river and swamp breeds. The new assembly and catalog of SNPs provide a foundation of genetic resources for a species with global economic importance.

## Method

### Sample collection and DNA extraction

**Figure 1:**
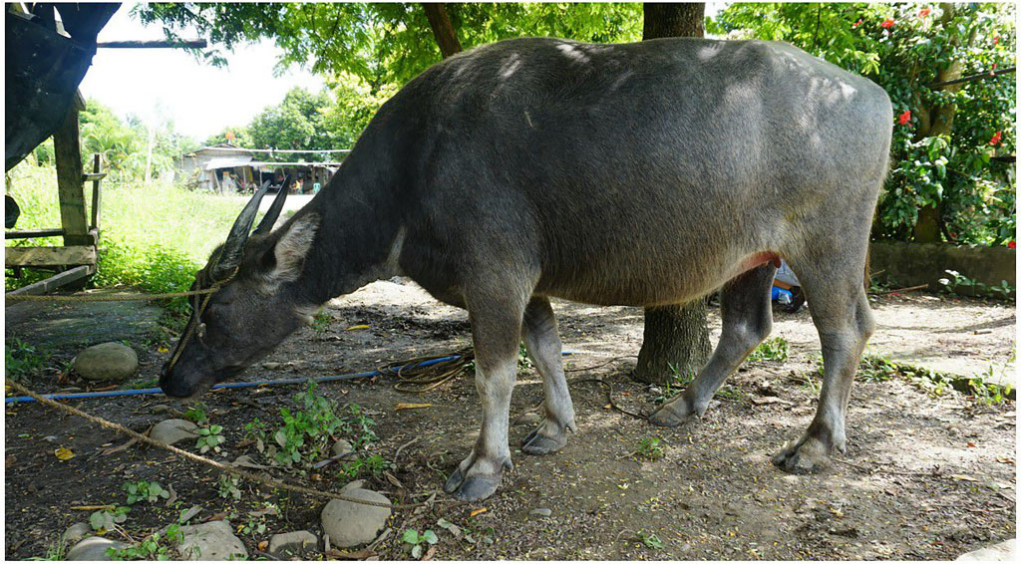
The female swamp buffalo from Kalinga Province, Philippines, was selected for whole-genome assembly.

All animal handling and procedures involved were approved by the Philippine Carabao Center Ethics Committee (Research Approval Code BG21001-ROG). A female carabao (NCBI:txid 3119969) from the Kalinga Province, Philippines, which represented 1 of the 3 major clusters of swamp buffalo in the country [21], was selected for genome sequencing (Fig. 1). The chosen animal was highly inbred as it came from a small herd of animals that was geographically isolated by mountains. Fresh blood was collected from the jugular vein into EDTA Vacutainer tubes and was kept cool on frozen gel packs for transportation to the laboratory and DNA extraction within 24 hours. Genomic DNA was isolated from the whole-blood sample using both Promega Wizard and Wizard® HMW DNA Extraction Kits following the manufacturer’s protocol and washing the DNA pellet up to 3 times in HMW lysis buffer to increase yield and purity.

### Library preparation and sequencing

The genomic DNA extracted with the Promega Wizard Kit was sequenced with Illumina NovaSeq to produce paired-end sequences. Low-quality bases and adapters from these short reads were trimmed using Trim Galore (v0.4.2) [22], and sequence quality was checked with FastQC (v0.11.4) [23]. To produce Hi-C short reads, a 200-µL blood sample was resuspended in 1% formaldehyde in a 15-mL conical tube and incubated for 20 minutes, with occasional mixing, and then 125 mM of glycine was added and incubated for a further 15 minutes with periodic mixing. The cross-linked blood was shipped to PhaseGenomics for Proximo Hi-C library preparation and sequencing. The restriction enzyme used was *DpnII* and a total of 400 million reads, 2 × 150-bp read pairs, were sequenced. Genomic DNA extracted with Promega Wizard and HMW Promega Wizard kits was sent to the US Department of Agriculture–Agricultural Research Service (USDA-ARS) for long-read sequencing using PacBio Sequel II. After DNA quality assessment, the sequencing library (>18 Kb) was prepared using the SMRTbell Express Template Prep Kit 2.0 following the USDA-ARS standard protocol for PacBio HiFi sequencing.

### Genome assembly, scaffolding, and polishing

The PacBio subread bam files were converted to HiFi reads using DeepConsensus (v0.3) [24]. Adapters were removed using the second release of HiFiAdapterFilt [25]. The raw coverage of PacBio HiFi reads was ∼29×, and after DeepConsensus, it was ∼34×. These reads were *de novo* assembled with HiFiasm (RRID:SCR_021069, v0.16.1-r375) [26] to produce a contig level assembly. The unphased contig assembly (primary) was used in the subsequent analysis because it was a more continuous assembly than HiFiasm phased assemblies. The PacBio HiFi long reads were then mapped to the contig assembly using minimap2 (v2.24-r1122) [27], and the alignments were used as input for purge_dups (v1.2.5) [28] to remove low-coverage (junks) and repeat contigs with size less than 1 Mb. Next, Hi-C short reads were processed following the Arima mapping pipeline [29] to map the reads to contigs. Then the contigs were scaffolded using YaHS (v1.2a.2) [30] without error correction to maintain the contigs assembled by HiFiasm [26]. The scaffolds were then aligned with the water buffalo genome UOA_WB_1 [11] and cattle genome ARS-UCD1.3 [31] using winnowmap (RRID:SCR_025349, v2.03) [32] to determine homologous chromosomes and the orientation of chromosome p and q arms. A Hi-C contact map was produced using juicer_tools (v1.8.9) [33] and visualized using Juicebox (RRID:SCR_021172, v1.11.08) [34] to check for misassemblies and to join scaffolds with strong Hi-C contact signals. These scaffolds were then aligned to homologous chromosomes of river buffalo and cattle with Gepard (v2.1) [35] to produce dot plots that allowed visual inspection of misassemblies. The identified chromosomes were then reoriented to a similar orientation as the ARS-UCD1.3 [31] homologous chromosomes using CombineFasta (v0.0.17) [36]. Next, gap filling was attempted with YAGCloser (v1.0.0) [37], but no gaps were filled. Further details and parameters for the different programs used can be found at https://github.com/plnspineda/ph_swamp_genome_assembly and Supplementary Table S1. The final assembly is available in the NCBI under the accession PCC_UOA_SB_1v2 (GCA_029407905.2).

### Genome size and assembly evaluation

Genome size and heterozygosity score were estimated using GenomeScope2 [38] from *k*-mer counts of Illumina short reads with ∼56× coverage using *k*-mers generated by meryl (v1.3) [39]. Base quality value (QV) of the assembly was assessed using Merqury (RRID:SCR_022964, v1.3) [39] using the *k*-mer counts. Genome assembly statistics were obtained using QUAST (RRID:SCR_001228, v4.5) [40]. The BUSCO completeness score was computed using BUSCO (RRID:SCR_015008, v5.4.4) [41], and the database used was mammalia_odb10. The completeness score based on *k*-mers was computed using Merqury.

### Mitochondrial genome assembly

The mitochondrial genome of the swamp buffalo was assembled with MitoHiFi (v2.2) [42]. A reference *B. bubalis* mitochondrial genome (Genbank ID OP921772.1) was used for comparison. The pairwise sequence identity of mitogenomes was determined using BLAST+ (v2.2.31) [43].

### Gaps and repeat analysis

Five water buffalo assemblies were used to compare gaps and sequence contiguity with the Philippine swamp genome (PCC_UOA_SB_1v2). Three assemblies were of river buffalo type (*B. bubalis*; NCBI:txid89462): Italian Mediterranean (UOA_WB_1) [11], Indian Murrah (NDDB_SH_1) [10], and Chinese Murrah (CUSA_RVB) [12]. Two assemblies were of the swamp type (*Bubalus kerabau*; NCBI:txid 3119969): a Chinese Fuzhong swamp buffalo assembly (CUSA_SWP) [12] and a male swamp buffalo labeled as Wang_2023 in our study [13]. These assemblies were either downloaded from the NCBI or the National Genomics Data Center (NGDC). Further information can be found in the Data Availability section. Repeat sequences in these genome assemblies were identified with RepeatMasker (RRID:SCR_012954, v4.1.4) [44] using a combined library of RepBaseRepeatMaskerEdition-20181026 and the default Dfam.h5, which used *B. bubalis* as the species reference. The repeats were filtered to keep matches that had >60% identity.

### Identification of telomeres and centromeres

Telomeric sequences in all 5 assemblies were identified with tidk (v0.2.31) [45] by searching for the TTAGGG telomeric repeats within the 20,000-bp window at both ends of the autosomes. Only telomeric repeat counts that were greater than 50 were kept (a series of TTAGGG was counted as 1). For centromeric repeats in autosomes, we used RepeatMasker (v4.1.4) [44] to find the “Satellite/centr” repeat family. Only repeats of this family with >60% identity were included for analysis. Repeats that were less than 1 Mbp from adjacent repeats were grouped. The groups with the most significant number of repeats on each chromosome were selected as candidate centromeric regions. To test whether this method can identify centromeric tandem array locations, we tested it on the human T2T genome (CHM13) and found that the approximate span of the centromeric region could be identified (Supplementary Table S2). The tandem repeats in the putative centromeric region of the swamp buffalo assembly were then identified using TRF (v.4.10.0) [46]. Finally, the candidate tandem repeats found by TRF were counted using HiCAT (1.0.0) [47].

### Genome annotation

The NCBI Eukaryotic Genome Annotation Pipeline was used to annotate genes, transcripts, proteins, and other genomic features [48]. The annotation process included 66,922 human RefSeq proteins, 14,224 cattle RefSeq proteins, and about ∼2.5 billion publicly available RNA sequencing reads. These were aligned to the swamp buffalo genome for gene predictions. We did not compare genome annotation with CUSA_SWP, CUSA_RVB and Wang_2023 because these were not annotated with the NCBI annotation pipeline.

### Estimation of divergence time

The divergence time between swamp-type and river-type buffaloes was estimated by constructing phylogenies based on single-copy ortholog (SCO) coding sequences (CDS) of 8 species using both IQ-TREE (RRID:SCR_017254) [49] and PAML (RRID:SCR_014932) [50]. The species included human (*Homo sapiens*), pig (*Sus scrofa*), goat (*Capra hircus*), sheep (*Ovis aries*), indicine cattle (*Bos indicus*), taurine cattle (*Bos taurus*), swamp buffalo (*B. bubalis kerabau*), and river buffalo (*B. bubalis*) (Supplementary Table S3). CDS of SCOs were identified from orthogroups using Orthofinder (RRID:SCR_017118) v2.4.0 [51] as implemented in the workflow found in https://gitlab.com/sandve-lab/salmonid_synteny. The SCOs were concatenated and used as input to create a phylogenetic tree with IQ-TREE (v2.2.2.3) [49] using 1,000 bootstrap replicates. Two different calculations, LSD2 [52] with IQ-TREE and Bayesian estimation methods with mcmctree, were used. The same concatenated SCOs were used to run PAML mcmctree (v4.10.6) [53] with independent rates to calculate divergence times. Two calibration times, human–cattle divergence of 61.5 to 131.5 million years ago (Mya) and cattle–sheep divergence of 18 to 28.55 Mya [54], were used as constraints for estimation of divergence times. To achieve convergence with an efficient sampling size (ESS) greater than 200, Bayesian Markov chain Monte Carlo inference was performed using a total of 4,020,000 iterations (comprising 20,000 burn-in iterations, 200 samples, and 20,000 sample frequency).

### Single nucleotide variant and structural variant identification by comparing assemblies

The 5 water buffalo assemblies (UOA_WB_1, NDDB_SH_1, CUSA_SWP, CUSA_RVB, and Wang_2023) were aligned with PCC_UOA_SB_1v2 using nucmer (v4.0.0) [55] to identify structural variants (SVs) and single nucleotide variants (SNVs). Gaps were removed in the assemblies to avoid N-to-N alignments. Large structural variants 50 to 10,000 bp in size were found using Assemblytics (v1.2.1) [56] from the nucmer alignment. SNVs were identified using the nucmer’s “show-snps –Clr” parameter to exclude SNVs within repeats. Unique and shared DNA variants among animals were visualized using upset plot data.

### SNP from the first run of 1000BGP

The first 1000BGP run was done with 80 swamp-type and 60 river-type buffaloes (Supplementary Table S4) using the GATK best practices for germline short variant discovery [57]. The chosen samples were based on submissions by members of the 1000BGP and contained almost all publicly available WGS data on 12 October 2024. The reference genomes used were swamp buffalo (PCC_UOA_SB_1v2) and river buffalo (UOA_WB_1). Briefly, the pipeline used Trim Galore (RRID:SCR_011847, v0.4.2) to remove low-quality bases and adapters, and sequence quality was checked with FastQC. The aligner bwa was used to align short WGS reads to PCC_UOA_SB_1v2 and UOA_WB_1. HaplotypeCaller was used to call variants per sample and chromosome in GVCF format. GenotypeGVCFs were used to genotype variants of all samples. A database of SNPs does not exist for water buffalo, so the following filters were applied: cluster_size=3, cluster_window_size=10, filter_expression=“(QD < 2.0) || (FS > 60.0) || (MQ < 40.0) || (MQRankSum < -12.5) || (ReadPosRankSum < -8.0).” The filter criteria for indels were cluster_size=3, cluster_window_size=10, filter_expression=“(QD < 2.0) || (FS > 60.0) || (MQ < 40.0) || (ReadPosRankSum < -8.0).” A dedicated snakemake workflow was created to streamline the first and all subsequent 1000BGP runs.

The counting of SNPs was done with BCFtools (RRID:SCR_005227, v1.17) [58], and the cumulative number of SNPs was computed for all buffalo samples using both swamp and river buffalo reference genomes. Principal component analysis (PCA) plots were performed using plink (v1.90) [59] after filtering the SNPs using the following parameters: –cow –nonfounders –allow-no-sex –autosome –geno 0.1 –mind 0.1 –maf 0.05, then pruning the SNPs based on linkage disequilibrium with the parameter –indep 50 5 2. Minor allele frequencies (MAFs) were also computed using plink with the same filtering criteria besides MAF, which is changed to 0.01. We identified ∼1.5 million SNPs that are highly polymorphic in swamp (MAF > 0.2) but were fixed in river buffaloes (MAF < 0.01) and ∼5 million SNPs in river that were fixed in swamp buffaloes. These SNP sites that have high polymorphism in one type and low or fixed in the other water buffalo type aligned with the swamp buffalo genome assembly were annotated using SnpEff (v.5.2a) [60]. The database for the swamp buffalo genome was built with the annotation file, coding, and protein sequences. When a gene had multiple transcripts, only the canonical transcript was chosen in annotating the impact of SNP. Genes with nonsynonymous mutations were recorded. A literature search was conducted by using the following search terms: “water buffalo GWAS” OR “water buffalo gene” OR “water buffalo association,” which covered more than 141 studies on water buffalo (Supplementary Table S5). These studies were scrutinized for genes that have an association with milk and reproductive traits. Genes found in the literature were then matched to the genes found with nonsynonymous mutations that have a high polymorphism in one type and low in the other type of buffalo. Comparison of SNPs between WGS and the Affymetrix Axiom Buffalo SNP array was done using the river buffalo (UOA_WB_1) [11] reference as both data types were based on the UOA_WB_1 SNP coordinates.

## Results

### *De novo* assembly

Sequencing of the female swamp buffalo generated ∼34× PacBio HiFi reads used for genome assembly, ∼473 million read pairs of Proximo HiC used for scaffolding, and ∼56× Illumina short reads of the same animal used to evaluate the genome assembly (Supplementary Table S6). The initial contig assembly with HiFiasm (v0.16.1-r375) produced 500 contigs spanning 2.95 Gb with a contig N50 of 85.47 Mb (Supplementary Table S7). After the removal of low-coverage contigs classified as junks, repeats less than 1 Mb, and contaminants identified as proteobacteria sequences, 137 contigs with an assembly size of 2.90 Gb and a contig N50 of 91.17 Mb were retained. Scaffolding produced 116 scaffolds with a final genome size of 2.90 Gb and scaffold N50 of 121.85 Mb. Around 6.5% of the total bases were classified as unplaced comprising 91 scaffolds. We identified a haploid set of 23 autosomes and an X chromosome that corresponds to the 24 chromosomes of the swamp buffalo (Supplementary Fig. S1).

A mitochondrial genome of 16,358 bp was also assembled, which had 99.79% identity with the Chinese swamp buffalo mitogenome (accession number: OP921772.1) and 97.67% identity with the Indian river buffalo mitogenome (accession number: NC_049568.1).

**Figure 2:**
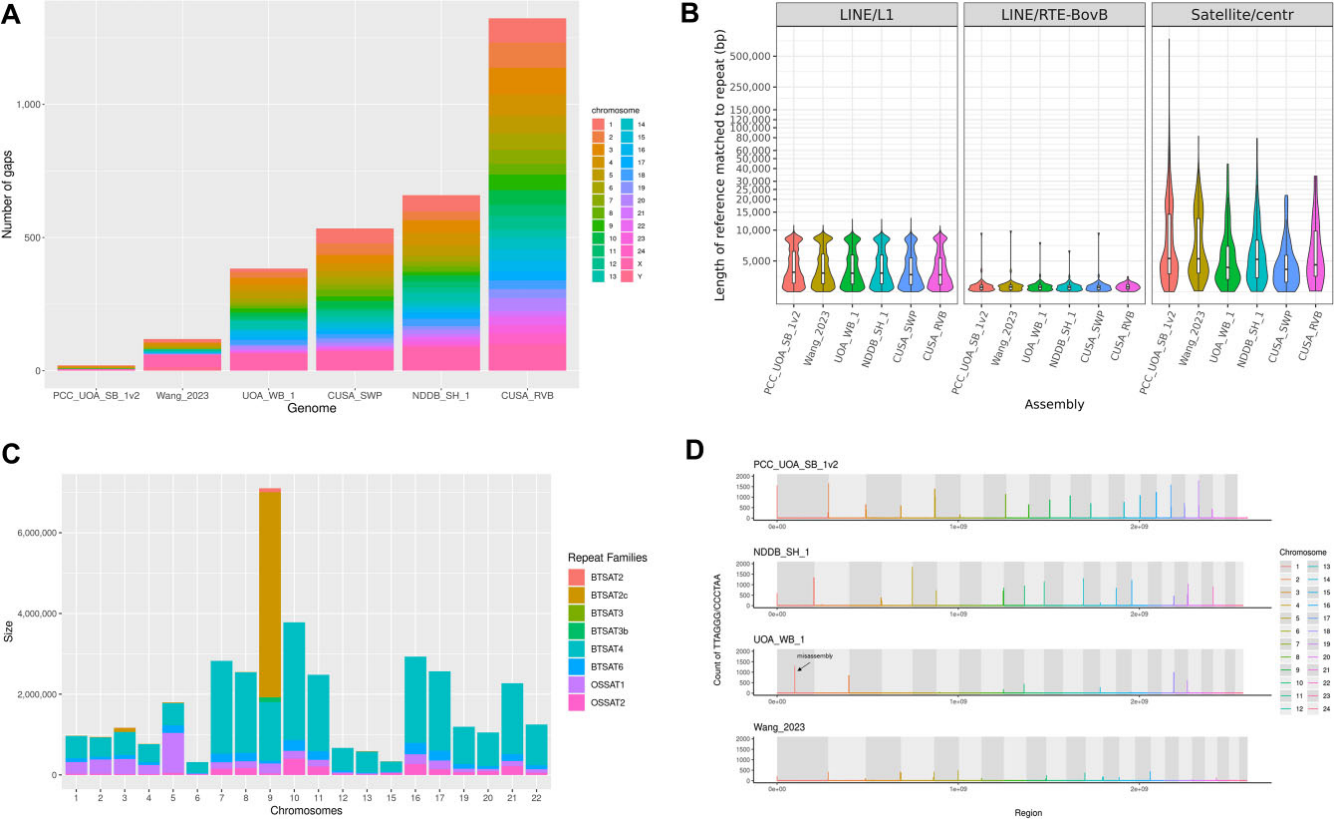
Comparison of gaps, major repeats, telomeric repeats, and centromeric repeats compared to other assemblies. (A) Barplot of the number of gaps per chromosome displaying the low number of gaps of the PCC_UOA_SB_1v2. (B) Violin plot for swamp and river buffalo genomes of repeat lengths >2 kb for LINE/L1, LINE/RTE-BovB, and satellite/centromeric repeats. The boxplot inside shows the quartile range and median. (C) Barplot of the centromeric satellite repeat families found in the tentative centromeric region of each chromosome. (D) Bedgraph for the telomeric signals of the 3 highly contiguous water buffalo assemblies. Telomeric count is equal to 1 unit of TTAGGG/CCCTGG. The red arrow represents the possible misassembly in chromosome 1 of UOA_WB_1.

The Philippine swamp buffalo genome (PCC_UOA_SB_1v2) has only 20 gaps (Fig. 2A, Table 1) spread across 8 autosomes and the X chromosome. Chromosome 4 and the X chromosome are the most fragmented chromosomes, but they only have 5 gaps each, whereas the next best, water buffalo X chromosome (UOA_WB_1), has 48 gaps. The contig N50 of the Philippine swamp buffalo was ∼4-fold higher than the river buffalo genome UOA_WB_1 (85.5 Mb vs. 22.4 Mb). Moreover, it also exceeded another male swamp buffalo genome, Wang_2023, by ∼13 Mb in terms of contig N50. Among the chromosomes, 15 contained a single contig or were gapless. Approximately 88% of the unplaced scaffolds consisted of repeat sequences, of which centromeric/satellite repeats were the majority, representing 131 Mb of the unplaced sequences.

**Table 1:** Assembly metrics of the Philippine swamp buffalo and 4 water buffalo genome assemblies are available in public databases. For NDDB_SH_1, gaps were reported as 17.44 Mb in size for its scaffold assembly. For Wang_2023, the assembly size and number of sequences were only an estimation since they were not reported. NA denotes not available

Assembly	Type	Assembly level	Assembly method	Assembly size (Gb)	N50 (Mb)	Number of sequences	Number of gaps	Reference
PCC_UOA_SB_1v2	Swamp	Contig	HiFiasm	2.95	85.5	500	0	This study
		Scaffold	YaHS	2.90	121.9	116	21	
		Chromosome	CombineFasta	2.70	121.9	24	20	
Wang_2023	Swamp	Contig	NextDenovo	2.68	72.2	173	0	Wang et al. [13]
		Scaffold	3D-DNA	2.68	120.03	33	140	
		Chromosome	Not specified	2.67	120.03	25	119	
UOA_WB_1	River	Contig	FALCON-Unzip	2.65	18.8	953	0	Low et al. [11]
		Scaffold	PacBio + Chicago + Hi-C	2.65	117.2	506	488	
		Chromosome	PBJelly, Arrow, Pilon	2.64	117.2	25	383	
NDDB_SH_1	River	Contig	FALCON	2.62	9.5	1,132	0	Ananthasayanam et al. [10]
		Scaffold	Scaff10x + BioNano	2.63	82.0	59	NA	
		Chromosome	RaGOO	2.62	117.5	25	659	
CUSA_SWP	Swamp	Contig	Wtdbg	2.61	8.8	2,003	0	Luo et al. [12]
		Scaffold	BioNano + HiC	2.63	117.3	1,534	536	
		Chromosome	Not specified	2.57	117.3	24	534	
CUSA_RVB	River	Contig	Wtdbg	2.63	3.1	3,482	0	Luo et al. [12]
		Scaffold	BioNano + HiC	2.65	116.1	2,304	1,323	
		Chromosome	Not specified	2.54	116.1	25	1,323	

### Repeats resolution

PacBio HiFi reads are highly accurate and long enough to span most repeats, and in fact, we observed that our PacBio HiFi-based swamp genome had resolved longer centromeric and satellite repeats than all the other long-read–based water buffalo assemblies; for instance, the total percentage of repeats was 0.84% in PCC_UOA_SB_1v2 vs. 0.09% in Wang_2023 (Fig. 2B, Supplementary Table S8). The Philippine swamp buffalo genome consisted of ∼51% repetitive sequences, which was slightly higher than other water buffalo assemblies that had ∼48% of total repeat sequences. The longest repeat family in the Philippine swamp buffalo genome belonged to long interspersed nuclear element (LINE), which was predominantly made up of L1 and retrotransposon of bovine B (RTE-BovB) that spanned a total of 694.52 Mb or ∼24% of the genome. Centromeres contained highly repetitive sequences and often caused gaps in the genome assemblies. Analysis of candidate centromeric regions with RepeatMasker identified a total of 8 repeat families (Fig. 2C). BTSAT4 was the most abundant repeat family, with a total length of 115.7 Mb and making up ∼4% of the genome. Two tandem repeats were detected with the tools TRF and HiCAT, and these repeats constituted the higher-order repeat (HOR) structure of the swamp buffalo centromeric region. The sizes of these tandem repeats were 1,404 bp and 673 bp with 4,160 and 3,582 copies, respectively (Supplementary Table S9). We denoted these tandem repeats as sat.1404 and sat.673. The sat.1404 was only found in acrocentric chromosomes, and sat.673 was seen in chromosomes 1 to 5 (submetacentric) and chromosome 9. In total, these satellite repeats in the centromeric region comprised approximately ∼6% of the genome.

Mammalian telomeres are tandem repeats of 5′-TTAGGG-3′ and are found at both ends of the chromosomes. The total telomeric repeat unit (TTAGGG)_n_ for PCC_UOA_SB_1v2 was 19,545 (∼117 Kbp), and the range of telomeric units across the chromosomes was between 637 (∼3.8 Kbp) and 2,369 (∼14 Kbp) (Supplementary Table S10; Fig. 2D). In comparison, the Chinese male swamp (Wang_2023) had a total of 5,240 telomeric repeats (∼31 Kbp). The best river buffalo reference (NDDH_SH_1), in terms of telomeric sequences, had 15,456 repeats (93 Kbp). On average, PCC_UOA_SB_1v2 had a higher count of telomeric repeats and number of telomeres at chromosomal ends than any other water buffalo assembly. Our swamp buffalo assembly had 3 sub-metacentric chromosomes (chr 1, chr 2, and chr 3) with telomeric repeats at both p- and q-arms; however, these chromosomes were not gapless. In both the Philippine swamp and Indian river buffalo genomes, telomeric repeats follow a distinct pattern: chromosomes with telomeric repeats at both ends were sub-metacentric, and none of the acrocentric chromosomes possessed telomeric repeats at the p-arms. While analyzing the location of telomeric repeats, we detected a misassembly in chromosome 1 of the UOA_WB_1 genome as it had a strong telomeric signal at position 97,361,828–97,370,520 (Fig. 2D). These telomeric repeats were ∼8 Kbp and found within a single contig spanning approximately 11 Kbp, which was scaffolded into chromosome 1.

### Genome assembly quality evaluation and annotation

The final genome size of 2.90 Gb was consistent with the estimated genome size from GenomeScope2.0 and was based on *k*-mers in short reads (Supplementary Fig. S2). This swamp buffalo genome size was ∼300 Mb larger than all other buffalo assemblies (Table 1). Assembly quality assessment of PCC_UOA_SB1v2 using Merqury showed base pair quality QV of 45.8 and completeness score of 95.9%. This assessment was done using short reads that were not used in the process of assembling the genome. The assembly also achieved a 95.7% BUSCO completeness score, suggesting a high-quality genome. The basepair quality (QV) of the Philippine swamp genome assembly outperformed the next most contiguous water buffalo assembly Wang_2023, which has a QV of 41.3.

The protein coding sequences, introns, exons, and transcript counts in the Philippine swamp buffalo genome were similar to the river buffalo assemblies. PCC_UOA_SB_1v2 contains a total of 21,871 protein-coding genes, 13,688 noncoding genes, and 4,726 nontranscribed pseudogenes.

Furthermore, the Philippine swamp buffalo genome contains 2,535 more genes compared to the NDDB_SH_1 water buffalo genome (Supplementary Table S11). Additional information on the annotation comparisons is given in Supplementary Note 1.

### Estimation of divergence time between swamp and river buffalo

**Figure 3:**
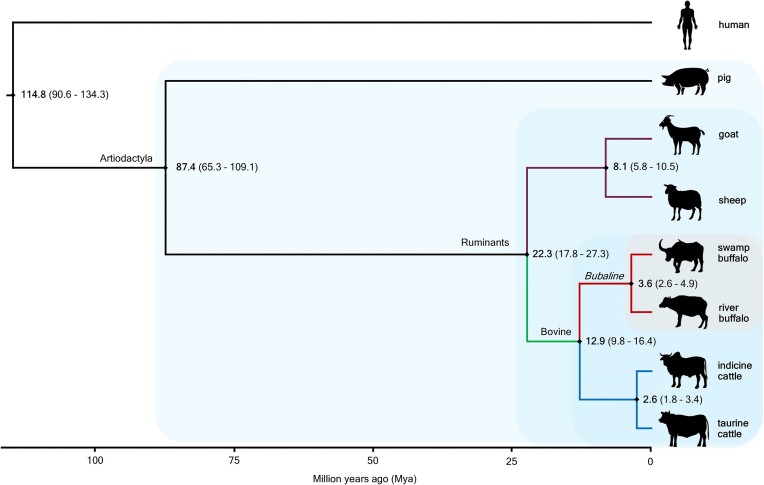
The phylogenetic tree of 8 species using single-copy ortholog genes indicating estimated time divergence and confidence interval from the present in Mya.

The divergence between swamp and river buffalo was estimated to be between 2.6 and 4.9 Mya, with a median value of 3.6 Mya, according to our analysis using the Bayesian method (mcmctree). This convergence was consistent with a separate estimate of between 2.2 and 4.3 Mya, with a median value of 3.1 Mya produced using LSD2 with IQTree (Supplementary Table S12). The Bayesian method was preferred over the simpler least squares method, so the median divergence time of 3.6 Mya from mcmctree was adopted for the rest of this article. The analysis used 11,976 SCOs identified by Orthofinder across 8 species. The phylogenetic tree from the concatenated SCOs of the 8 species showed ruminants grouping and the *Bovidae* family in the same cluster (Fig. 3).

### DNA variants from aligning genome assemblies

There were, on average, ∼6 million SNVs discovered from pairwise genome alignments between swamp buffalo assemblies (Table 2) and, on average, ∼7.4 million SNVs from pairwise comparisons of river buffalo assemblies. When a swamp assembly was aligned to a river assembly, ∼12 million SNVs were found, on average. There were, on average, 23,138 SVs that comprised ∼21 million bases found in pairwise comparisons of river buffalo assemblies. When a swamp assembly was aligned to a river assembly, 33,694 SVs that were made up of ∼30 million bases were found, on average. The river- and swamp-type buffalo divergence from autosomal SNP and SV is ∼1.5%.

**Table 2:** Number of SNPs and size of SVs (bp) from pairwise genome assembly alignment. Numbers above the diagonal are the total size of SVs, while below are the total number of SNPs

	Genome Assemblies	PCC_UOA_SB_1v2	Wang_2023	CUSA_SWP	UOA_WB_1	NDDB_SH_1	CUSA_RVB	
	**PCC_UOA_SB_1v2**	—	17,507,727	21,907,568	27,064,859	27,246,061	32,173,283	SV
	**Wang_2023**	6,315,498	—	22,452,637	27,918,711	28,155,313	32,953,777	
	**CUSA_SWP**	5,969,757	5,941,882	—	29,250,364	29,646,973	33,998,313	
	**UOA_WB_1**	12,375,163	12,376,399	11,930,093	—	16,771,137	23,217,345	
	**NDDB_SH_1**	12,437,983	12,470,447	11,984,056	7,999,500	—	23,562,063	
SNP	**CUSA_RVB**	12,093,156	12,082,957	11,896,391	7,758,580	7,771,824	—	

**Figure 4:**
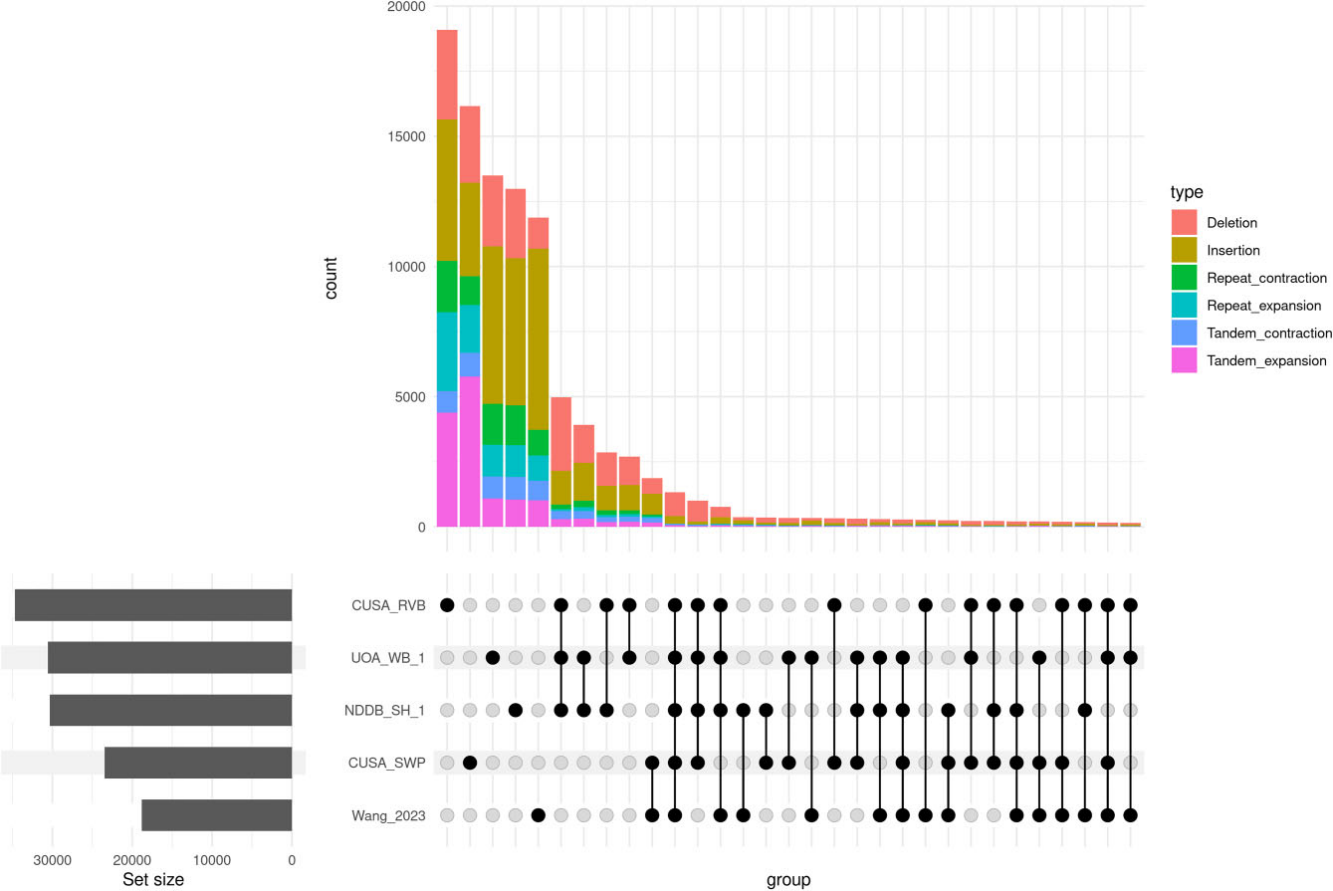
Upset plot of the intersection of different types of SVs identified in water buffalo assemblies when aligned to PCC_UOA_SV_1v2 (swamp type), which shows the number of shared and unique SVs between different water buffalo assemblies.

Most SVs detected in pairwise genome alignments were unique to each assembly with insertion, deletion, and tandem expansions being more common than other types of SVs (Fig. 4; Supplementary Fig. S3). On average, ∼14,000 SVs were unique to each assembly, which constituted ∼15 Mb or 0.6% of the genome. There were 5,289 SVs that were shared by the 3 swamp buffalo assemblies compared to the river buffalo reference (UOA_WB_1) (Supplementary Fig. S4). In contrast, 4,981 SVs were shared by the river buffalo assemblies compared to the swamp buffalo reference (PCC_UOA_SB_1v2).

### Discovery of SNPs in buffaloes

The first phase of the 1000BGP analyzed WGS data of 140 animals and identified a total of 41,632,997 and 41,071,165 SNPs using PCC_UOA_SB_1v2 and UOA_WB_1 as reference genomes, respectively (Table 3), with a Ti/Tv (transitions vs. transversions) ratio of 2.12. An average of 25 million SNPs were identified for each buffalo type when selecting only the autosomes, biallelic loci, sample call rates >90%, and SNP call rates >90%. Of the SNPs identified using the PCC_UOA_SB_1v2, ∼14 million SNPs were river buffalo specific, whereas ∼10 million SNPs were swamp buffalo specific. When UOA_WB_1 was used as the reference, ∼11 million SNPs were specific to river-type buffaloes, and ∼12 million SNPs were specific to swamp type. Regardless of the reference genome choice, ∼13 million SNPs with a MAF > 1% were shared between the 2 types and many of these can be considered ancestral variations.

**Table 3:** Summary of SNP counts by reference genome and concordant SNPs with the 90K SNP buffalo genotyping array. Only the SNPs aligned with UOA_WB_1 were used to determine concordant SNPs in the 90K SNP buffalo genotyping array. Description for the SNPs rows was as follows: All = all SNPs found without filtering; Autosomes only = all SNPs found in the autosomes without another filtering; Swamp after QC = SNPs identified in swamp buffalo animals after quality filtering; River after QC = SNPs identified in river buffalo animals after quality filtering; Swamp specific = SNPs identified only in the swamp (not in river) buffalo animals after quality filtering; River specific = SNPs identified only in the river (not in swamp) buffalo animals after quality filtering; River and swamp shared = SNPs identified in both river and swamp buffalo animals after quality filtering

SNPs	PCC_UOA_SB_1v2	UOA_WB_1	90K SNP buffalo array
All	41,632,997	41,071,165	90,000
Autosomes only	40,905,045	40,340,557	72,434
Swamp after QC	22,847,574	24,914,052	39,278
River after QC	26,525,477	24,485,667	65,890
Swamp specific	10,161,461	11,756,460	278
River specific	13,839,364	11,328,075	26,890
River and swamp shared	12,686,113	13,157,592	39,000

Approximately 1.5 million SNPs were found to be polymorphic (MAF > 0.2) in swamp but were fixed in river buffaloes. Most of these variants were in the intergenic (∼48%) and intronic (∼36%) regions. Moreover, ∼99% were SNPs classified as modifiers by snpEff, which were predicted to have a minor impact as they are often found in noncoding regions. However, 0.24%, 0.43, and 0.01% have moderate, low, and high putative impact, respectively. The impacts were based on position in coding regions and type of amino acid changes. Among SNPs with predicted impact, 4,863 were nonsynonymous mutations that affected 3,338 genes, which were polymorphic in swamp buffaloes but fixed in river buffaloes. Of the 3,338 genes, 57 are associated with milk and reproductive traits (Supplementary Table S13).

There were ∼5 million SNPs in river buffaloes that were fixed in swamp buffaloes. Of these SNPs, 36,890 were predicted to have an impact and 12,796 were nonsynonymous mutations that affected 6,657 genes. Of these 6,657 genes, 130 were associated with milk production traits and reproductive traits (Supplementary Table S14).

**Figure 5:**
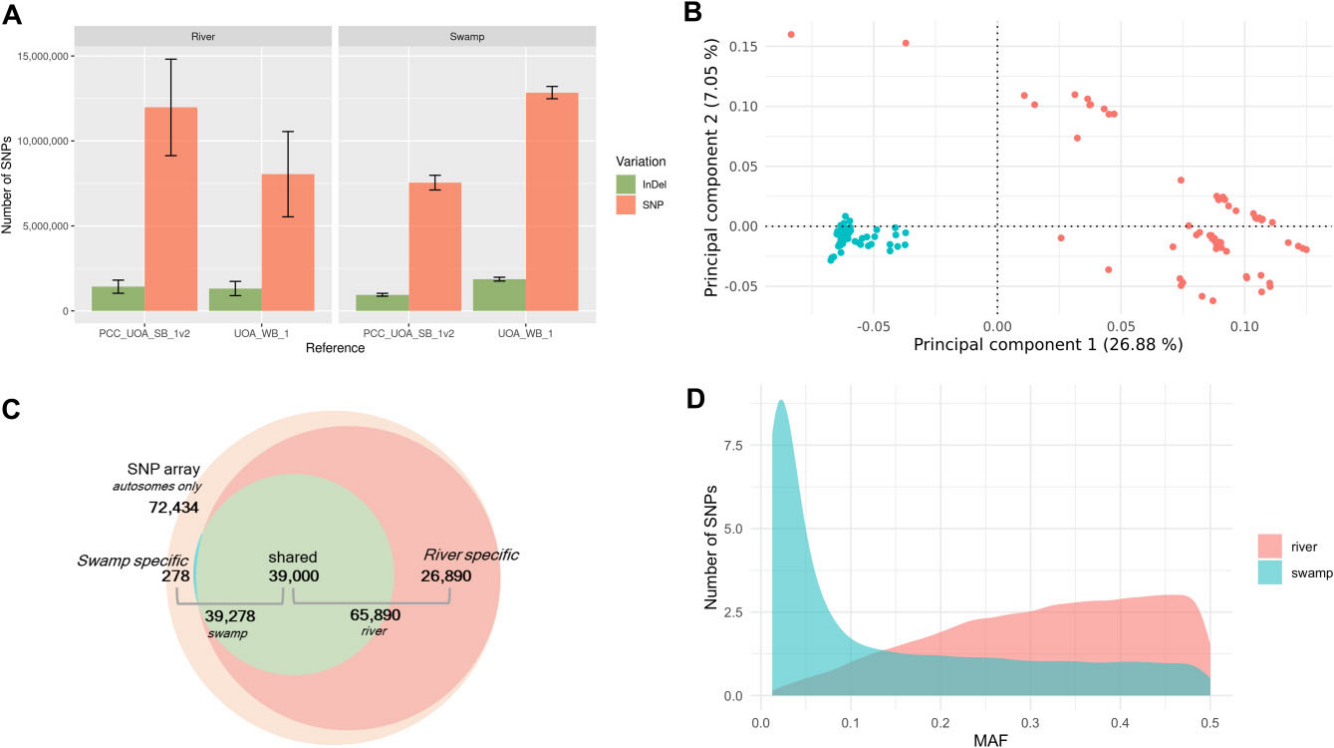
The first phase of the 1000BGP. (A) Bar graph of the average number of SNPs with standard deviation in swamp and river buffalo aligned with PCC_UOA_SB_1v2 (swamp type) and UOA_WB_1 (river type). (B) PCA plot using the swamp buffalo reference genome (PCC_UOA_SB_1v2) showing clear clustering of the swamp and river buffaloes. (C) Venn diagram of the number of autosomal SNPs in the 90K SNP buffalo genotyping array that are shared and specific for each water buffalo type. The large light peach circle shows the number of SNPs found in the 90K array; among these SNPs, the dark peach color shows river-specific SNPs, blue shows swamp-specific SNPs, and green shows SNPs shared for both types. (D) Histogram plot of the SNPs in the 90K SNP buffalo genotyping array, including both specific and shared SNPs per MAF value binned at 0.01.

The average number of SNPs found in the short reads from the 140 samples were ∼8 million SNPs and ∼1 million indels when using the reference genome from the same water buffalo type (Fig. 5A; Supplementary Tables S15–S16). The cumulative count of SNPs was lower when the sample and reference genome were from the same water buffalo type; for example, fewer SNPs were found for the Binhu breed, a swamp-type buffalo, when mapped to the swamp reference, PCC_UOA_SB_1v2, than to a river buffalo reference (Supplementary Figs. S6–S7). Some SNPs were fixed within a subspecies, which would not be scored if the respective subspecies reference was used, and were likely to be new mutations that occurred after the divergence of the buffalo subspecies from the common ancestor. A distinct genetic differentiation between the 2 water buffalo subspecies was observed. The PCA plot explained 34% of variation coming from ∼3 million SNPs regardless of reference genome choice (Fig. 5B; Supplementary Fig. S8). Swamp buffaloes display lower average heterozygosity per sample compared to river buffaloes (1.75 vs. 1.88 heterozygous sites per kb).

Comparing the SNPs aligned with UOA_WB_1 to the 90K SNP buffalo genotyping array, about 26,890 SNPs were polymorphic only in river type while 278 SNPs were polymorphic only in swamp type (Fig. 5C). Nevertheless, 39,000 SNPs were polymorphic in both river and swamp types. However, 55% of the SNPs in the swamp type have MAF < 0.1 (Fig. 5D; Supplementary Table S17). Only ∼12,450 SNPs had MAF > 0.2 for swamp-type buffaloes in the 90K SNP array, which accounted for only ∼17% of all the SNPs in the panel, whereas 74% of the SNPs were highly polymorphic in river-type buffaloes.

## Discussion

The accurate PacBio HiFi long-read sequences have facilitated the assembly of highly contiguous genomes, including the human genome [26, 61]. Here, we presented a PacBio HiFi-based swamp buffalo genome assembly, which is more contiguous than other assemblies of the same species [10–13]. This Philippine swamp buffalo genome assembly has a higher contig N50 (85.5 Mb vs. 72.2 Mb), with fewer gaps (21 vs. 140) and a higher Merqury QV score (45.8 vs. 41.3), than the next best water buffalo genome [13]. It also exceeds other water buffalo genome assemblies [10–13] in the resolution of many types of repeats, including telomeric and centromeric satellite sequences. The better resolution of repeats is likely to be the reason why our swamp buffalo assembly is larger than the other water buffalo assemblies. Genome assemblies that used HiFi reads, such as human [61], Hanwoo cattle [62], and sheep [63], also have larger genome sizes than previously published genome sizes for the same species. The satellite DNA sequences that we identified in the sub-metacentric (sat.673) and acrocentric (sat.1404) chromosomes are the same satellite repeats identified by 2 studies of water buffaloes [64, 65]. These repeats have ∼80% similarity to the bovine satellite I and II sequences and are both localized in the centromeric regions of both water buffalo types [7]. We designated the second satellite repeat as sat.1404, instead of 1378 described in Pathak et al. [65], since the average length of the tandem repeats is 1,404 bp. The sat.673 repeats were found in all the water buffalo chromosomes [64, 65], but we only found this satellite repeat in the sub-metacentric chromosomes and chromosome 9. There were no complete centromeres in any of our chromosomes, which was because the HiFi reads alone could not completely span the repeats in centromeres. The quality of genome assemblies will improve as the accuracy of sequence reads, such as PacBio HiFi [66], and length of reads, such as Oxford Nanopore duplex [67], increase.

Here we also report the first phase analysis from 1000BGP on 140 water buffaloes, of which 60 are river buffaloes and 80 are swamp buffaloes, with DNA variants identified using the buffalo genomes from the 2 buffalo types as reference (UOA_WB_1 and PCC_UOA_SB_1v2). There were ∼41 million SNPs discovered, and the average number of heterozygous sites per individual was 1.81 per kilobase, which is higher than humans [68] and cattle [69]. The numbers of river- or swamp-specific SNPs were influenced by the choice of reference genomes, which could be due to read mapping bias in the reference genome [70].

The river buffaloes are more valued for their milk and have undergone a more organized breeding program compared to the swamp buffaloes. The river buffaloes have ∼5 million SNPs that were fixed in the swamp buffaloes. One notable gene with a nonsynonymous SNP (g.2754274C>T) is *DGAT1*, which is a well-known gene associated with milk production traits [71]. The SNP corresponds to *DGAT1* g.11,785 T > C in another study that reported the TC and TT genotypes associated with higher fat and protein percentages in milk, respectively [72]. Note the coordinates of the SNPs differ because they were discovered with different reference genomes. In swamp buffaloes, the SNP has a low frequency of the T allele (0.6%), whereas in river buffaloes, the T allele frequency is higher at 21%. This difference in allele frequency could be the result of different selective pressures on milk fats. The g.2754274C>T SNP leads to a change in the protein sequence from alanine (Ala) to valine (Val) at position 494 (p.Ala494Val). This amino acid change has a moderate impact on the protein sequence. Several other genes with nonsynonymous mutations (e.g., *SASS6, VPS13B, ADGRA1, DNAH11, UBQLN4, PLEKHG7, ADAMTS9, DOCK7, ZNF292*, and *AKAP6*) were candidate genes for milk yield [73–80].

We also found 22 and 9 genes with nonsynonymous SNPs known to be linked with reproductive traits in the river buffaloes and swamp buffaloes, respectively. Among these genes, the *KISS1* and *KISS1R* genes were associated with fertility traits in a gene expression study in ovarian follicular tissue in buffalo [81]. The *KISS1* encodes for the kisspeptin and *KISS1R* is the kisspeptin receptor, and they play a role in hormonal regulation that influences fertility traits such as gonadotropin releasing hormone and luteinizing hormone in ruminants [82]. The nonsynonymous mutations have moderate impacts on *KISS1* (g.55887161G>A) and *KISS1R* (g.212308648C>A), which change the protein sequence from alanine to valine at position 133 (p.Ala133Val) and alanine to glutamic acid at position 36 (p.Ala36Glu), respectively. Among the polymorphic genes in river buffaloes that are fixed in swamp buffaloes, some genes such as *CAST* and CAPN have a strong association with meat tenderness in cattle [83], which could be the result of selective pressure for draft work in swamp buffaloes.

PCA analysis with ∼3 million autosomal SNPs that were polymorphic in both buffalo types clearly showed distinct genetic differentiation of river- and swamp-type buffaloes. The PCA plot (Fig. 5B) shows a tight clustering of the swamp buffaloes and a loose clustering of the river buffaloes, which is similar to other water buffalo population studies using a 90K buffalo SNP panel [9, 84] and a high-density cattle SNP array [85]. Admixture analysis of river and swamp buffaloes by Sun et al. [8] showed the distinctiveness of the Mediterranean breed and that introgression of the river type is evident in certain swamp buffaloes. This may be due in part to the interbreeding of the 2 types to improve milk production.

We estimated the divergence of the river and swamp buffalo to be between 2.6 and 4.9 Mya, which is consistent with the 2.2 to 5.4 Mya divergence reported by Luo et al. [12]. Although natural mating between river- and swamp-type buffaloes is possible, it requires weeks to months for a riverine bull to socialize and successfully breed with swamp buffaloes. Furthermore, the 2 types of water buffalo do not live in the same natural environments and have only been present in the same geographical location recently due to the importation of the river-type buffaloes to Southeast Asia and Southern American countries to upgrade traits such as milk and meat production [86]. It is possible to generate fertile hybrids of river and swamp buffaloes [7], and as such, these 2 types of buffalo are still best defined as subspecies.

The genetic diversity captured in our dataset is sufficient for us to investigate the representativeness of SNP markers on the current 90K SNP array panel at genotyping river- and swamp-type buffaloes. The current Axiom 90K Buffalo SNP array (Thermofisher) was created using data from river-type buffaloes, and the SNP showed high levels of heterozygosity in river buffaloes [18, 77, 87]. In the present study, 55% of SNPs in swamp buffalo samples detected in the 90K SNP array have MAF < 0.1. The SNP array was designed based on the polymorphism of 4 river buffalo breeds [18], so the limited performance of SNPs in swamp buffalo samples is unsurprising. We found 13 million SNPs from the first 1000BGP run that are polymorphic in both river and swamp buffaloes with MAF > 0.01. This SNP dataset presents an opportunity to design a genotyping panel suitable for both buffalo types. The SNP lists of this work are publicly available at the consortium’s website found at https://1000buffalogenomes.github.io/datamgmt. The first and subsequent runs of 1000BGP SNP lists will be useful to those working on selection signatures, domestication signals [88], breed identification, screening for recessive lethal mutations [89], and many other uses.

In conclusion, we presented a high-quality swamp buffalo genome sequence that enabled analyses of genomic features missing from previous buffalo genome assemblies. There were distinct genetic differences between the river and swamp buffalos. We showed that reference genome choice affected the identification of genetic variants probably because it affected the alignment of short-read sequences. The first run of the 1000BGP identified a large number of SNPs, including variants that were common between both types of buffalo, for the design of a new genotyping SNP panel. In the future, the project aims to increase the data available on global water buffalo samples to increase information on water buffalo genetics. Further goals of the 1000BGP consortium are to create a buffalo pangenome graph using available long-read assemblies of different breeds and to generate phased telomere-to-telomere assemblies of a river × swamp buffalo hybrid to enable complete characterization of centromeres and other difficult to assemble genomic regions.

## Supplementary Material

giae053_GIGA-D-24-00094_Original_Submission

giae053_GIGA-D-24-00094_Revision_1

giae053_GIGA-D-24-00094_Revision_2

giae053_Response_to_Reviewer_Comments_Original_Submission

giae053_Response_to_Reviewer_Comments_Revision_1

giae053_Reviewer_1_Report_Original_Submission James Prendergast -- 4/23/2024 Reviewed

giae053_Reviewer_2_Report_Original_Submission Paul Stothard -- 4/25/2024 Reviewed

giae053_Supplemental_Files

## Data Availability

The PacBio HiFi reads, Hi-C reads, and Illumina paired-end reads are available in the SRA under BioProject PRJNA901059. The BioSample of the animal is SAMN31703457. The genome accession number for PCC_UOA_SB_1v2 is GCA_029407905.2. The assemblies UOA_WB_1 (GCA_003121395.1) and NDDB_SH_1 (GCA_019923935.1) were downloaded from NCBI. The assemblies CUSA_SWP (GWHAAJZ00000000) and CUSA_RVB (GWHAAKA00000000) were downloaded in NGDC. The assembly Wang_2023 was downloaded from Figshare [90] as stated in Wang et al. [13]. Annotation files are available through NCBI with RefSeq GCF_029407905.1. All additional supporting data, including the identified variants and scripts produced in this study, are available in the *GigaScience* database, GigaDB [91].

## Additional Files

**Supplementary Note 1**. Further details on genome annotation.

**Supplementary Fig. S1**. Circos plot of swamp buffalo chromosome mapped to river buffalo. Chromosome 1 of the swamp buffalo showed clear homology to chromosomes 4 and 9 of the river buffalo.

**Supplementary Fig. S2**. Genomescope2 profile showing *k*-mer spectra of the short reads and inferring total genome length (len), percentage of the genome that is nonrepetitive or unique (uniq), percentage of homozygosity (aa) and heterozygosity (ab), mean *k*-mer coverage for heterozygous bases (kcov), error rate of the reads (err), average rate of duplicate reads (dup), *k*-mer size used (k), and number of set of chromosomes (p).

**Supplementary Fig. S3**. An upset plot of the number of different types of structural variants (SVs) identified when aligned to UOA_WB_1 (river type), which shows shared and unique SVs between various water buffalo assemblies.

**Supplementary Fig. S4**. Bar graph of the number of different types of structural variants (SVs) shared between swamp buffalo assemblies (PCC_UOA_SB_1v2, Wang_2023, and CUSA_SWP) when aligned to river buffalo assembly (UOA_WB_1).

**Supplementary Fig. S5**. Bar graph of the number of the number of different types of structural variants (SVs) shared between river buffalo assemblies (UOA_WB_1, NDDB_SH_1, CUSA_RVB) when aligned to swamp buffalo assembly (PCC_UOA_SB_1v2).

**Supplementary Fig. S6**. A line plot showing the cumulative number of SNPs of swamp-type buffalo samples per breed when aligned to swamp or river buffalo reference genomes.

**Supplementary Fig. S7**. A line plot showing the cumulative number of SNPs of river-type buffalo samples per breed when aligned to swamp or river buffalo reference genomes.

**Supplementary Fig. S8**. Principal component analysis (PCA) plot using the river buffalo reference genome (UOA_WB_1) shows clear clustering of the swamp and river buffaloes.

**Supplementary Table S1**. Software used in the study for *de novo* assembly, assessment, comparison, and analysis.

**Supplementary Table S2**. Estimated satellite arrays of the human T2T genome assembly using RepeatMasker. The T2T-CHM13v1 column is from Table 5 of Nurk et al. (2022) showing coordinates of alpha and human satellite arrays in v1.0 assembly.

**Supplementary Table S3**. Data accession number and links for species used in the estimation of divergence.

**Supplementary Table S4**. Whole-genome short-read sequence information on the samples for the 1000 Buffalo Genomes Project.

**Supplementary Table S5**. List of article searches for water buffalo genomes.

**Supplementary Table S6**. Sequencing reads.

**Supplementary Table S7**. Assembly statistics.

**Supplementary Table S8**. Percentage of repeat sequences and length of repeat families in the water buffalo assemblies. Repeat alignment lengths less than 2.5 Kbp were filtered out. Numbers are in base pair (bp).

**Supplementary Table S9**. Sizes in base pairs (bp) of the satellite repeat types within the estimated centromeric region per chromosome of the Philippine swamp genome. The repeat types sat.1404 and sat.673 are a subset of the repeat families identified by RepeatMasker.

**Supplementary Table S10**. Number of telomeric repeats across 5 water buffalo assemblies within a 20-Kbp window of each end of the chromosomes. Telomere counts less than 50 were filtered out. One telomeric repeat is equivalent to TTAGGG1.

**Supplementary Table S11**. Comparisons of various assembly features of the water buffalo genome assemblies available in NCBI. The Male swamp buffalo, Fuzhong swamp buffalo, and Murrah river buffalo are annotated differently. NA denotes not available.

**Supplementary Table S12**. Estimated divergence time and confidence interval of the 8 species.

**Supplementary Table S13**. List of genes polymorphic in swamp buffaloes but fixed in river buffaloes with corresponding traits from research articles on water buffaloes.

**Supplementary Table S14**. List of genes polymorphic in river buffaloes but fixed in swamp buffaloes with corresponding traits from research articles on water buffaloes.

**Supplementary Table S15**. Number of SNPs, indels, and cumulative SNPs of swamp buffaloes per sample using swamp and river reference genomes.

**Supplementary Table S16**. Number of SNPs, indels, and cumulative SNPs of river buffaloes per sample using swamp and river reference genomes.

**Supplementary Table S17**. Number of SNPs per MAF range with intervals of 0.1 using swamp and river reference genomes, as well as SNP concordance with the 90K SNP buffalo genotyping array.

## Abbreviations

1000BGP: 1000 Buffalo Genome Project; BLAST: Basic Local Alignment Search Tool; BUSCO: Benchmarking Universal Single-Copy Orthologs; CDS: coding sequences; ESS: efficient sampling size; Gb: gigabases; MAF: minor allele frequency; Mb: megabases; Mya: million years ago; NCBI: The National Center for Biotechnology Information; NGDC: National Genomics Data Center; PCA: principal component analysis; QV: quality value; SCO: single-copy ortholog; SNP: single nucleotide polymorphism; SNV: single nucleotide variant; SV: structural variant; USDA-ARS: US Department of Agriculture–Agricultural Research Service.
