# Supplementary material for: Disentangling river and swamp buffalo genetic diversity: initial insights from the 1000 Buffalo Genomes Project
Source: Gigascience. 2024 Sep 9;13:giae053. doi: 10.1093/gigascience/giae053 (PMC11382405; doi:10.1093/gigascience/giae053)
Supplement: giae053_GIGA-D-24-00094_Revision_2 [file giae053_giga-d-24-00094_revision_2.pdf]

GigaScience

Disentangling river- and swamp-buffalo genetic diversity: Initial Insights from the 1000 Buffalo Genomes Project

--Manuscript Draft--

**Manuscript Number:**	GIGA-D-24-00094R2
**Full Title:**	Disentangling river- and swamp-buffalo genetic diversity: Initial Insights from the 1000 Buffalo Genomes Project
**Article Type:**	Research
**Funding Information:**	Philippine Carabao Center (BG21001-ROG) — Dr Ester B. Flores<br>USDA-ARS — Dr Timothy P. L. Smith<br>Science Education Institute, Department of Science and Technology, Republic of the Philippines — Ms. Paulene Pineda<br>University of Adelaide — Ms. Paulene Pineda
**Abstract:**	Background<br>More people in the world depend on water buffalo for their livelihoods than on any other domesticated animals, but its genetics is still not extensively explored. The 1000 Buffalo Genomes Project (1000BGP) provides genetic resources for global buffalo population study and tools to breed more sustainable and productive buffaloes.<br><br>Results<br>Here we report the most contiguous swamp buffalo genome assembly (PCC_UOA_SB_1v2) with substantial resolution of telomeric and centromeric repeats, ~4-fold more contiguous than the existing reference river buffalo assembly and exceeding a recently published male swamp buffalo genome. This assembly was used along with the current reference to align 140 water buffalo short-read sequences and produce a public genetic resource with an average of ~41 million SNPs per swamp and river buffalo genome. Comparison of the swamp and river buffalo sequences showed ~1.5% genetic differences, and estimated divergence time occurred 3.1 million years ago (Mya) (95% CI: 2.6 to 4.9).<br><br>Conclusion<br>The open science model employed in this project (the “1000 buffalo genomes project; 1000BGP) provides a key genomic resource and tools for a species with global economic relevance.
**Corresponding Author:**	Paulene Pineda, Ph.D.<br>The University of Adelaide<br>Roseworthy, South Australia AUSTRALIA
**Corresponding Author Secondary Information:**	
**Corresponding Author's Institution:**	The University of Adelaide
**Corresponding Author's Secondary Institution:**	
**First Author:**	Paulene Pineda, Ph.D.
**First Author Secondary Information:**	
**Order of Authors:**	Paulene Pineda, Ph.D.
	Ester B. Flores
	Lilian P. Villamor
	Connie Joyce Parac
	Mehar S. Khatkar

	Hien To Thu
	Timothy P. L. Smith
	Benjamin D. Rosen
	Paolo Ajmone-Marsan
	Licia Colli
	John L. Williams
	Wai Yee Low
**Order of Authors Secondary Information:**	
**Response to Reviewers:**	Thank you very much for accepting our manuscript for publication. We also appreciate your pointers and have made the following revisions:<br><br>1. Added citations at REFERENCES section at number 91 and short description of the data at Line 677.<br>2. Moved the URL citations to the REFERENCE section, specifically at entries 20, 22, 36, 37, 45 and 48.<br>3. Included the DOI at entry 90 in the REFERENCES section.<br>4. Added the ORCID number of the authors at line 3-8.<br>5. Provided the NCBI taxon number for swamp and river buffalo at line 102, 169 and 171.<br>6. Restructured the abstract as suggested, now at line 22-38.<br><br>Thank you once again for your guidance and assistance!<br><br>Best regards,<br>Paulene S. Pineda
**Additional Information:**	
**Question**	**Response**
Are you submitting this manuscript to a special series or article collection?	No
**Experimental design and statistics**<br><br>Full details of the experimental design and statistical methods used should be given in the Methods section, as detailed in our Minimum Standards Reporting Checklist. Information essential to interpreting the data presented should be made available in the figure legends.<br><br>Have you included all the information requested in your manuscript?	Yes
**Resources**<br><br>A description of all resources used, including antibodies, cell lines, animals and software tools, with enough information to allow them to be uniquely	Yes

identified, should be included in the Methods section. Authors are strongly encouraged to cite Research Resource Identifiers (RRIDs) for antibodies, model organisms and tools, where possible.<br><br>Have you included the information requested as detailed in our Minimum Standards Reporting Checklist?	
**Availability of data and materials**<br><br>All datasets and code on which the conclusions of the paper rely must be either included in your submission or deposited in publicly available repositories (where available and ethically appropriate), referencing such data using a unique identifier in the references and in the "Availability of Data and Materials" section of your manuscript.<br><br>Have you have met the above requirement as detailed in our Minimum Standards Reporting Checklist?	Yes

Powered by Editorial Manager® and ProduXion Manager® from Aries Systems Corporation

Manuscript
Click here to access/download;Manuscript;wgs_swampbuf_for_production.d

Disentangling river- and swamp-buffalo genetic diversity: Initial Insights from the 1000 Buffalo Genomes Project

Paulene S. Pineda[1,2] [0000-0002-7147-3302], Ester B. Flores[2] [0000-0001-9057-1664], Lilian P. Villamor[2] [0000-0002-8013-6703], Connie Joyce M. Parac[2] [0000-0003-2609-6699], Mehar S. Khatkar[1] [0000-0002-4554-1404], Thu, Hien To[3] [0000-0003-0662-9734], Timothy P.L. Smith[4] [0000-0003-1611-6828], Benjamin D. Rosen[5] [0000-0001-9395-8346], Paolo Ajmone-Marsan[6] [0000-0003-3165-4579], Licia Colli[6] [0000-0002-7221-2905], John L. Williams[1,6] [0000-0001-5188-7957], Wai Yee Low*[1] [0000-0002-0749-765X] & 1000 Buffalo Genomes Consortium

[1]The Davies Research Centre, School of Animal and Veterinary Sciences, University of Adelaide, Roseworthy, SA 5371, Australia

[2]Philippine Carabao Center National Headquarters and Genepool, Science City of Muñoz, Nueva Ecija, Philippines 3120

[3]Norwegian University of Life Sciences: NMBU, Universitetstunet 3, 1430 Ås, Norway

[4]U.S. Meat Animal Research Center, USDA-ARS, Clay Center, Nebraska, USA

[5]Animal Genomics and Improvement Laboratory, USDA-ARS, Beltsville, MD, 20705, USA

[6]Department of Animal Science, Food and Nutrition, Università Cattolica del Sacro Cuore, 29122 Piacenza, Italy

Corresponding Author: Wai Yee Low (wai.low@adelaide.edu.au)

Background

More people in the world depend on water buffalo for their livelihoods than on any other domesticated animals, but its genetics is still not extensively explored. The 1000 Buffalo Genomes Project (1000BGP) provides genetic resources for global buffalo population study and tools to breed more sustainable and productive buffaloes.

Results

Here we report the most contiguous swamp buffalo genome assembly (PCC_UOA_SB_1v2) with substantial resolution of telomeric and centromeric repeats, ~4-fold more contiguous than the

existing reference river buffalo assembly and exceeding a recently published male swamp buffalo genome. This assembly was used along with the current reference to align 140 water buffalo short-read sequences and produce a public genetic resource with an average of ~41 million SNPs per swamp and river buffalo genome. Comparison of the swamp and river buffalo sequences showed ~1.5% genetic differences, and estimated divergence time occurred 3.1 million years ago (Mya) (95% CI: 2.6 to 4.9).

Conclusion

The open science model employed in this project (the “1000 buffalo genomes project”; 1000BGP) provides a key genomic resource and tools for a species with global economic relevance.

Keywords: buffalo genomics, whole-genome sequencing, carabao, SNP panel, structural variants

INTRODUCTION

Water buffalo (*Bubalus bubalis*) produce milk and meat to support rural economies. The global buffalo population is ~230 million, mainly found in Asia. Water buffalo are adapted to hot climates, are tolerant of diseases that are a barrier to farming cattle and can thrive on low-quality fodder [1, 2]. More people worldwide depend on water buffalo for their livelihoods than any other domesticated animals [3]. There are two types of water buffalo, river and swamp, each considered a subspecies with its distinct geographical distribution and biological traits, differing in body size, draft capacity, and milk and meat production [2, 4]. Despite lower productivity, swamp buffaloes are vital livestock in resource-limited regions of the world due to their resilience and adaptability [5]. Swamp buffaloes have 48 chromosomes, while river buffaloes have 50 chromosomes, with chromosome 1 in swamp buffalo being homologous to chromosomes 9 and 4 in river buffalo [6, 7]. The two water buffalo types can interbreed, resulting in fertile cross-bred offspring with 49 chromosomes [7]. The ancestral origin of the water buffalo is generally recognized to be from wild water buffalo *Bubalus arnee*, which originated in mainland Southeast Asia and later expanded to the Indian subcontinent, eventually diverging into a river buffalo [8, 9]. The swamp buffalo underwent two migration events, expanding southward to Indonesia and northward toward China

where it eventually moved southwards into the Philippines. A series of post-domestication events followed independently for both water buffalo types, involving importation, isolation and cross-breeding, that resulted in the formation of different water buffalo breeds and introgression of the river genetics to some swamp buffalo populations [9].

High-quality reference genomes provide the foundation for applying genomics in agriculture to conservation and selective breeding to improve animal health and productivity. Several buffalo genome sequences have been published, including three long read-based genome assemblies for river [10-12] and two for swamp buffalo [12, 13]. However, highly repetitive regions, such as the tandem arrays in the centromere and telomere, continue to be a challenge in assembling the genome as the high repetition makes it difficult to piece the sequences together resulting in a fragmented genome assembly [14]. Variant detection can be impacted by the quality and representativeness of the reference genome, highlighting the significance of a high-quality reference genome that correctly represents the population for accurate variant calling [15]. Ideally, a reference genome should be highly contiguous, span the telomeres and centromeres, contain no gaps, and have high accuracy [15, 16].

Most genomics studies on water buffalo have focused on river types, as they are the most abundant and are mostly utilized in well-developed countries [4]. Several independent studies have produced whole-genome short-read sequences for both river and swamp buffaloes [8, 12, 17]. A 90K SNP genotyping tool also exists for water buffaloes [18]. The SNP panel can be used for genetic diversity studies in swamp-type buffalo [19], but the SNPs were designed based on the river type and may not be suitable for use in genomic analysis on swamp-type buffalo. Molecular genetic information has been accumulating in river buffalo, but there are limited resources for the swamp-type. Collating the existing data and generating additional whole-genome sequences that equally represent both types globally will expand the understanding of water buffalo genetics and facilitate sustainable farming of water buffaloes.

The 1000 Buffalo Genome Project (1000BGP) [20] is an international consortium formed in 2022, comprising 38 researchers who have previous works on water buffalo from 15 countries. The project aims to create high-quality reference genomes for both subspecies of water buffalo and coordinate sampling and WGS sequencing of global buffalo breeds. These data were made publicly accessible and will be used for subsequent and downstream analyses.

Here we report the assembly and annotation of a swamp buffalo genome (PCC_UOA_SB_1v2), having the best contiguity and repeat resolution of any water buffalo assembly to date. Using this swamp reference along with the previously generated river reference genome (UOA_WB_1) [11], we aligned 140 samples to call SNPs for the first run of the 1000 buffalo genomes project. We identified 13 million SNPs in both river and swamp breeds. The new assembly and catalog of SNPs provide foundation genetic resources for a species with global economic importance.

METHODOLOGY

Sample collection and DNA extraction

All animal handling and procedures involved were approved by the Philippine Carabao Center Ethics Committee (Research Approval Code BG21001-ROG). A female carabao (NCBI:txid 3119969) from the Kalinga Province, Philippines, which represented one of the three major clusters of swamp buffalo in the country [21], was selected for genome sequencing (Figure 1). The chosen animal was highly inbred as it came from a small herd of animals that was geographically isolated by mountains. Fresh blood was collected from the jugular vein into EDTA vacutainer tubes and was kept cool on frozen gel packs for transportation to the laboratory and DNA extraction within 24 hours. Genomic DNA was isolated from the whole-blood sample using both Promega Wizard and Wizard® HMW DNA Extraction Kits following the manufacturer’s protocol and washing the DNA pellet up to 3x in HMW lysis buffer to increase yield and purity.

Library preparation and sequencing

The genomic DNA extracted with the Promega Wizard Kit was sequenced with Illumina NovaSeq to produce paired-end sequences. Low-quality bases and adapters from these short reads were trimmed using Trim Galore (v0.4.2) [22] and sequence quality was checked with FastQC (v0.11.4) [23]. To produce Hi-C short reads, a 200uL blood sample was resuspended in 1% formaldehyde in a 15mL conical tube and incubated for 20 minutes, with occasional mixing, then 125mM of glycine was added and incubated for a further 15 minutes with periodic mixing. The cross-linked blood was shipped to PhaseGenomics for Proximo HiC library preparation and sequencing. The restriction enzyme used was *DpnII* and a total of 400 million reads 2 x 150 bp read pairs were sequenced. Genomic DNA extracted with Promega Wizard and HMW Promega Wizard kits was sent to the USDA-ARS for long-read sequencing using PacBio Sequel II. After DNA quality assessment, the sequencing library (>18 Kb) was prepared using the SMRTbell Express Template Prep Kit 2.0 following the USDA-ARS standard protocol for PacBio HiFi sequencing.

Genome assembly, scaffolding, and polishing

The PacBio subread bam files were converted to HiFi reads using DeepConsensus (v0.3) [24]. Adapters were removed using the second release of HiFiAdapterFilt [25]. The raw coverage of PacBio HiFi reads was ~29x, and after DeepConsensus it was ~34x. These reads were *de novo* assembled with HiFiasm (RRID:SCR_021069, v0.16.1-r375) [26] to produce a contig level assembly. The unphased contig assembly (primary) was used in the subsequent analysis because it was a more continuous assembly than HiFiasm phased assemblies. The PacBio HiFi long reads were then mapped to the contig assembly using minimap2 (v2.24-r1122) [27] and the alignments were used as input for purge_dups (v1.2.5) [28] to remove low-coverage (junks) and repeat contigs with size less than 1 Mb. Next, Hi-C short-reads were processed following the Arima mapping pipeline [29] to map the reads to contigs. Then the contigs were scaffolded using YaHS (v1.2a.2) [30] without error correction to maintain the contigs assembled by HiFiasm [26]. The scaffolds were then aligned with the water buffalo genome UOA_WB_1 [11] and cattle genome ARS-UCD1.3 [31] using winnowmap (RRID:SCR_025349, v2.03) [32] to determine homologous chromosomes and the orientation of chromosome p and q arms. A Hi-C contact map was produced using juicer_tools (v1.8.9) [33] and visualized using Juicebox (RRID:SCR_021172, v1.11.08) [34] to check for mis-

assemblies and to join scaffolds with strong Hi-C contact signals. These scaffolds were then aligned to homologous chromosomes of river buffalo and cattle with Gepard (v2.1) [35] to produce dot plots that allowed visual inspection of mis-assemblies. The identified chromosomes were then reoriented to a similar orientation as the ARS-UCD1.3 [31] homologous chromosomes using CombineFasta (v0.0.17) [36]. Next, gap filling was attempted with YAGCloser (v1.0.0) [37] but no gaps were filled. Further details and parameters for the different programs used can be found at https://github.com/plnspineda/ph_swamp_genome_assembly and Table 1_Supplementary Material. The final assembly is available in the National Center for Biotechnology Information (NCBI) under the accession PCC_UOA_SB_1v2 (GCA_029407905.2).

Genome size and assembly evaluation

Genome size and heterozygosity score were estimated using GenomeScope2 [38] from k-mer counts of Illumina short-reads with ~56x coverage using k-mers generated by meryl (v1.3) [39]. Base quality value (QV) of the assembly was assessed using Merqury (RRID:SCR_022964, v1.3) [39] using the k-mer counts. Genome assembly statistics were obtained using QUAST (RRID:SCR_001228, v4.5) [40]. The BUSCO completeness score was computed using BUSCO (RRID:SCR_015008, v5.4.4) [41] and the database used was mammalia_odb10. The completeness score based on k-mers was computed using Merqury.

Mitochondrial genome assembly

The mitochondrial genome of the swamp buffalo was assembled with MitoHiFi (v2.2) [42]. A reference *Bubalus bubalis* mitochondrial genome (Genbank ID OP921772.1) was used for comparison. The pairwise sequence identity of mitogenomes was determined using BLAST+ (v2.2.31) [43].

Gaps and repeat analysis

Five water buffalo assemblies were used to compare gaps and sequence contiguity with the Philippine swamp genome (PCC_UOA_SB_1v2). Three assemblies were of river buffalo type (*Bubalus bubalis*; NCBI:txid89462): Italian Mediterranean (UOA_WB_1) [11], Indian Murrah

(NDDB_SH_1) [10] and Chinese Murrah (CUSA_RVB) [12]. Two assemblies were of the swamp-type (*Bubalus kerabau*; NCBI:txid 3119969): a Chinese Fuzhong swamp buffalo assembly (CUSA_SWP) [12] and a male swamp buffalo labelled as Wang_2023 in our study [13]. These assemblies were either downloaded from the NCBI, or the National Genomics Data Center (NGDC). Further information can be found in the Data Availability section. Repeat sequences in these genome assemblies were identified with RepeatMasker (RRID:SCR_012954, v4.1.4) [44] using a combined library of RepBaseRepeatMaskerEdition-20181026 and the default Dfam.h5, which used *Bubalus bubalis* as the species reference. The repeats were filtered to keep matches that had >60% identity.

Identification of telomeres and centromeres

Telomeric sequences in all five assemblies were identified with tidk (v0.2.31) [45] by searching for the TTAGGG telomeric repeats within the 20,000 bp window at both ends of the autosomes. Only telomeric repeat counts that were greater than 50 were kept (a series of TTAGGG was counted as one). For centromeric repeats in autosomes, we used RepeatMasker (v4.1.4) [44] to find the "Satellite/centr" repeat family. Only repeats of this family with >60% identity were included for analysis. Repeats that were less than 1Mbp from adjacent repeats were grouped. The groups with the most significant number of repeats on each chromosome were selected as candidate centromeric regions. To test whether this method can identify centromeric tandem array locations, we tested it on the human T2T genome (CHM13) and found that the approximate span of the centromeric region could be identified (Table 2_Supplementary Material). The tandem repeats in the putative centromeric region of the swamp buffalo assembly were then identified using TRF (v.4.10.0) [46]. Finally, the candidate tandem repeats found by TRF were counted using HiCAT (1.0.0) [47].

Genome annotation

The NCBI Eukaryotic Genome Annotation Pipeline was used to annotate genes, transcripts, proteins and other genomic features [48]. The annotation process included 66,922 human RefSeq proteins, 14,224 cattle RefSeq proteins and about ~2.5 billion publicly available RNA-seq reads.

These were aligned to the swamp buffalo genome for gene predictions. We did not compare genome annotation with CUSA_SWP, CUSA_RVB and Wang_2023 because these were not annotated with the NCBI annotation pipeline.

Estimation of divergence time

The divergence time between swamp-type and river-type buffaloes was estimated by constructing phylogenies based on single-copy orthologous (SCOs) coding sequences (CDS) of eight species using both IQ-TREE (RRID:SCR_017254) [49] and PAML (RRID:SCR_014932) [50]. The species included human (*Homo sapiens*), pig (*Sus scrofa*), goat (*Capra hircus*), sheep (*Ovis aries*), indicine cattle (*Bos indicus*), taurine cattle (*Bos taurus*), swamp buffalo (*Bubalus bubalis kerabau*), and river buffalo (*Bubalus bubalis*) (Table 3_Supplementary Material). CDS of SCOs were identified from orthogroups using Orthofinder (RRID:SCR_017118) v2.4.0 [51] as implemented in the workflow found in https://gitlab.com/sandve-lab/salmonid_synteny. The SCOs were concatenated and used as input to create a phylogenetic tree with IQ-TREE (v2.2.2.3) [49] using 1000 bootstrap replicates. Two different calculations, LSD2 [52] with IQ-TREE and Bayesian estimation methods with mcmctree were used. The same concatenated SCOs were used to run PAML mcmctree (v4.10.6) [53] with independent rates to calculate divergence times. Two calibration times, human-cattle divergence of 61.5 to 131.5 Mya and cattle-sheep divergence of 18 to 28.55 Mya [54], were used as constraints for estimation of divergence times. To achieve convergence with an efficient sampling size (ESS) greater than 200, Bayesian MCMC inference was performed using a total of 4,020,000 iterations (comprising 20,000 burn-in iterations, 200 samples, and 20,000 sample frequency).

SNV and SV identification by comparing assemblies

The five water buffalo assemblies (UOA_WB_1, NDDB_SH_1, CUSA_SWP, CUSA_RVB and Wang_2023) were aligned with PCC_UOA_SB_1v2 using nucmer (v4.0.0) [55] to identify structural variants (SV) and single nucleotide variants (SNVs). Gaps were removed in the assemblies to avoid N-to-N alignments. Large structural variants 50 bp to 10,000 bp in size were found using Assemblytics (v1.2.1) [56] from the nucmer alignment. SNVs were identified using the nucmer's

'show-snps –Clr' parameter to exclude SNVs within repeats. Unique and shared DNA variants among animals were visualized using upset plot data.

SNP from the first run of 1000BGP

The first 1000BGP run was done with 80 swamp-type and 60 river-type buffaloes (Table 4_Supplementary Material) using the GATK best practices for germline short variant discovery [57]. The chosen samples were based on submissions by members of the 1000 BGP and contained almost all publicly available WGS data on October 12, 2024. The reference genomes used were swamp buffalo (PCC_UOA_SB_1v2) and river buffalo (UOA_WB_1). Briefly, the pipeline used Trim Galore (RRID:SCR_011847, v0.4.2) to remove low-quality bases and adapters, and sequence quality was checked with FastQC. The aligner bwa was used to align short WGS reads to PCC_UOA_SB_1v2 and UOA_WB_1. HaplotypeCaller was used to call variants per sample and chromosome in GVCF format. GenotypeGVCFs was used to genotype variants of all samples. A database of SNPs does not exist for water buffalo, so the following filters were applied: cluster_size=3, cluster_window_size=10, filter_expression="(QD < 2.0) || (FS > 60.0) || (MQ < 40.0) || (MQRankSum < -12.5) || (ReadPosRankSum < -8.0)". The filter criteria for indels were cluster_size=3, cluster_window_size=10, filter_expression="(QD < 2.0) || (FS > 60.0) || (MQ < 40.0) || (ReadPosRankSum < -8.0)". A dedicated snakemake workflow was created to streamline the first and all subsequent 1000BGP runs.

The counting of SNPs was done with BCFtools (RRID:SCR_005227, v1.17) [58] and the cumulative number of SNPs was computed for all buffalo samples using both swamp and river buffalo reference genomes. Principal Component Analysis (PCA) plots were performed using plink (v1.90) [59] after filtering the SNPs using the following parameters: --cow --nonfounders --allow-no-sex --autosome --geno 0.1 --mind 0.1 --maf 0.05, then pruning the SNPs based on linkage disequilibrium with the following parameter --indep 50 5 2. Minor allele frequencies were also computed using plink with the same filtering criteria besides MAF which is changed to 0.01. We identified ~1.5 million SNPs that are highly polymorphic in swamp (MAF > 0.2) but were fixed in river buffaloes (MAF < 0.01) and ~5 million SNPs in river that were fixed in swamp buffaloes.

These SNP sites that have high polymorphism in one type and low or fixed in the other water buffalo type aligned with the swamp buffalo genome assembly were annotated using SnpEff (v.5.2a) [60]. The database for the swamp buffalo genome was built with the annotation file, coding, and protein sequences. When a gene had multiple transcripts, only the canonical transcript was chosen in annotating the impact of SNP. Genes with non-synonymous mutations were recorded. A literature search was conducted by using the search terms: “water buffalo GWAS” OR “water buffalo gene” OR “water buffalo association”, which covered more than 141 studies on water buffalo (Table 5_Supplementary Material). These studies were scrutinized for genes that have an association with milk and reproductive traits. Genes found in the literature were then matched to the genes found with non-synonymous mutations that have a high polymorphism in one type and low in the other type of buffalo. Comparison of SNPs between WGS and the Affymetrix Axiom Buffalo SNP array was done using the river buffalo (UOA_WB_1) [11] reference as both data types were based on the UOA_WB_1 SNP coordinates.

RESULTS

De novo **assembly**

Sequencing of the female swamp buffalo generated ~34x PacBio HiFi reads used for genome assembly, ~473 million read pairs of Proximo HiC used for scaffolding, and ~56x Illumina short reads of the same animal used to evaluate the genome assembly (Table 6_Supplementary Material). The initial contig assembly with HiFiasm (v0.16.1-r375) produced 500 contigs spanning 2.95 gigabases (Gb) with a contig N50 of 85.47 megabases (Mb) (Table 7_Supplementary Material). After the removal of low-coverage contigs classified as junks, repeats less than 1 Mb and contaminants identified as proteobacteria sequences, 137 contigs with assembly size of 2.90 Gb and a contig N50 of 91.17 Mb were retained. Scaffolding produced 116 scaffolds with a final genome size of 2.90 Gb and scaffold N50 of 121.85 Mb. About 6.5% of the total bases were classified as unplaced comprising 91 scaffolds. We identified a haploid set of 23 autosomes and an X chromosome that corresponds to the 24 chromosomes of the swamp buffalo (Figure 1_Supplementary Material).

A mitochondrial genome of 16,358 bp was also assembled which had 99.79% identity with the Chinese swamp buffalo mitogenome (Accession number: OP921772.1) and 97.67% identity with the Indian river buffalo mitogenome (Accession number: NC_049568.1).

The Philippine swamp buffalo genome (PCC_UOA_SB_1v2) has only 20 gaps (Figure 2A, Table 1) spread across eight autosomes and X chromosome. The chromosome 4 and X chromosome are the most fragmented chromosomes, but they only have five gaps each whereas the next best water buffalo X chromosome (UOA_WB_1) had 48 gaps. The contig N50 of the Philippine swamp buffalo was ~4-fold higher than the river buffalo genome UOA_WB_1 (85.5 Mb vs 22.4 Mb). Moreover, it also exceeded another male swamp buffalo genome Wang_2023 by ~13 Mb in terms of contig N50. Among the chromosomes, 15 of them contained single contig or were gapless. Approximately 88% of the unplaced scaffolds consisted of repeat sequences, of which centromeric/satellite repeats were the majority, representing 131 Mb of the unplaced sequences.

Repeats resolution

PacBio HiFi reads are highly accurate and long enough to span most repeats, and in fact we observed that our PacBio HiFi-based swamp genome had resolved longer centromeric and satellite repeats than all the other long read based water buffalo assemblies, for instance, the total percentage of repeats were 0.84% in PCC_UOA_SB_1v2 vs 0.09% in Wang_2023 (Figure 2B, Table 8_Supplementary Material). The Philippine swamp buffalo genome consisted of ~51% repetitive sequences, which was slightly higher than other water buffalo assemblies that had ~48% of total repeat sequences. The longest repeat family in the Philippine swamp buffalo genome belonged to Long Interspersed Nuclear Element (LINE), which was predominantly made up of L1 and Retrotransposon of Bovine B (RTE-BovB) that spanned a total of 694.52 Mb or ~24% of the genome. Centromeres contained highly repetitive sequences and often caused gaps in the genome assemblies. Analysis of candidate centromeric regions with RepeatMasker identified a total of eight repeat families (Figure 2C). BTSAT4 was the most abundant repeat family, with a total length of 115.7 Mb and making up ~4% of the genome. Two tandem repeats were detected with the tools TRF and HiCAT and these repeats constituted the higher-order repeat (HOR) structure of

the swamp buffalo centromeric region. The sizes of these tandem repeats were 1,404 bp and 673 bp with 4,160 and 3,582 copies, respectively (Table 9_Supplementary Material). We denoted these tandem repeats as sat.1404 and sat.673. The sat.1404 was only found in acrocentric chromosomes and sat.673 was seen in chromosomes 1 to 5 (submetacentric) and chromosome 9. In total, these satellite repeats in the centromeric region comprised approximately ~6% of the genome.

Mammalian telomeres are tandem repeats of 5'-TTAGGG-3' and are found at both ends of the chromosomes. The total telomeric repeat unit $(TTAGGG)_n$ for PCC_UOA_SB_1v2 were 19,545 (~117 Kbp), and the range of telomeric units across the chromosomes was between 637 (~3.8 Kbp) and 2369 (~14 Kbp) (Table 10_Supplementary Material; Figure 2D). In comparison, the Chinese male swamp (Wang_2023) had a total of 5,240 telomeric repeats (~31 Kbp). The best river buffalo reference (NDDH_SH_1), in terms of telomeric sequences, had 15,456 repeats (93 Kbp). On average, PCC_UOA_SB_1v2 had higher count of telomeric repeats and number of telomeres at chromosomal ends than any other water buffalo assembly. Our swamp buffalo assembly had three sub-metacentric chromosomes (chr 1, chr 2 and chr 3) with telomeric repeats at both p- and q-arms; however, these chromosomes were not gapless. In both the Philippine swamp and Indian river buffalo genomes, telomeric repeats follow a distinct pattern: chromosomes with telomeric repeats at both ends were sub-metacentric and none of the acrocentric chromosomes possess telomeric repeats at the p-arms. While analyzing the location of telomeric repeats, we detected a mis-assembly in chromosome 1 of the UOA_WB_1 genome as it had a strong telomeric signal at position 97,361,828 - 97,370,520 (Figure 2D). These telomeric repeats were ~8 Kbp and found within a single contig spanning approximately 11 Kbp, which was scaffolded into chromosome 1.

Genome assembly quality evaluation and annotation

The final genome size of 2.90 Gb was consistent with the estimated genome size from GenomeScope2.0 and was based on k-mers in short reads (Figure 2_Supplementary Material). This swamp buffalo genome size was ~300 Mb larger than all other buffalo assemblies (Table 1).

Assembly quality assessment of PCC_UOA_SB1v2 using Merqury showed base pair quality QV of 45.8 and completeness score of 95.9%. This assessment was done using short reads that were not used in the process of assembling the genome. The assembly also achieved 95.7% BUSCO completeness score suggesting a high-quality genome. The base-pair quality (QV) of the Philippine swamp genome assembly outperformed the next most contiguous water buffalo assembly Wang_2023, which has QV of 41.3.

The protein coding sequences, introns, exons, and transcript counts in the Philippine swamp buffalo genome were similar to the river buffalo assemblies. PCC_UOA_SB_1v2 contains a total of 21,871 protein-coding genes, 13,688 non-coding genes, and 4,726 non-transcribed pseudogenes. Furthermore, the Philippine swamp buffalo genome contains 2,535 more genes compared to the NDDB_SH_1 water buffalo genome (Table 11_Supplementary Material). Additional information on the annotation comparisons is given in Supplementary Note 1.

Estimation of divergence time between swamp and river buffalo

The divergence between swamp and river buffalo was estimated to be between 2.6 to 4.9 million years ago (Mya) with a median value of 3.6 Mya according to our analysis using the Bayesian method (mcmctree). This convergence was consistent with a separate estimate of between 2.2 to 4.3 Mya with a median value of 3.1 Mya produced using LSD2 with IQTreee (Table 12_Supplementary Material). The Bayesian method was preferred over the simpler least square method, so the median divergence time of 3.6 Mya from mcmctree was adopted for the rest of this paper. The analysis used 11,976 single-copy orthologues (SCOs) identified by Orthofinder across eight species. The phylogenetic tree from the concatenated SCOs of the eight species showed ruminants grouping and the *Bovidae* family in the same cluster (Figure 3).

DNA variants from aligning genome assemblies

There were on average, ~6 million SNVs discovered from pairwise genome alignments between swamp buffalo assemblies (Table 2) and, on average, ~7.4 million SNVs from pairwise comparisons of river buffalo assemblies. When a swamp assembly was aligned to a river

assembly, ~12 million SNVs were found on average. There were on average 23,138 structural variants (SVs) that were comprised of ~21 million bases found in pairwise comparisons of river buffalo assemblies. When a swamp assembly was aligned to a river assembly, 33,694 SVs that were made up of ~30 million bases were found on average. The river- and swamp-type buffalo divergence from autosomal SNP and SV is ~1.5%.

Most SVs detected in pairwise genome alignments were unique to each assembly with insertion, deletion and tandem expansions being more common than other types of SV (Figure 4; Figure 3_Supplementary Material). On average, ~14,000 SVs were unique to each assembly, which constituted ~15 Mb or 0.6% of the genome. There were 5,289 SVs that were shared by the three swamp buffalo assemblies when compared to the river buffalo reference (UOA_WB_1) (Figure 4_ Supplementary Material). In contrast, 4,981 SVs were shared by the river buffalo assemblies when compared to the swamp buffalo reference (PCC_UOA_SB_1v2).

Discovery of SNPs in buffaloes

The first phase of the 1000 Buffalo Genomes Project analyzed WGS data of 140 animals and identified a total of 41,632,997 and 41,071,165 SNPs using PCC_UOA_SB_1v2 and UOA_WB_1 as reference genomes, respectively (Table 3) with a Ti/Tv (transitions vs transversions) ratio of 2.12. An average of 25 million SNPs were identified for each buffalo type when selecting only the autosomes, biallelic loci, samples call rates >90% and SNPs call rates >90%. Out of the SNPs identified using the PCC_UOA_SB_1v2, ~14 million SNPs were river buffalo-specific whereas ~10 million SNPs were swamp buffalo-specific. When UOA_WB_1 was used as the reference, ~11 million SNPs were specific to river-type buffaloes and ~12 million SNPs were specific to swamp-type. Regardless of the reference genome choice, ~13 million SNPs with a minor allele frequency (MAF) >1% were shared between the two types and many of these can be considered ancestral variations.

Approximately 1.5 million SNPs were found to be polymorphic (MAF > 0.2) in swamp but were fixed in river buffaloes. Most of these variants were in the intergenic (~48%) and intronic (~36%) regions. Moreover, ~99% were SNPs classified as modifiers by snpEff, that were predicted to have minor impact as they are often found in non-coding regions. However, 0.24%, 0.43 and 0.01% have moderate, low, and high putative impact, respectively. The impacts were based on position in coding regions and type of amino acid changes. Among SNPs with predicted impact, 4,863 were non-synonymous mutations that affected 3,338 genes, which were polymorphic in swamp buffaloes but were fixed in river buffaloes. Of the 3,338 genes, 57 of them are associated with milk and reproductive traits (Table 13_Supplementary Material).

There were ~5 million SNPs in river that were fixed in swamp buffaloes. Of these SNPs, 36,890 were predicted to have an impact and 12,796 were non-synonymous mutations that affected 6,657 genes. Of these 6,657 genes, 130 genes were associated with milk production traits and reproductive traits (Table 14_Supplementary Material).

The average number of SNPs found in the short reads from the 140 samples were ~8 million SNPs and ~1 million InDels when using the reference genome from the same water buffalo type (Figure 5A; Table 15-16_Supplementary Material). The cumulative count of SNPs was lower when the sample and reference genome were from the same water buffalo type e.g. fewer SNPs were found for the Binhu breed, a swamp-type buffalo, when mapped to the swamp reference, PCC_UOA_SB_1v2, than to a river buffalo reference (Figure 6_Supplementary Material; Figure 7_Supplementary Material). Some SNPs were fixed within a subspecies, which would not be scored if the respective subspecies reference is used and are likely to be new mutations that occurred after the divergence of the buffalo sub-species from the common ancestor. A distinct genetic differentiation between the two water buffalo subspecies was observed. The PCA plot explained 34% of variation coming from ~3 million SNPs regardless of reference genome choice (Figure 5B; Figure 8_Supplementary Material). Swamp buffaloes display lower average heterozygosity per sample compared to river buffaloes (1.75 vs 1.88 heterozygous sites per kb).

Comparing the SNPs aligned with UOA_WB_1 to the 90K SNP buffalo genotyping array, about 26,890 SNPs were polymorphic only in river-type while 278 SNPs were polymorphic only in swamp-type (Figure 5C). Nevertheless, 39,000 SNPs were polymorphic in both river- and swamp-type. However, 55% of the SNPs in swamp-type have MAF < 0.1 (Figure 5D; Table 17_Supplementary Material). Only ~12,450 SNPs have MAF > 0.2 for swamp-type buffaloes in the 90K SNP array, which accounts for only ~17% of all the SNPs in the panel whereas 74% of the SNPs were highly polymorphic in river-type buffaloes.

Discussion

The accurate PacBio HiFi long-read sequences have facilitated the assembly of highly contiguous genomes including the human genome [26, 61]. Here, we presented a PacBio HiFi-based swamp buffalo genome assembly, which is more contiguous than other assemblies of the same species [10-13]. This Philippine swamp buffalo genome assembly has higher contig N50 (85.5 Mb vs 72.2 Mb), with fewer gaps (21 vs 140) and a higher Merqury QV score (45.8 vs 41.3) than the next best water buffalo genome [13]. It also exceeds other water buffalo genome assemblies [10-13] in the resolution of many types of repeats including telomeric and centromeric satellite sequences. The better resolution of repeats is likely to be the reason why our swamp buffalo assembly is larger than the other water buffalo assemblies. Genome assemblies that used HiFi reads, such as human [61], Hanwoo cattle [62] and sheep [63], also have larger genome sizes than previously published genome sizes for the same species. The satellite DNA sequences that we identified in the sub-metacentric (sat.673) and acrocentric (sat.1404) chromosomes are the same satellite repeats identified by two studies of water buffaloes [64, 65]. These repeats have ~80% similarity to the bovine satellite I and II sequences and are both localized in the centromeric regions of both water buffalo types [7]. We designated the second satellite repeat as sat.1404, instead of 1378 described in Pathak et al., 2006 [65] as the average length of the tandem repeats is 1,404 bp. The sat.673 repeats were found in all the water buffalo chromosomes [64, 65], however, we only found this satellite repeat in the sub-metacentric chromosomes and chromosome 9. There were no complete centromeres in any of our chromosomes, which was because the HiFi reads alone could not

completely span the repeats in centromeres. The quality of genome assemblies will improve as the accuracy of sequence reads, such as PacBio HiFi [66], and length of reads, such as Oxford Nanopore duplex [67] increases.

Here we also report the first phase analysis from 1000BGP on 140 water buffaloes, of which 60 are river buffaloes and 80 are swamp buffaloes, with DNA variants identified using the buffalo genomes from the two buffalo types as reference (UOA_WB_1 and PCC_UOA_SB_1v2). There were ~41 million SNPs discovered and the average number of heterozygous sites per individual was 1.81 per kilobase, which is higher than humans [68] and cattle [69]. The numbers of river- or swamp-specific SNPs were influenced by the choice of reference genomes, which could be due to read mapping bias in the reference genome [70].

The river buffaloes are more valued for their milk and have undergone a more organized breeding program compared to the swamp buffaloes. The river buffaloes have ~5 million SNPs that were fixed in the swamp buffaloes. One notable gene with a non-synonymous SNP (g.2754274C>T) is *DGAT1*, which is a well-known gene associated with milk production traits [71]. The SNP corresponds to *DGAT1* g.11,785 T > C in another study that reported the TC and TT genotypes associated with higher fat and protein percentages in milk, respectively [72]. Note the coordinates of the SNPs differ because they were discovered with different reference genomes. In swamp buffaloes, the SNP has a low frequency of the T allele (0.6%), whereas in river buffaloes the T allele frequency is higher at 21%. This difference in allele frequency could be the result of different selective pressures on milk fats. The g.2754274C>T SNP leads to a change in the protein sequence from alanine (Ala) to valine (Val) at position 494 (p.Ala494Val). This amino acid change has a moderate impact on the protein sequence. Several other genes with non-synonymous mutations e.g. *SASS6*, *VPS13B*, *ADGRA1*, *DNAH11*, *UBQLN4*, *PLEKHG7*, *ADAMTS9*, *DOCK7*, *ZNF292* and *AKAP6*, were candidate genes for milk yield [73-80].

We also found 22 and 9 genes with non-synonymous SNPs known to be linked with reproductive traits in the river buffaloes and swamp buffaloes, respectively. Among these genes, the *KISS1* and

KISS1R genes were associated with fertility traits in a gene expression study in ovarian follicular tissue in buffalo [81]. The *KISS1* encodes for the kisspeptin and *KISS1R* is the kisspeptin receptor, and they play a role in hormonal regulation that influences fertility traits such as gonadotropin releasing hormone (GnRH) and luteinizing hormone (LH) in ruminants [82]. The non-synonymous mutations have moderate impacts in *KISS1* (g,55887161G>A) and *KISS1R* (g.212308648C>A), which change the protein sequence from alanine to valine at position 133 (p.Ala133Val) and alanine to glutamic acid at position 36 (p.Ala36Glu), respectively. Among the polymorphic genes in river buffaloes that are fixed in swamp buffaloes, some genes such as *CAST* and CAPN have a strong association with meat tenderness in cattle [83] which could be the result of selective pressure for draft work in swamp buffaloes.

PCA analysis with ~3 million autosomal SNPs that were polymorphic in both buffalo types, clearly showed distinct genetic differentiation of river- and swamp-type buffaloes. The PCA plot (Figure 5B) shows a tight clustering of the swamp buffaloes and a loose clustering of the river buffaloes, which is similar to other water buffalo population studies using a 90K buffalo SNP panel [9, 84] and a high-density cattle SNP array [85]. Admixture analysis of river and swamp buffaloes by Sun et. al., 2020 [8] showed the distinctiveness of the Mediterranean breed and that introgression of the river type is evident in certain swamp buffaloes. This may be due in part to the interbreeding of the two types to improve milk production.

We estimated the divergence of the river and swamp buffalo to be between 2.6 to 4.9 million years ago (Mya), which is consistent with the 2.2 to 5.4 Mya divergence reported by Luo et al, 2020 [12]. Although natural mating between river- and swamp-type buffaloes is possible, it requires weeks to months for a riverine bull to socialize and successfully breed with swamp buffaloes. Furthermore, the two types of water buffalo do not live in the same natural environments and have only been present in the same geographical location recently due to the importation of the river-type buffaloes to Southeast Asia and Southern American countries to upgrade traits such as milk and meat production [86]. It is possible to generate fertile hybrids of river and swamp buffaloes [7] and as such, these two types of buffalo are still best defined as subspecies.

The genetic diversity captured in our dataset is sufficient for us to investigate the representativeness of SNP markers on the current 90K SNP array panel at genotyping river and swamp-type buffaloes. The current Axiom 90K Buffalo SNP array (Thermofisher) was created using data from river-type buffaloes, and the SNP showed high levels of heterozygosity in river buffaloes [18, 77, 87]. In the present study, 55% of SNPs in swamp buffalo samples detected in the 90K SNP array have MAF < 0.1. The SNP array was designed based on the polymorphism of four river buffalo breeds [18], so the limited performance of SNPs in swamp buffalo samples is unsurprising. We found 13 million SNPs from the first 1000BGP run that are polymorphic in both river and swamp buffaloes with MAF > 0.01. This SNP dataset presents an opportunity to design a genotyping panel suitable for both buffalo types. The SNP lists of this work are publicly available at the consortium's website found at https://1000buffalogenomes.github.io/datamgmt. The first and subsequent runs of 1000BGP SNP lists will be useful to those working on selection signatures, domestication signals [88], breed identification, screening for recessive lethal mutations [89] and many other uses.

In conclusion, we presented a high-quality swamp buffalo genome sequence that enabled analyses of genomic features missing from previous buffalo genome assemblies. There were distinct genetic differences between the river and swamp buffalo. We showed that reference genome choice affected the identification of genetic variants probably because it affected the alignment of short-read sequences. The first run of the 1000BGP identified a large number of SNPs including variants that were common between both types of buffalo for the design of a new genotyping SNP panel. In the future, the project aims to increase the data available on global water buffalo samples to increase information on water buffalo genetics. Further goals of the 1000BGP consortium are to create a buffalo pangenome graph using available long-read assemblies of different breeds and to generate phased telomere-to-telomere assemblies of a river x swamp buffalo hybrid to enable complete characterization of centromeres and other difficult to assemble genomic regions.

Figures

Figure 1. The female swamp buffalo from Kalinga Province, Philippines was selected for whole-genome assembly.

Figure 2. Comparison of gaps, major repeats, telomeric repeats and centromeric repeats compared to other assemblies. (A) Barplot of the number of gaps per chromosome displaying the low number of gaps of the PCC_UOA_SB_1v2. (B) Violin plot for swamp and river buffalo genome of repeat lengths >2 kb for LINE/L1, LINE/RTE-BovB and satellite/centromeric repeats. The boxplot inside shows the quartile range and median. (C) Barplot of the centromeric satellites repeat families found in the tentative centromeric region of each chromosome. (D) Bedgraph for the telomeric signals of the three highly contiguous water buffalo assemblies. Telomeric count is equal to one unit of TTAGGG/CCCTGG. The red arrow represents the possible misassembly in chromosome 1 of UOA_WB_1.

Figure 3. The phylogenetic tree of eight species using single-copy orthologue genes indicating estimated time divergence and confidence interval from the present in Mya.

Figure 4. Upset plot of the intersection of different types of structural variants (SV) identified in water buffalo assemblies when aligned to PCC_UOA_SV_1v2 (swamp type) which shows the number of shared and unique SVs between different water buffalo assemblies.

Figure 5. The first phase of the 1000 Buffalo Genomes Project. (A) Bar graph of the average number of SNPs with standard deviation in swamp and river buffalo aligned with PCC_UOA_SB_1v2 (swamp-type) and UOA_WB_1 (river-type). (B) Principal component analysis (PCA) plot using the swamp buffalo reference genome (PCC_UOA_SB_1v2) showing clear clustering of the swamp and river buffaloes. (C) Venn diagram of the number of autosomal SNPs in the 90K SNP buffalo genotyping array that are shared and specific for each water buffalo type. The large light peach circle shows the number of SNPs found in the 90K array, among these SNPs the dark peach color shows river-specific SNPs, the blue color shows swamp-specific SNPs and green shows SNPs shared for both types. (D) Histogram plot of the SNPs in the 90K SNP buffalo genotyping array including both specific and shared SNPs per MAF value binned at 0.01.

Tables

Table 1. Assembly metrics of the Philippine swamp buffalo and four water buffalo genome assemblies are available in public databases. For NDDB_SH_1, gaps were reported as 17.44 Mb in size for its scaffold assembly. For Wang_2023, the assembly size and number of sequences were only an estimation since they were not reported. NA denotes not available.

Assembly	Type	Assembly level	Assembly method	Assembly size (Gb)	N50 (Mb)	Number of sequences	Number of gaps	Reference
PCC_UOA_SB_1v2	Swamp	Contig	HiFiasm	2.95	85.5	500	0	This study
		Scaffold	YaHS	2.90	121.9	116	21	
		Chromosome	CombineFasta	2.70	121.9	24	20	
Wang_2023	Swamp	Contig	nextdenovo	2.68	72.2	173	0	Wang et al., 2023
		Scaffold	3d-dna	2.68	120.03	33	140	
		Chromosome	not specified	2.67	120.03	25	119	
UOA_WB_1	River	Contig	FALCON-Unzip	2.65	18.8	953	0	Low et al., 2019
		Scaffold	PacBio + Chicago + Hi-C	2.65	117.2	506	488	
		Chromosome	PBJelly, Arrow, Pilon	2.64	117.2	25	383	
NDDB_SH_	River	Contig	FALCON	2.62	9.5	1132	0	Ananthasay

1		Scaffold	Scaff10x + BioNano	2.63	82.0	59	NA	anam et al., 2020
		Chromosome	RaGOO	2.62	117.5	25	659	
CUSA_SWP	Swamp	Contig	Wtdbg	2.61	8.8	2003	0	Luo et al., 2020
		Scaffold	BioNano + HiC	2.63	117.3	1534	536	
		Chromosome	not specified	2.57	117.3	24	534	
CUSA_RVB	River	Contig	Wtdbg	2.63	3.1	3482	0	Luo et al., 2020
		Scaffold	BioNano + HiC	2.65	116.1	2304	1323	
		Chromosome	not specified	2.54	116.1	25	1323	

Table 2. Number of SNPs and size of SVs (bp) from pairwise genome assembly alignment. Numbers above "-" are the total size of structural variants (SVs) while below are the total number of SNPs.

	**Genome Assemblies**	**PCC_UOA_SB_1v2**	**Wang_2023**	**CUSA_SWP**	**UOA_WB_1**	**NDDB_SH_1**	**CUSA_RVB**
SNP	**PCC_UOA_SB_1v2**	-	17507727	21907568	27064859	27246061	32173283
	**Wang_2023**	6315498	-	22452637	27918711	28155313	32953777
	**CUSA_SWP**	5969757	5941882	-	29250364	29646973	33998313
	**UOA_WB_1**	12375163	12376399	11930093	-	16771137	23217345
	**NDDB_SH_1**	12437983	12470447	11984056	7999500	-	23562063
	**CUSA_RVB**	12093156	12082957	11896391	7758580	7771824	-

Table 3. Summary of SNP counts by reference genome and concordant SNPs with the 90K SNP buffalo genotyping array. Only the SNPs aligned with UOA_WB_1 were used to determine concordant SNPs in the 90K SNP buffalo genotyping array. Description for the SNPs rows was as follows: all = all SNPs found without filtering; autosomes only = all SNPs found in the autosomes without another filtering; swamp after QC = SNPs identified in swamp buffalo animals after quality filtering; river after QC = SNPs identified in river buffalo animals after quality filtering; swamp specific = SNPs identified only in the swamp (not in river) buffalo animals after quality filtering; river specific = SNPs identified only in the river (not in swamp) buffalo animals after quality filtering; and river and swamp shared = SNPs identified in both river and swamp buffalo animals after quality filtering.

SNPs	PCC_UOA_SB_1v2	UOA_WB_1	90K SNP buffalo array
all	41,632,997	41,071,165	90,000
autosomes only	40,905,045	40,340,557	72,434
swamp after QC	22,847,574	24,914,052	39,278
river after QC	26,525,477	24,485,667	65,890
swamp specific	10,161,461	11,756,460	278
river specific	13,839,364	11,328,075	26,890
river and swamp shared	12,686,113	13,157,592	39,000

Supplementary information

Supplementary Note 1. Further details on genome annotation

Full annotation of the swamp water buffalo is available in NCBI with annotation release ID GCF_029407905.1-RS_2023_04. The number of partial coding sequences (CDSs) and CDSs that required major corrections are indicators of the quality of genome annotation, the smaller the number, the better the quality. The PCC_UOA_SB_1v2 contains only 102 partial CDSs, fewer in comparison than the river water buffalo annotations: NDDB_SH_1 and UOA_WB_1 with 202 and 157 partial CDSs, respectively. There are also fewer CDSs with major corrections in the swamp

buffalo genome (~1% of the CDSs) compared to UOA_WB_1 (~3% of the CDSs). The improved sequence contiguity of the swamp buffalo has completely assembled the immunoglobulin heavy chain (IGH), a region mainly comprised of repeating sequences previously found in the unplaced scaffolds of the UOA_WB_1.

Supplementary Figures

Figure 1_Supplementary Material. Circos plot of swamp buffalo chromosome mapped to river buffalo. Chromosome 1 of the swamp buffalo showed clear homology to Chromosomes 4 and 9 of the river buffalo.

Figure 2_Supplementary Material. Genomescope2 profile showing k-mer spectra of the short-reads and inferring total genome length (len), percentage of the genome that are non-repetitive or unique (uniq), percentage of homozygosity (aa) and heterozygosity (ab), mean k-mer coverage for heterozygous bases (kcov), error rate of the reads (err), average rate of duplicate reads (dup), k-mer size used (k) and number of set of chromosomes (p).

Figure 3_Supplementary Material. An upset plot of the number of different types of structural variants (SV) identified when aligned to UOA_WB_1 (river type) which shows shared and unique SVs between various water buffalo assemblies.

Figure 4_Supplementary Material. Bar graph of the number of different types of structural variants (SV) shared between swamp buffalo assemblies (PCC_UOA_SB_1v2, Wang_2023 and CUSA_SWP) when aligned to river buffalo assembly (UOA_WB_1).

Figure 5_Supplementary Material. Bar graph of the number of the number of different types of structural variants (SV) shared between river buffalo assemblies (UOA_WB_1, NDDB_SH_1, CUSA_RVB) when aligned to swamp buffalo assembly (PCC_UOA_SB_1v2).

Figure 6_Supplementary Material. A line plot showing the cumulative number of SNPs of swamp-type buffalo samples per breed when aligned to swamp or river buffalo reference genomes.

Figure 7_Supplementary Material. A line plot showing the cumulative number of SNPs of river-type buffalo samples per breed when aligned to swamp or river buffalo reference genomes.

Figure 8_Supplementary Material. Principal component analysis (PCA) plot using the river buffalo reference genome (UOA_WB_1) shows clear clustering of the swamp and river buffaloes.

Table legends

Table 1_Supplementary Material. Software used in the study for de novo assembly, assessment, comparison, and analysis.

Table 2_Supplementary Material. Estimated satellite arrays of the human T2T genome assembly using repeatmasker. The T2T-CHM13v1 column is from Table 5 of Nurk et al., 2022 showing coordinates of alpha and human satellite arrays in v1.0 assembly.

Table 3_Supplementary Material. Data accession number and links for species used in the estimation of divergence.

Table 4_Supplementary Material. Whole-genome short-read sequence information on the samples for the 1000 Buffalo Genomes Project.

Table 5_Supplementary Material. List of article searches for water buffalo genomes.

Table6_Supplementary Material. Sequencing reads.

Table 7_Supplementary Material. Assembly statistics.

Table 8_Supplementary Material. Percentage of repeat sequences and length of repeat families in the water buffalo assemblies. Repeat alignment lengths less than 2.5 Kbp were filtered out. Numbers are in base pair (bp).

Table 9_Supplementary Material. Sizes in base pairs (bp) of the satellite repeat types within the estimated centromeric region per chromosome of the Philippine swamp genome. The repeat types sat.1404 and sat.673 are a subset of the repeat families identified by repeat masker.

Table 10_Supplementary Material. Number of telomeric repeats across five water buffalo assemblies within a 20kbp window of each end of the chromosomes. Telomere counts less than 50 were filtered out. One telomeric repeat is equivalent to TTAGGG1.

Table 11_Supplementary Material. Comparisons of various assembly features of the water buffalo genome assemblies available in NCBI. The Male swamp buffalo, Fuzhong swamp buffalo and Murrah river buffalo are annotated differently. NA denotes not available.

Table 12_Supplementary Material. Estimated divergence time and confidence interval of the eight species.

Table 13_Supplementary Material. List of genes polymorphic in swamp buffaloes but is fixed in river buffaloes with corresponding traits from research articles on water buffaloes.

Table 14_Supplementary Material. List of genes polymorphic in river buffaloes but is fixed in swamp buffaloes with corresponding traits from research articles on water buffaloes.

Table 15_Supplementary Material. Number of SNPs, InDels and cumulative SNPs of swamp buffaloes per sample using swamp and river reference genomes.

Table 16_Supplementary Material. Number of SNPs, InDels and cumulative SNPs of river buffaloes per sample using swamp and river reference genomes.

Table 17_Supplementary Material. Number of SNPs per MAF range with intervals of 0.1 using swamp and river reference genomes, and SNPs concordance with the 90K SNP buffalo genotyping array.

Data availability

The PacBio HiFi reads, Hi-C reads, and Illumina paired-end reads are available in the SRA under BioProject PRJNA901059. The BioSample of the animal is SAMN31703457. The genome accession number for PCC_UOA_SB_1v2 is GCA_029407905.2. The assemblies UOA_WB_1 (GCA_003121395.1) and NDDB_SH_1 (GCA_019923935.1) were downloaded from NCBI. The assemblies CUSA_SWP (GWHAAJZ00000000) and CUSA_RVB (GWHAAKA00000000) were downloaded in NGDC. The assembly Wang_2023 was downloaded from Figshare [90] as stated in Wang et al., 2023. Annotation files are available through NCBI with RefSeq GCF_029407905.1. All additional supporting data, including the identified variants and scripts produced in this study, are available in the GigaScience database, GigaDB [91].

Acknowledgments

This work was supported with supercomputing resources provided by the Phoenix HPC service at the University of Adelaide. The work was partly funded by the Philippine Carabao Center. We thank the Kalinga Province Veterinary Local Government Unit, Sherwin Matias, and Maureen Gajeton for assisting with sample collection. The work was supported in part by funds from USDA-ARS. The use of trade names or commercial products in this manuscript is solely to provide specific

information. It does not imply recommendation or endorsement by the U.S. Department of Agriculture. USDA is an equal opportunity provider and employer. We thank Francoise Thibaud-Nissen for her help in coordinating genome annotation at the NCBI. We also thank the DOST-SEI Foreign Graduate Scholarship program for providing financial assistance to P.S.P.

Author contributions

The genome assembly study was jointly conceived by P.S.P., E.B.F., L.P.V., T.P.L.S., and W.Y.L. Additionally, P.S.P., E.B.F., M.S.K., and W.Y.L. jointly conceived the buffalo consortium. Genome sequencing and base calling were contributed by T.P.L.S. and B.D.R. Coordination of short-read sequence data and ideas for the 1000BGP were contributed by C.J.P., P.A.M., L.C., and J.L.W. P.S.P. conducted the genome assembly and downstream analysis, while W.Y.L. handled SNP calling. Divergence time estimation was performed by T.H.T. and P.S.P. The initial manuscript was written by P.S.P. and W.Y.L., with revisions provided by L.P.V., M.S.K., T.P.L.S., B.D.R., L.C., and J.L.W.

Competing interests

The authors declare no competing interests.

REFERENCES

1. Maylem, E.R.S., et al., Development of adaptability of foreign breeds of water buffalo in Philippine tropical climate. Anim Front, 2023. 13(5): p. 89-91.
2. Minervino, A.H.H., et al., Bubalus bubalis: A Short Story, in Frontiers in Veterinary Science. 2020.
3. FAO, World Watch List for Domestic Animal Diversity. 2000 (FAO, Rome).
4. Pineda, P.S., et al., Opportunities and Challenges for Improving the Productivity of Swamp Buffaloes in Southeastern Asia. Frontiers in Genetics, 2021. 12(March): p. 1-8.

5. Escarcha, J.F., et al., Livelihoods transformation and climate change adaptation: The case of smallholder water buffalo farmers in the Philippines. Environmental Development, 2020. 33(September 2018): p. 100468-100468.
6. Degrandi, T., et al., Cytogenetic identification of four generations of crossbred buffaloes maintained in a conservation program in the Marajó island/Brazil. Journal of Biotechnology and Biodiversity, 2014: p. 162-171.
7. Iannuzzi, A., P. Parma, and L. Iannuzzi, The cytogenetics of the water buffalo: A review. Animals, 2021. 11(11).
8. Sun, T., et al., Genomic analyses reveal distinct genetic architectures and selective pressures in buffaloes. GigaScience, 2020. 9(2).
9. Colli, L., et al., New insights on water buffalo genomic diversity and post-domestication migration routes from medium density SNP chip data. Frontiers in Genetics, 2018. 9(MAR).
10. Ananthasayanam, S., et al., First near complete haplotype phased genome assembly of River buffalo (*Bubalus bubalis*). bioRxiv, 2020: p. 618785-618785.
11. Low, W.Y., et al., Chromosome-level assembly of the water buffalo genome surpasses human and goat genomes in sequence contiguity. Nature Communications, 2019. 10(1): p. 1-11.
12. Luo, X., et al., Understanding divergent domestication traits from the whole-genome sequencing of swamp- And river-buffalo populations. National Science Review, 2020. 7(3): p. 686-701.
13. Wang, X., et al., Chromosome-level genome and recombination map of the male buffalo. GigaScience, 2023. 12.
14. Li, H. and R. Durbin, Genome assembly in the telomere-to-telomere era. Nat Rev Genet, 2024.
15. Aganezov, S., et al., A complete reference genome improves analysis of human genetic variation. Science, 2022. 376(6588).
16. The Vertebrate Genomes Project, A reference standard for genome biology. Nature Biotechnology, 2018. 36(1121).
17. Liang, D., et al., Genomic Analysis Revealed a Convergent Evolution of LINE-1 in Coat Color: A Case Study in Water Buffaloes (Bubalus bubalis). Mol Biol Evol, 2021. 38(3): p. 1122-1136.

18. Iamartino, D., et al., Design and validation of a 90K SNP genotyping assay for the water buffalo (Bubalus bubalis). PLOS ONE, 2017. 12(10): p. e0185220-e0185220.
19. Herrera, J.R., et al. Genome-wide association study for milk traits in Philippine dairy buffaloes. 2018.
20. The 1000 Buffalo Genomes Project, 2024. https://1000buffalogenomes.github.io/.
21. Villamor, L., et al., Genetic Diversity of Philippine Carabao (Bubalus bubalis) Using Mitochondrial DNA D-loop Variation: Implications to Conservation and Management. Philippine Journal of Science, 2021. 150.
22. Krueger, F., TrimGalore. 2016. https://github.com/FelixKrueger/TrimGalore.
23. Andrews, S., FastQC - A quality control tool for high throughput sequence data. http://www.bioinformatics.babraham.ac.uk/projects/fastqc. Babraham Bioinformatics, 2010.
24. Baid, G., et al., DeepConsensus improves the accuracy of sequences with a gap-aware sequence transformer. Nature Biotechnology, 2022.
25. Sim, S.B., et al., HiFiAdapterFilt, a memory efficient read processing pipeline, prevents occurrence of adapter sequence in PacBio HiFi reads and their negative impacts on genome assembly. BMC Genomics, 2022. 23(1).
26. Cheng, H., et al., Haplotype-resolved de novo assembly using phased assembly graphs with hifiasm. Nature Methods, 2021. 18(2).
27. Li, H., Minimap and miniasm: Fast mapping and de novo assembly for noisy long sequences. Bioinformatics, 2016. 32(14).
28. Guan, D., et al., Identifying and removing haplotypic duplication in primary genome assemblies. Bioinformatics. 36(9): p. 2896-2898.
29. Arima Genomics, *mapping_pipeline.* 2024. https://github.com/ArimaGenomics/mapping_pipeline.
30. Zhou, C., S.A. McCarthy, and R. Durbin, YaHS: yet another Hi-C scaffolding tool. Bioinformatics (Oxford, England), 2023. 39(1): p. 10-12.
31. Rosen, B.D., et al., De novo assembly of the cattle reference genome with single-molecule sequencing. GigaScience, 2020. 9(3).

32. Jain, C., et al., Weighted minimizer sampling improves long read mapping. Bioinformatics, 2020. 36.
33. Durand, N.C., et al., Juicer Provides a One-Click System for Analyzing Loop-Resolution Hi-C Experiments. Cell Systems, 2016. 3(1).
34. Durand, N.C., et al., Juicebox Provides a Visualization System for Hi-C Contact Maps with Unlimited Zoom. Cell Systems, 2016. 3(1).
35. Krumsiek, J., R. Arnold, and T. Rattei, Gepard: A rapid and sensitive tool for creating dotplots on genome scale. Bioinformatics, 2007. 23(8).
36. Bickhart, D., *CombineFasta.* 2020. https://github.com/njdbickhart/CombineFasta.
37. Escalona, M., *YagCloser.* 2023. https://github.com/merlyescalona/yagcloser.
38. Ranallo-Benavidez, T.R., K.S. Jaron, and M.C. Schatz, GenomeScope 2.0 and Smudgeplot for reference-free profiling of polyploid genomes. Nature Communications, 2020. 11(1).
39. Rhie, A., et al., Merqury: Reference-free quality, completeness, and phasing assessment for genome assemblies. Genome Biology, 2020. 21(1).
40. Gurevich, A., et al., QUAST: Quality assessment tool for genome assemblies. Bioinformatics, 2013. 29(8).
41. Simão, F.A., et al., BUSCO: Assessing genome assembly and annotation completeness with single-copy orthologs. Bioinformatics, 2015. 31(19): p. 3210-3212.
42. Uliano-Silva, M., et al., MitoHiFi: a python pipeline for mitochondrial genome assembly from PacBio high fidelity reads. BMC Bioinformatics, 2023. 24(1).
43. Camacho, C., et al., BLAST+: Architecture and applications. BMC Bioinformatics, 2009. 10.
44. Smit, A.F.A., R. Hubley, and P. Green, RepeatMasker Open-3.0, in RepeatMasker Open-3.0. 1996.
45. Brown, M., González De la Rosa, P. M. and Mark, B., A Telomere Identification Toolkit. Zenodo, 2023. https://github.com/tolkit/telomeric-identifier.
46. Benson, G., Tandem repeats finder: A program to analyze DNA sequences. Nucleic Acids Research, 1999. 27(2).
47. Gao, S., et al., HiCAT: a tool for automatic annotation of centromere structure. Genome Biology, 2023. 24(1).

48. The NCBI Eukaryotic Genome Annotation Pipeline, 2024. https://www.ncbi.nlm.nih.gov/refseq/annotation_euk/process/. Accessed 5 May 2024.
49. Minh, B.Q., et al., IQ-TREE 2: New Models and Efficient Methods for Phylogenetic Inference in the Genomic Era. Molecular Biology and Evolution, 2020. 37(5).
50. Yang, Z., PAML 4: Phylogenetic analysis by maximum likelihood. Molecular Biology and Evolution, 2007. 24(8).
51. Emms, D.M. and S. Kelly, OrthoFinder: Phylogenetic orthology inference for comparative genomics. Genome Biology, 2019. 20(1).
52. To, T.H., et al., Fast Dating Using Least-Squares Criteria and Algorithms. Systematic Biology, 2016. 65(1).
53. Rannala, B. and Z. Yang, Inferring speciation tunes under an episodic molecular clock. Systematic Biology, 2007. 56(3).
54. Benton, M., et al., Constraints on the timescale of animal evolutionary history. Palaeontologia Electronica, 2015. 18: p. 1-116.
55. Marçais, G., et al., MUMmer4: A fast and versatile genome alignment system. PLoS Computational Biology, 2018. 14(1).
56. Nattestad, M. and M.C. Schatz, Assemblytics: A web analytics tool for the detection of variants from an assembly. Bioinformatics, 2016. 32(19).
57. Poplin, R., et al., Scaling accurate genetic variant discovery to tens of thousands of samples. 2017. bioRxiv 201178 . https://doi.org/10.1101/201178.
58. Li, H., A statistical framework for SNP calling, mutation discovery, association mapping and population genetical parameter estimation from sequencing data. Bioinformatics, 2011. 27(21): p. 2987-93.
59. Purcell, S., et al., PLINK: a tool set for whole-genome association and population-based linkage analyses. Am J Hum Genet, 2007. 81(3): p. 559-75.
60. Cingolani, P., et al., A program for annotating and predicting the effects of single nucleotide polymorphisms, SnpEff: SNPs in the genome of Drosophila melanogaster strain w1118; iso-2; iso-3. Fly (Austin), 2012. 6(2): p. 80-92.
61. Nurk, S., et al., The complete sequence of a human genome. Science, 2022. 376(6588).

62. Jang, J., et al., Chromosome-level genome assembly of Korean native cattle and pangenome graph of 14 Bos taurus assemblies. Scientific Data, 2023. 10(1).
63. Li, R., et al., A sheep pangenome reveals the spectrum of structural variations and their effects on tail phenotypes. Genome Research, 2023. 33(3).
64. Tanaka, K., et al. Characterization and chromosomal distribution of satellite DNA sequences of the water buffalo (Bubalus bubalis). in Journal of Heredity. 1999.
65. Pathak, D., et al., Chromosomal localization, copy number assessment, and transcriptional status of BamHI repeat fractions in water buffalo Bubalus bubalis. DNA and Cell Biology, 2006. 25(4).
66. Wenger, A.M., et al., Accurate circular consensus long-read sequencing improves variant detection and assembly of a human genome. Nature Biotechnology, 2019. 37(10): p. 1155-1162.
67. Oxford Nanopore Technologies., Improved de novo assembly with nanopore ultra-long and duplex data, and scaffolding using Pore-C. 2023. https://nanoporetech.com/resource-centre/improved-de-novo-assembly-nanopore-ultra-long-and-duplex-data-and-scaffolding-using. Accessed 23 November 2023.
68. Altshuler, D.L., et al., A map of human genome variation from population-scale sequencing. Nature, 2010. 467(7319).
69. Daetwyler, H.D., et al., Whole-genome sequencing of 234 bulls facilitates mapping of monogenic and complex traits in cattle. Nature Genetics, 2014. 46(8).
70. Valiente-Mullor, C., et al., One is not enough: On the effects of reference genome for the mapping and subsequent analyses of short-reads. PLoS Computational Biology, 2021. 17(1).
71. Khan, M.Z., et al., Association of DGAT1 With Cattle, Buffalo, Goat, and Sheep Milk and Meat Production Traits. Front Vet Sci, 2021. 8: p. 712470.
72. de Freitas, A.C., et al., Genetic association between SNPs in the DGAT1 gene and milk production traits in Murrah buffaloes. (1573-7438 (Electronic)).
73. Deng, T., et al., Integrative Analysis of Transcriptome and GWAS Data to Identify the Hub Genes Associated With Milk Yield Trait in Buffalo. Front Genet, 2019. 10: p. 36.

74. Liu, J.J., et al., Genome-wide association studies to identify quantitative trait loci affecting milk production traits in water buffalo. J Dairy Sci, 2018. 101(1): p. 433-444.
75. Abdel-Shafy, H., et al., Prospecting genomic regions associated with milk production traits in Egyptian buffalo. J Dairy Res, 2020. 87(4): p. 389-396.
76. de Camargo, G.M., et al., Prospecting major genes in dairy buffaloes. BMC Genomics, 2015. 16: p. 872.
77. Mokhber, M., et al., Study of whole genome linkage disequilibrium patterns of Iranian water buffalo breeds using the Axiom Buffalo Genotyping 90K Array. PLoS ONE, 2019. 14(5).
78. Vohra, V., et al., Genome-Wide Association Studies in Indian Buffalo Revealed Genomic Regions for Lactation and Fertility. Front Genet, 2021. 12: p. 696109.
79. Ravi Kumar, D., et al., Genomic diversity and selection sweeps identified in Indian swamp buffaloes reveals it's uniqueness with riverine buffaloes. Genomics, 2020. 112(3): p. 2385-2392.
80. Lazaro, S.F., et al., Genomic studies of milk-related traits in water buffalo (Bubalus bubalis) based on single-step genomic best linear unbiased prediction and random regression models. J Dairy Sci, 2021. 104(5): p. 5768-5793.
81. Mishra, G., et al., Relative expression profile of Kisspeptin (Kiss1-Kiss1r) and gonadotrophin receptor in the ovarian follicular tissue and their association in the buffalo. The Indian journal of animal sciences, 2022. 92: p. 580-584.
82. Daniel, J.A., et al., Reproduction and beyond, kisspeptin in ruminants. J Anim Sci Biotechnol, 2015. 6(1): p. 23.
83. Kostusiak, P.A.-O., et al., Polymorphism of Genes and Their Impact on Beef Quality. (1467-3045 (Electronic)).
84. Herrera, J.R., et al., Performance of the Axiom 90k Buffalo Genotyping Array in four Philippine water buffalo populations. Revista CES Medicina Veterinaria y Zootecnia, 2016. 11: p. 210-210.
85. Pérez-Pardal, L., et al., Genomic differentiation between swamp and river buffalo using a cattle high-density single nucleotide polymorphisms panel. Animal, 2017. 12(3): p. 464-471.

86. Cruz, L.C., Changing faces of swamp buffaloes in an industrializing Asia. Buffalo Bulletin, 2013. 32(SPEC. ISSUE 1): p. 32-49.
87. Herrera, J.R.V., et al., Accuracy of Genomic Prediction for Milk Production Traits in Philippine Dairy Buffaloes, in Frontiers in Genetics. 2021. p. 1996-1996.
88. Dutta, P., et al., Whole genome analysis of water buffalo and global cattle breeds highlights convergent signatures of domestication. Nature Communications, 2020. 11(1).
89. VanRaden, P.M., et al., Harmful recessive effects on fertility detected by absence of homozygous haplotypes. J Dairy Sci, 2011. 94(12): p. 6153-61.
90. Wang, XB., The genome and annotation of the male swamp buffalo. Figshare, 2023. https://doi.org/10.6084/m9.figshare.19885720.v2 .
91. Pineda P, F.E., Villamor LP, Parac CJ, Khatkar MS, Thu HT, et al., Supporting data for "Disentangling river- and swamp-buffalo genetic diversity: Initial Insights from the 1000 Buffalo Genomes Project". GigaScience Database, 2024. https://doi.org/10.5524/102562 .

List of members of the 1000 Buffalo Genomes Consortium

Name	Institution	Country	Contact
Lloyd Low	The University of Adelaide	Australia	wai.low@adelaide.edu.au
Mehar Khatkar	The University of Adelaide	Australia	mehar.khatkar@adelaide.edu.au
Tong Chen	The University of Adelaide	Australia	tong.chen@adelaide.edu.au
Hanh Thi Hong Nguyen	University of Adelaide	Australia	hanh.t.nguyen@adelaide.edu.au
Humberto Tonhati	Universidade Estadual Paulista	Brasil	humberto.tonhati@unesp.br
Gregório Miguel Ferreira de Camargo	Escola de Medicina Veterinária e Zootecnia	Brasil	gregorio.camargo@ufba.br
Stefano Biffani	IBBA-CNR National Research Council	Brasil	biffani@ibba.cnr.it
Jianlin, Han	Consultative Group on International Agricultural Research	China	h.jianlin@cgiar.org
Yi Zhang	China Agricultural University	China	yizhang@cau.edu.cn
Mei Liu	Hunan Agricultural University	China	mei.liu@hunau.edu.cn
Yang Zhou	Huazhong Agricultural University	China	yangzhou@mail.hzau.edu.cn
Divier Antonio Agudelo Gómez	Universidad CES	Columbia	dagudelo@ces.edu.co

P. Kumarasamy	Tamil Nadu Veterinary and Animal Sciences University	India	pksamy2000@gmail.com
Jaswinder Singh Bhatti	Progressive Dairy Farmers Association	India	drjsbhatti@gmail.com
Manishi Mukesh	ICAR-National Bureau of Animal Genetic Resources	India	mmukesh_26@hotmail.com
Dwi Sendi Priyono	Universitas Gadjah Mada	Indonesia	dwisendipriyono@ugm.ac.id
Akhmad Dakhlan	Universitas Lampung	Indonesia	akhmad.dakhlan@fp.unila.ac.id
Mahdi Mokhber	Urmia University	Iran	mehdi.mokhber@ut.ac.ir
John Williams	Università Cattolica del Sacro Cuore	Italy	john.williams01@adelaide.edu.au
Ajmone Marsan Paolo	Catholic University of the Sacred Heart	Italy	paolo.ajmone@unicatt.it
Licia Colli	Catholic University of the Sacred Heart	Italy	licia.colli@unicatt.it
Mayra Gómez Carpio	ANASB (Italian National Association of Buffalo Breeders)	Italy	m.gomezcarpio@anasb.it
Roberta Cimmino	ANASB (Italian National Association of Buffalo Breeders)	Italy	r.cimmino@anasb.it
Ali Raza Awan	University of Veterinary and Animal Sciences, Lahore	Pakistan	arawan77@uvas.edu.pk
Paulene S. Pineda	The University of Adelaide, Philippine Carabao Center	Philippines	paulene.pineda@adelaide.edu.au
Lilian P. Villamor	Philippine Carabao Center	Philippines	lpvillamor2021@gmail.com
Ester B. Flores	Philippine Carabao Center	Philippines	esterflrs@gmail.com
Connie Joyce Parac	Philippine Carabao Center	Philippines	cjparac@gmail.com
Rangsun Parnpai	Suranaree University of Technology	Thailand	rangsun@g.sut.ac.th
Siri Tuk	Department of Livestock Development	Thailand	tuk_siri@yahoo.com
M.İhsan Soysal	Tekirdag Namık Kemal University	Turkey	misoysal@gmail.com; misoysal@nku.edu.tr
Emel Özkan Unal	Tekirdag Namık Kemal University	Turkey	ozemel@nku.edu.tr
Raziye Isik	Tekirdag Namık Kemal University	Turkey	risik@nku.edu.tr
Zhihua Jiang	Washington State University	USA	jiangz@wsu.edu
Đỗ Đức Lực	Vietnam National University	Vietnam	ddluc@vnua.edu.vn
Nguyen Hoang Thinh	Vietnam National University	Vietnam	nhthinh@vnua.edu.vn

Figure 1

Click here to access/download;Figure;Figure1.pdf

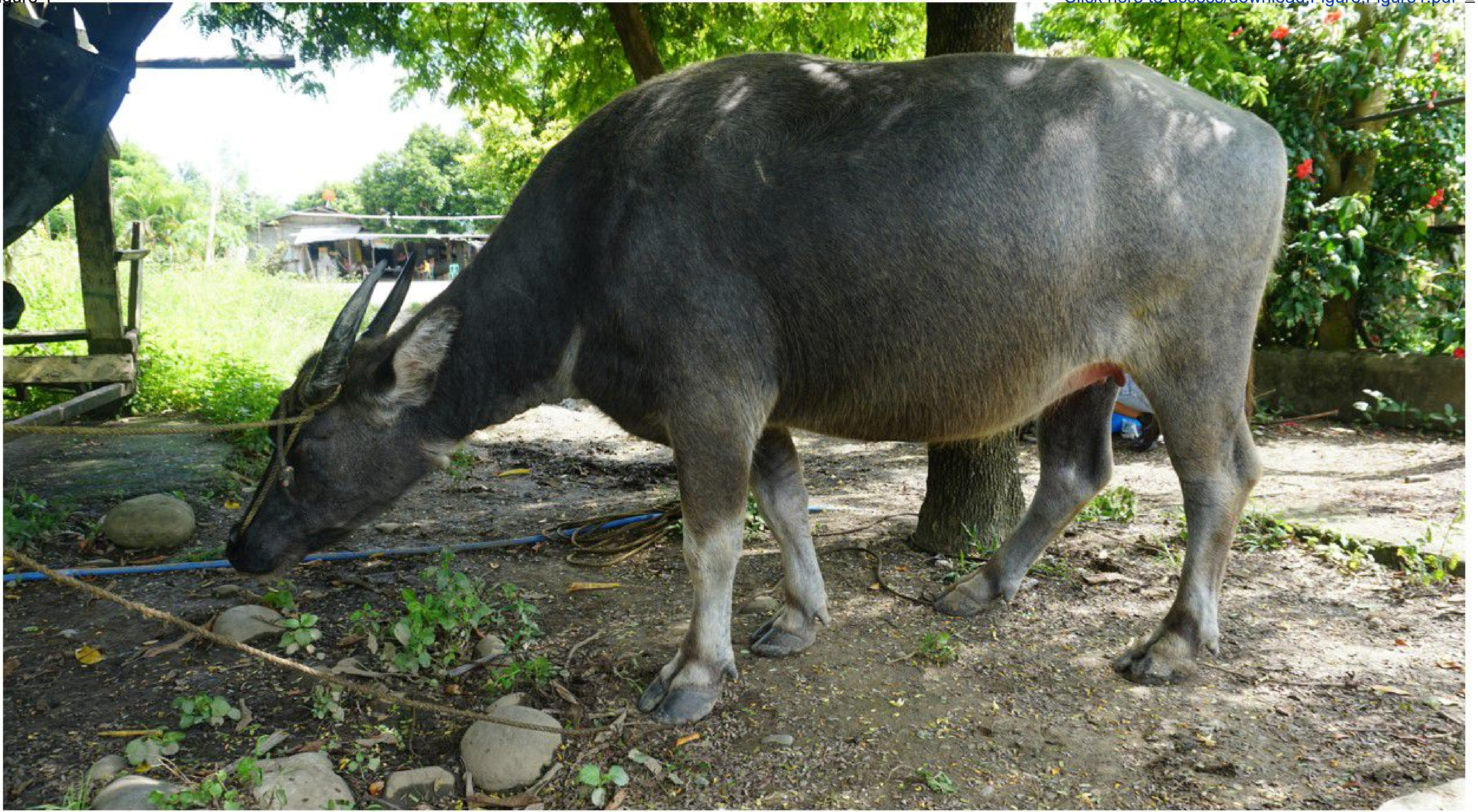

Figure 2

Click here to access/download;Figure;Figure2.pdf

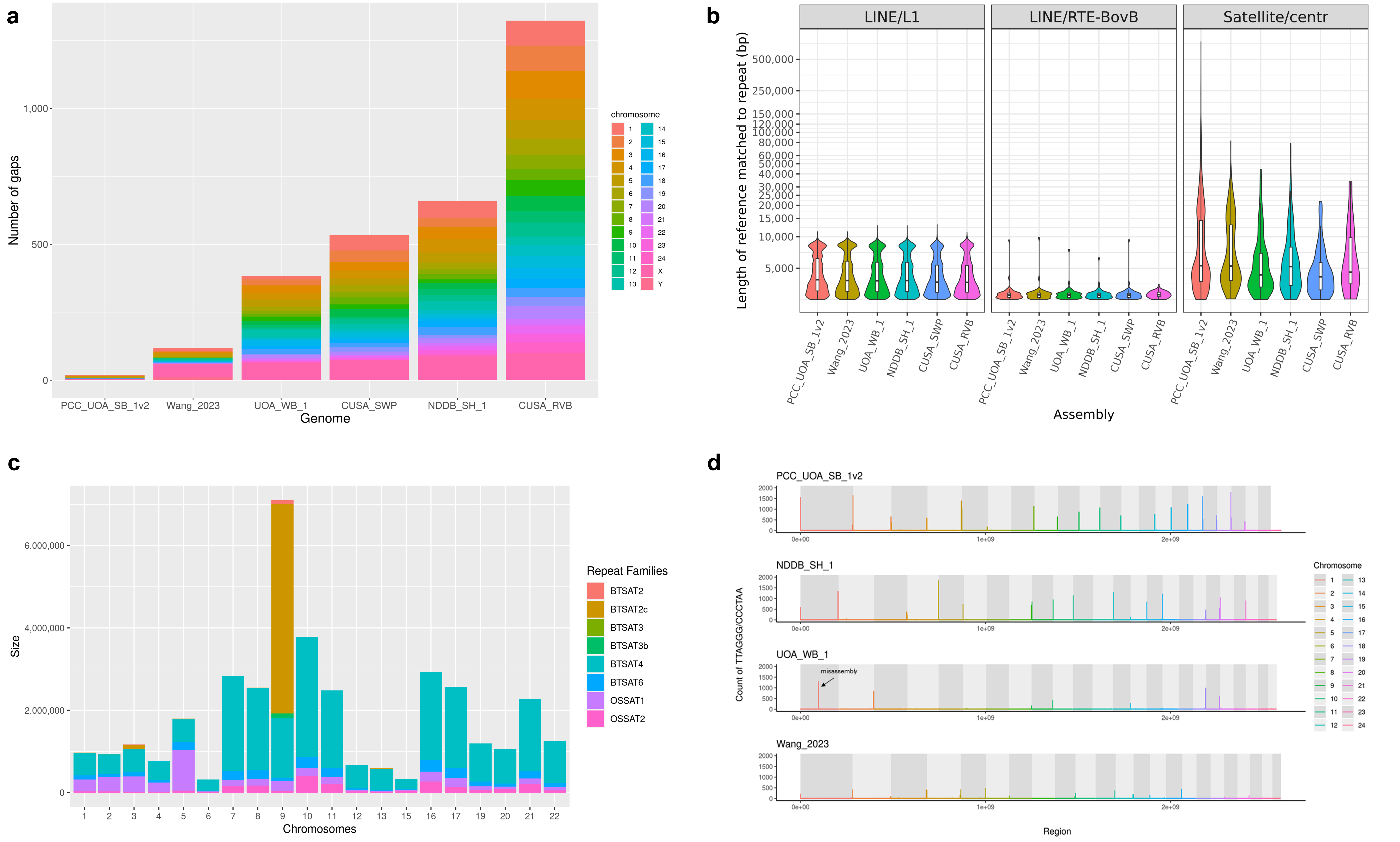

Figure 3

Click here to access/download;Figure;Figure3.pdf

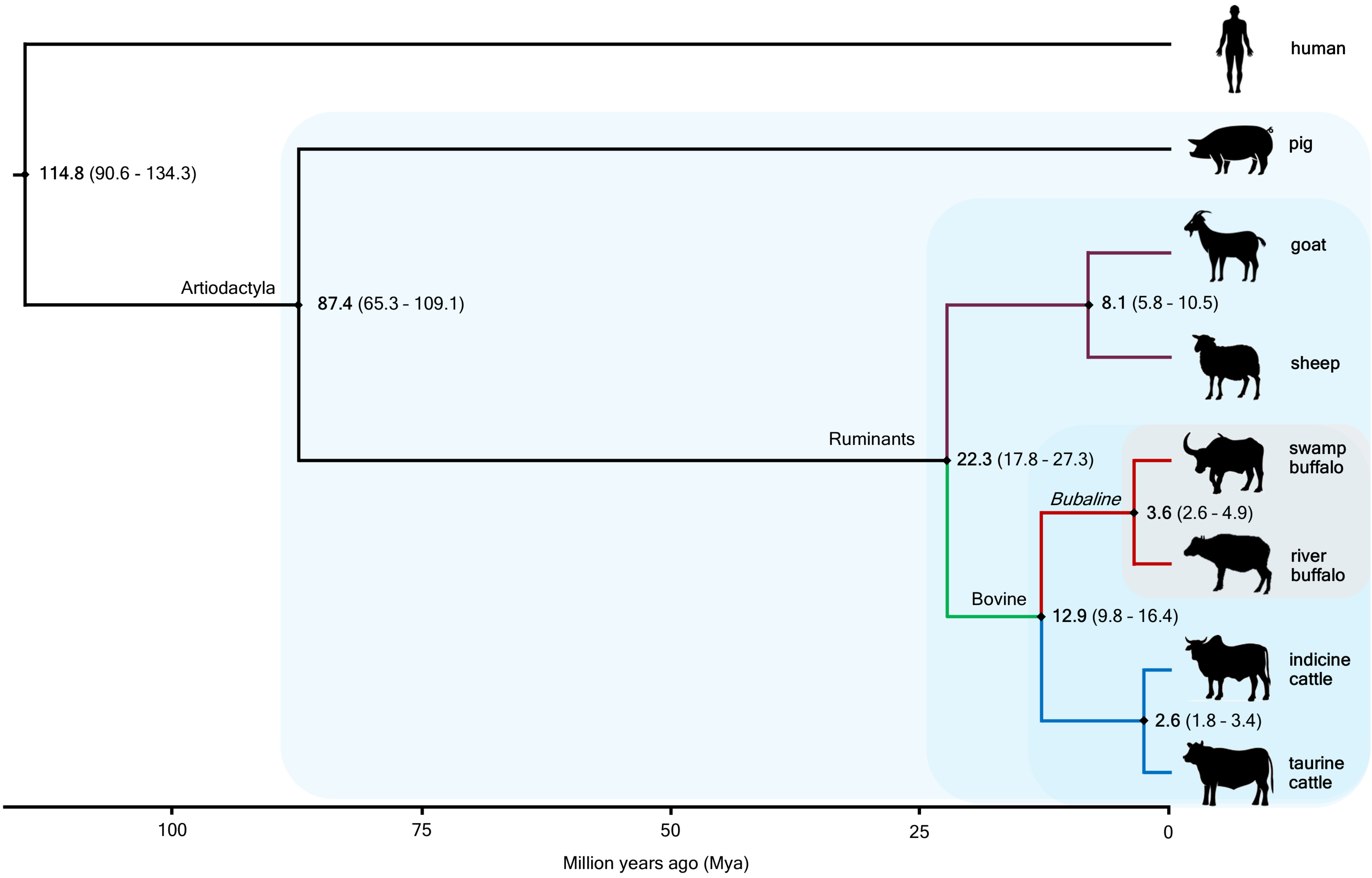

Figure 4

Click here to access/download;Figure;Figure4.pdf

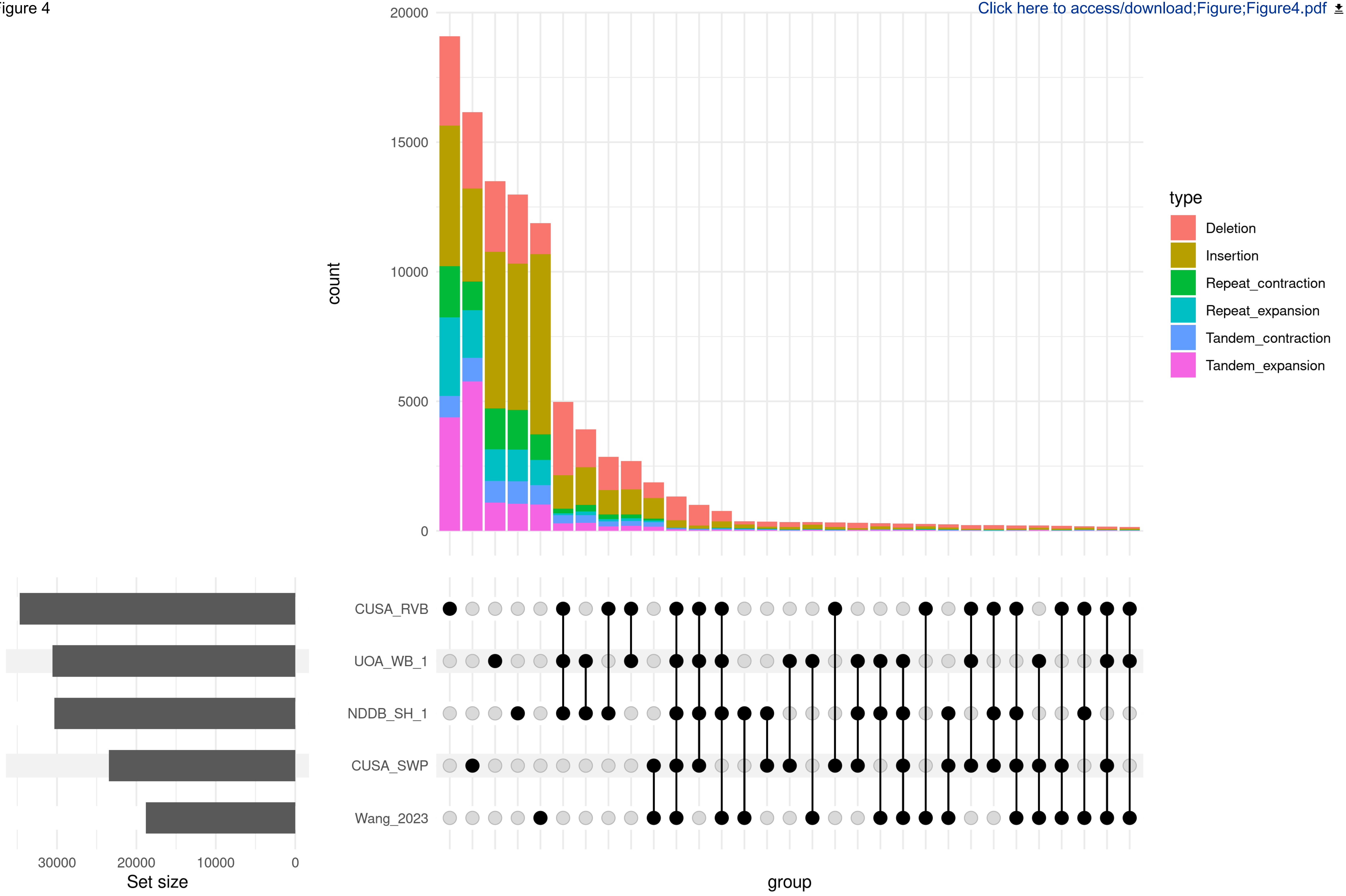

Figure 5

Click here to access/download;Figure;Figure5.pdf

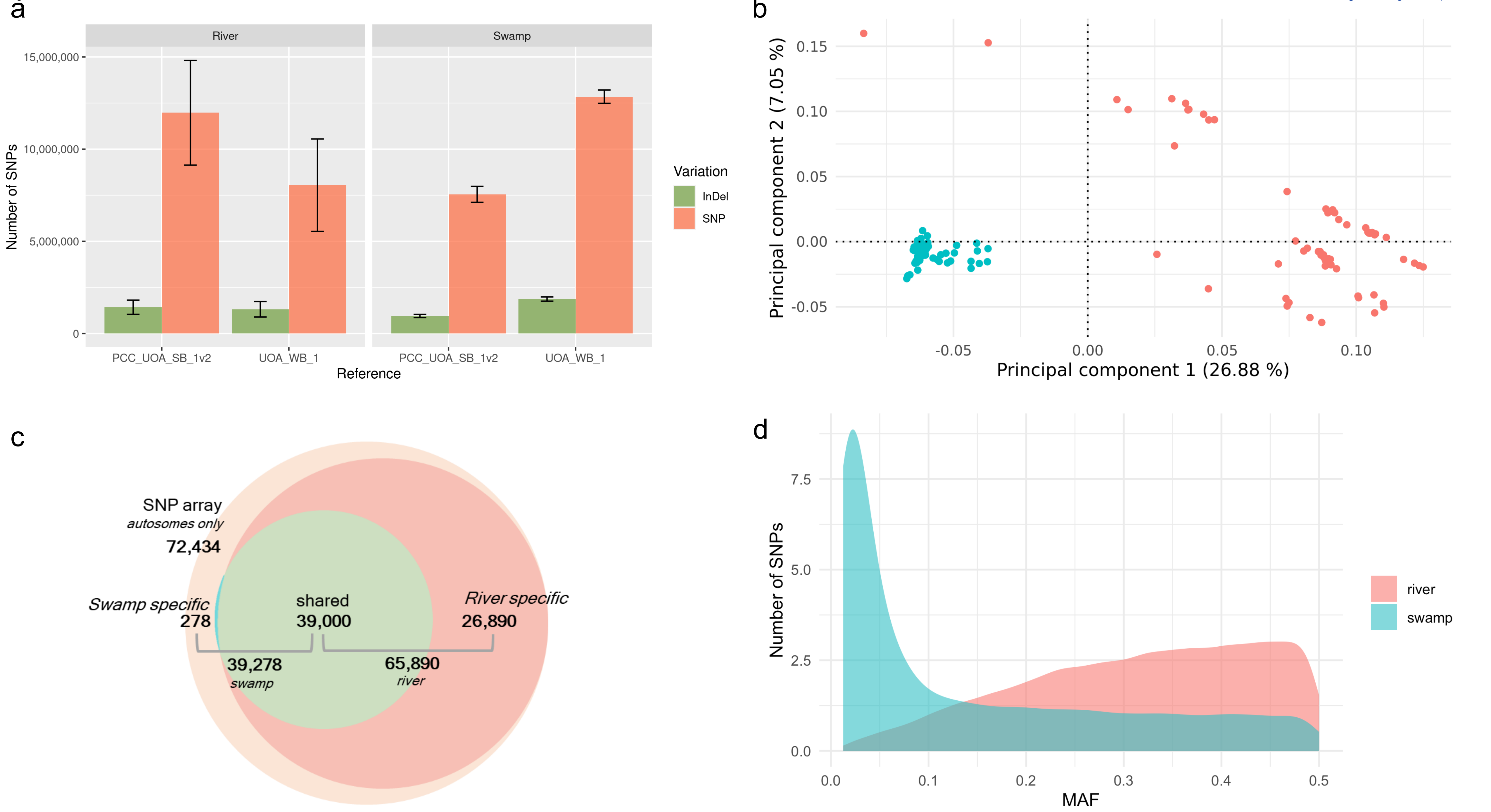

Supplementary Figures

Click here to access/download
Supplementary Material
supplementary_figures.docx

Supplementary Tables

Click here to access/download
Supplementary Material
Supplementary_tables.xlsx

Cover letter

Click here to access/download;Personal Cover;cover_letter_1000BGP_giga_20240318.pdf

THE UNIVERSITY of ADELAIDE

Dear Editor,

We submit an original manuscript, "Disentangling river- and swamp-buffalo genetic diversity: Initial Insights from the 1000 Buffalo Genomes Project", to be considered for publication as an Article in *Gigascience*. The paper describes the world's largest genomics study on water buffalo, encompassing two subspecies: river-type and swamp-type buffalo. The **1000 Buffalo Genome Project (1000BGP)** (https://1000buffalogenomes.github.io/) is an international consortium that is made up of 38 members of researchers who work on water buffalo from 15 countries. There are many goals within the 1000BGP and here we present two of the goals, which is creation of high-quality reference genomes for both water buffalo subspecies and generation of single nucleotide polymorphisms (SNPs) data to guide genetic studies of the species. A river buffalo reference genome has been made available by the principal investigator of this project in 2019. In this paper, **a new swamp buffalo genome** is described, and its quality has surpassed all available water buffalo assemblies. The **SNPs data** were generated from 140 samples that consisted of both river- and swamp-type buffalo, which is referred to as the first run of the 1000BGP. To discover and compare these SNPs, we have mapped short read data from the 140 samples to both subspecies' reference genomes, which is a novel aspect in buffalo genomics study.

To summarise some of our key findings, we have presented a high-quality genome sequence for the swamp buffalo, providing **insights into genomic features** such as centromeric and telomeric repeats previously absent in other buffalo genome assemblies. Our analysis revealed **distinct genetic differences between river and swamp buffalo**, with SNP analysis indicating a greater divergence between swamp- and river-type buffalo than between indicine and taurine cattle. We also demonstrated the impact of reference genome choice on genetic variant identification. The initial run of the 1000BGP identified numerous SNPs, including **polymorphic SNPs common to both buffalo types**, informing the design of a new genotyping SNP panel for the species. Our SNP analysis has also uncovered **non-synonymous mutations** in key genes such as *DGAT1* and *KISS1* that are associated with milk and reproductive traits, respectively. The 1000BGP is an on-going project with subsequent runs that will include more global samples for SNP discovery and serve as the foundation for other studies.

From: Dr Low Wai Yee, Senior Bioinformatician, Davies Research Centre
Faculty of Sciences / School of Animal and Veterinary Sciences
The University of Adelaide, Roseworthy Campus SA 5371, AUSTRALIA
Tel: +61 8313 0030 **Email:** wai.low@adelaide.edu.au **www.adelaide.edu.au**
CRICOS provider number 00123M

The manuscript and associated data have not been published elsewhere, nor are they under consideration by another journal. All authors have read and approved the submitted manuscript. Please address all correspondence to me at wai.low@adelaide.edu.au.

Yours faithfully,

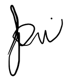

Low Wai Yee
University of Adelaide
Adelaide, Australia

From: Dr Low Wai Yee, Senior Bioinformatician, Davies Research Centre
Faculty of Sciences / School of Animal and Veterinary Sciences
The University of Adelaide, Roseworthy Campus SA 5371, AUSTRALIA
Tel: +61 8313 0030 **Email:** wai.low@adelaide.edu.au **www.adelaide.edu.au**
CRICOS provider number 00123M
