# Supplementary material for: Disentangling river and swamp buffalo genetic diversity: initial insights from the 1000 Buffalo Genomes Project
Source: Gigascience. 2024 Sep 9;13:giae053. doi: 10.1093/gigascience/giae053 (PMC11382405; doi:10.1093/gigascience/giae053)
Supplement: giae053_Supplemental_Files [file giae053_supplemental_files.zip › supplementary_figures.docx]

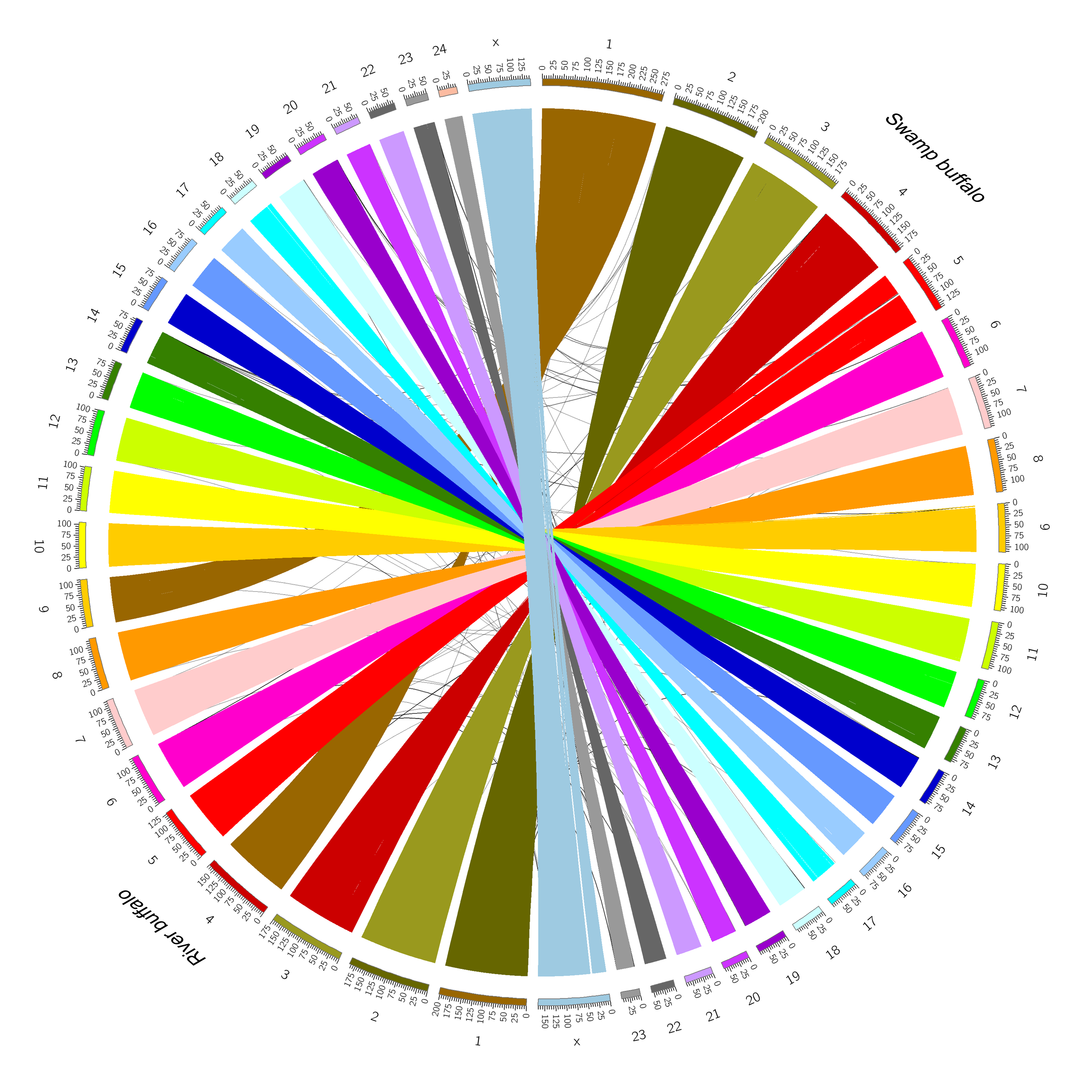
**Figure 1_Supplementary Material**. Circos plot of swamp buffalo chromosome mapped to river buffalo. Chromosome 1 of the swamp buffalo showed clear homology to Chromosomes 4 and 9 of the river buffalo.


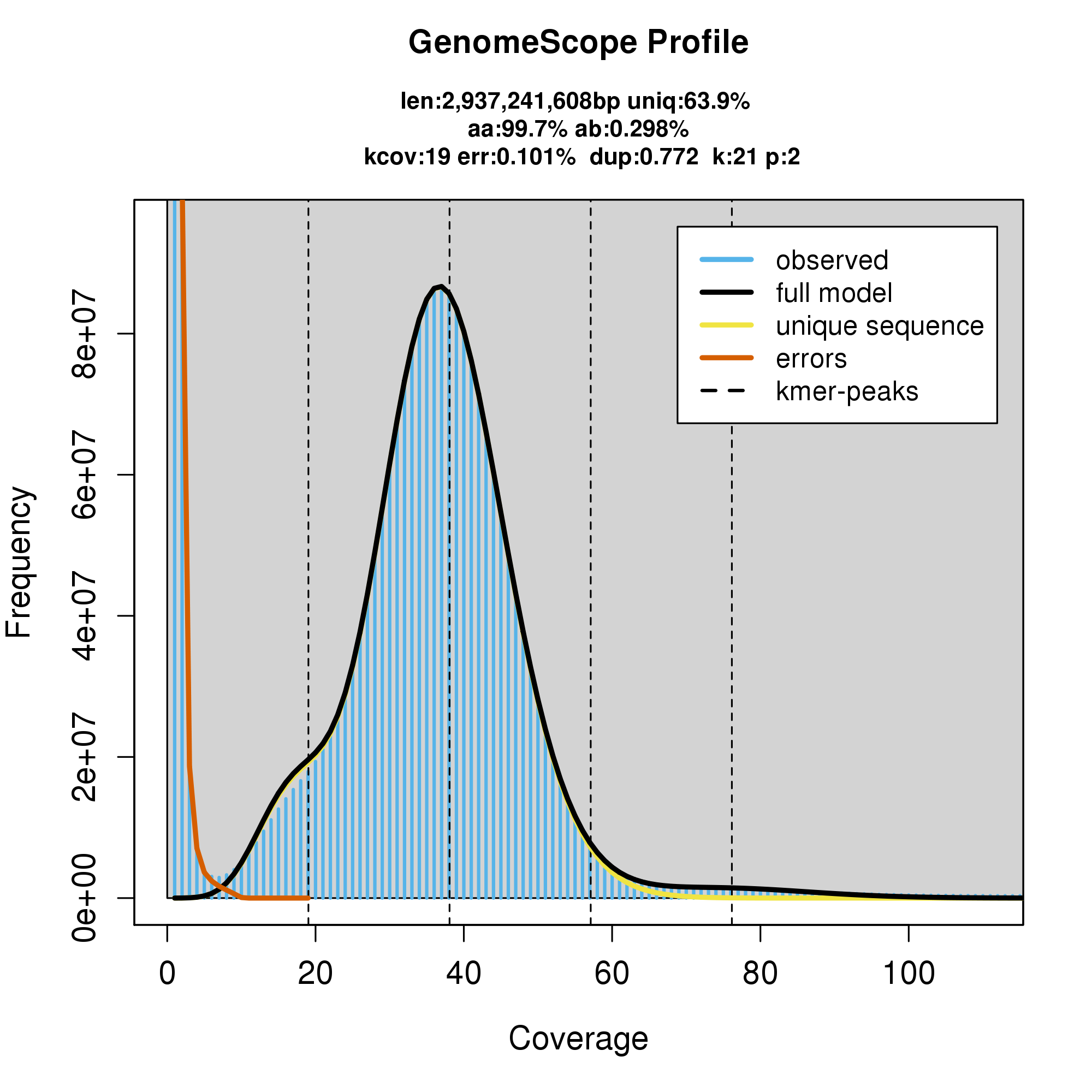
**Figure 2_Supplementary Material**. Genomescope2 profile showing k-mer spectra of the short-reads and inferring total genome length (len), percentage of the genome that are non-repetitive or unique (uniq), percentage of homozygosity (aa) and heterozygosity (ab), mean k-mer coverage for heterozygous bases (kcov), error rate of the reads (err), average rate of duplicate reads (dup), k-mer size used (k) and number of set of chromosomes (p).


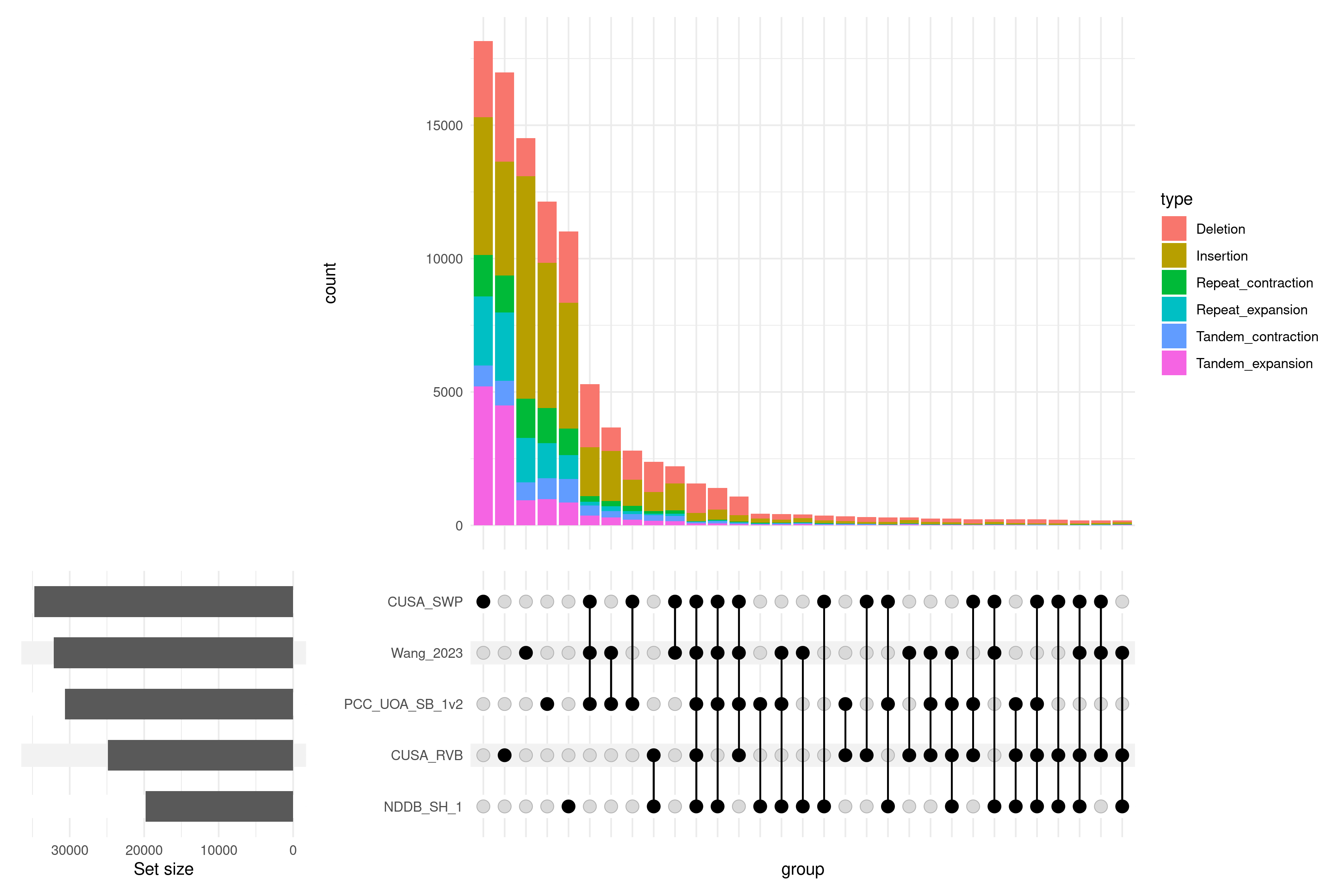
**Figure 3_Supplementary Material.** An upset plot of the number of different types of structural variants (SV) identified when aligned to UOA_WB_1 (river-type) which shows shared and unique SVs between various water buffalo assemblies.


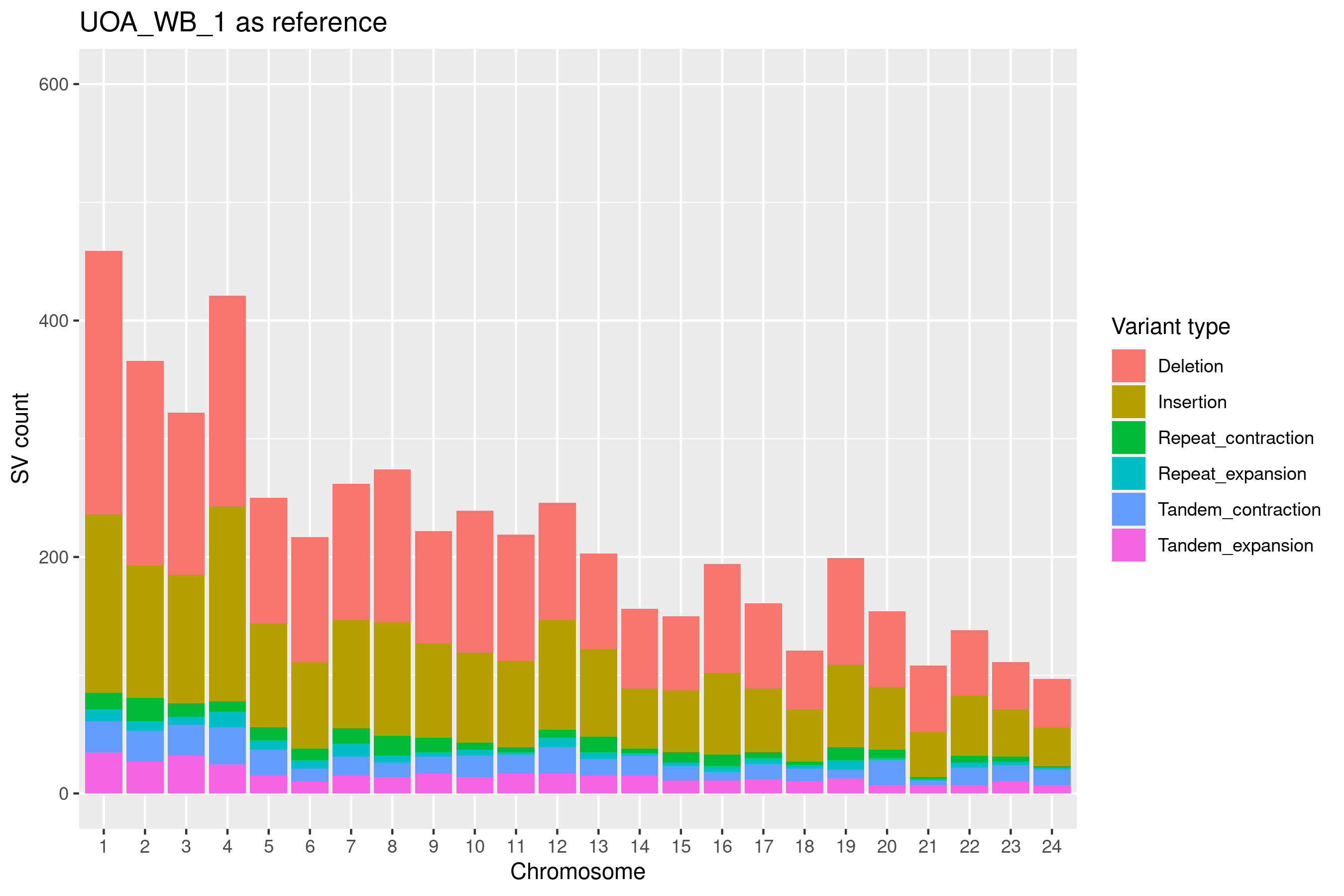
**Figure 4_Supplementary Material.** Bar graph of the number of the number of different types of structural variants (SV) shared between swamp buffalo assemblies (PCC_UOA_SB_1v2, Wang_2023 and CUSA_SWP) when aligned to river buffalo assembly (UOA_WB_1).


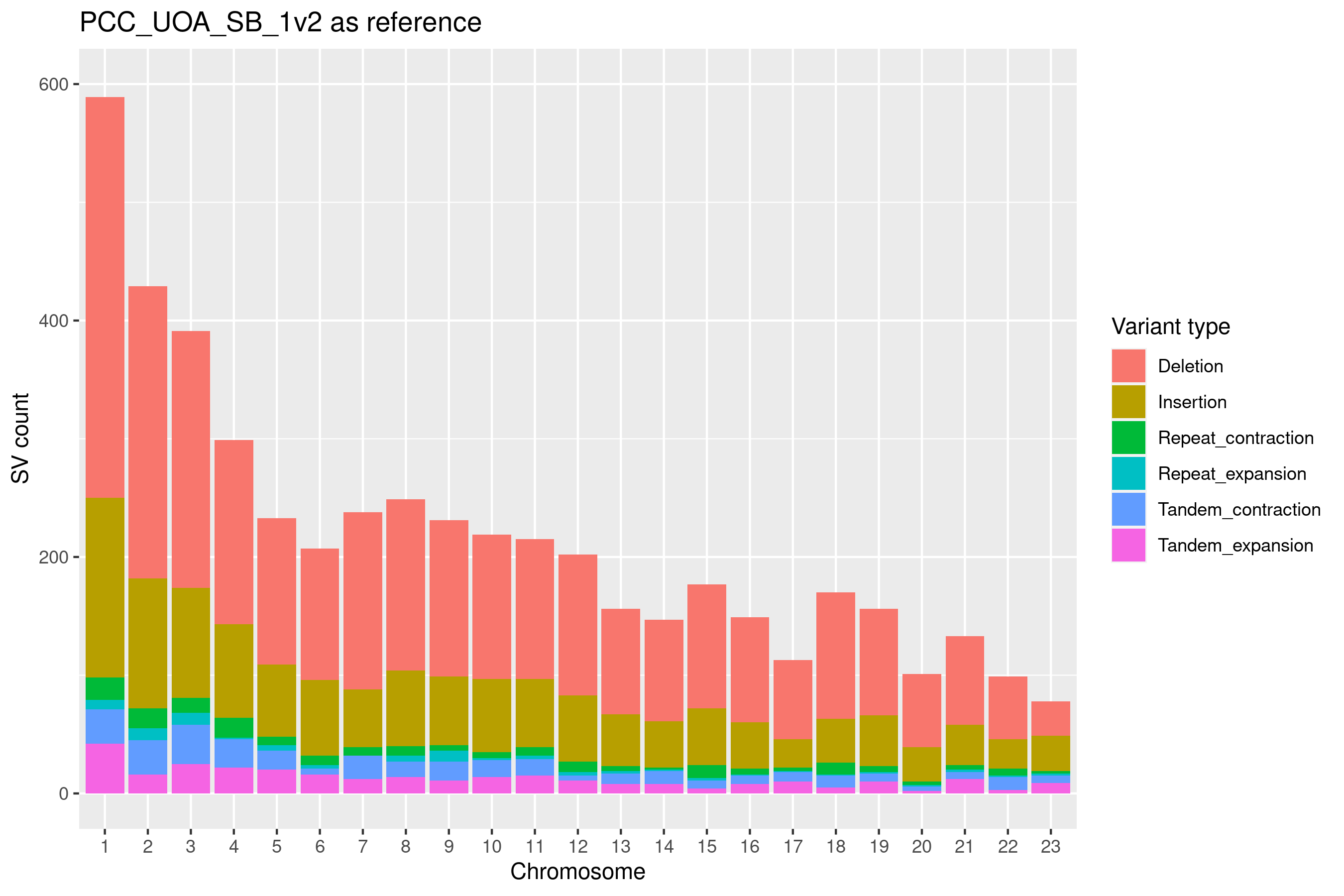
**Figure 5_Supplementary Material.** Bar graph of the number of the number of different types of structural variants (SV) shared between river buffalo assemblies (UOA_WB_1, NDDB_SH_1, CUSA_RVB) when aligned to swamp buffalo assembly (PCC_UOA_SB_1v2).


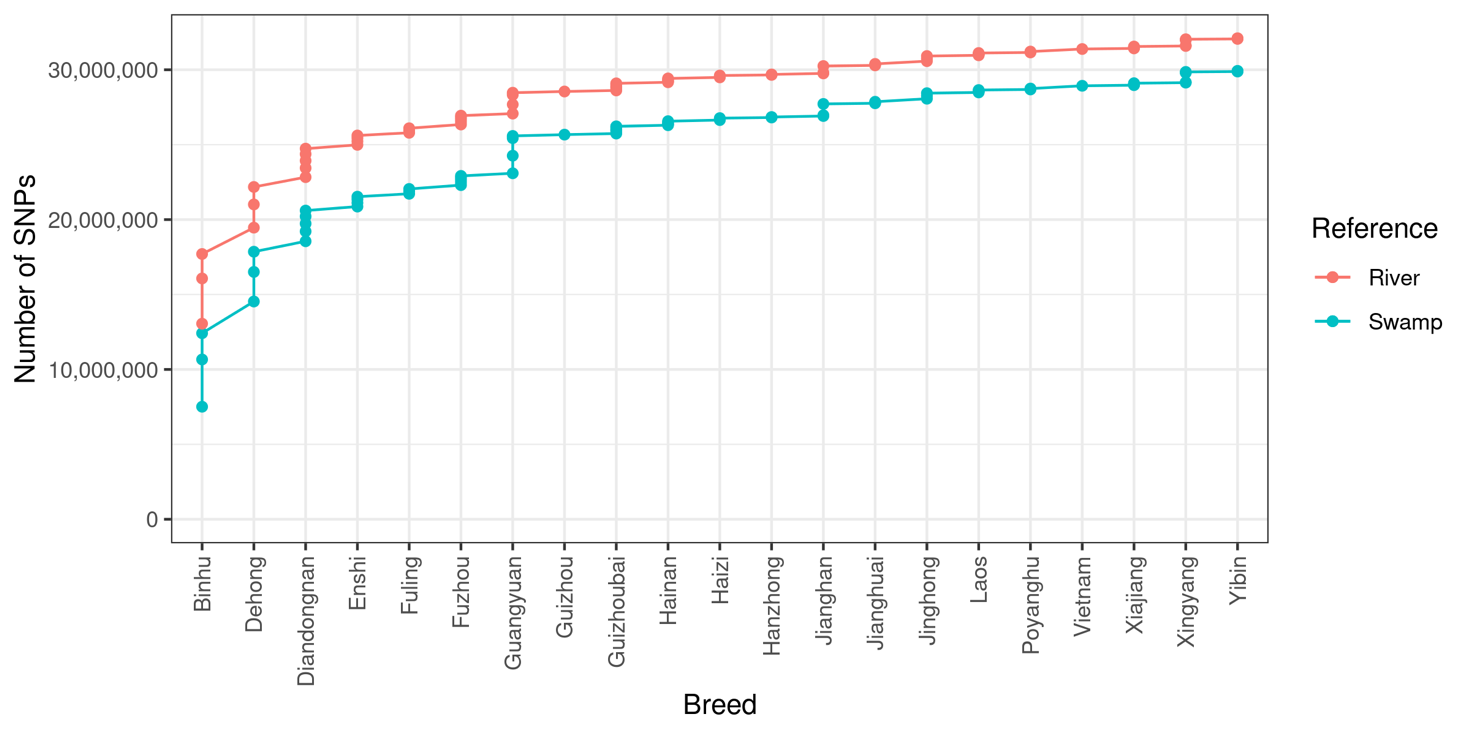


**Figure 6_Supplementary Material.** A line plot showing the cumulative number of SNPs of swamp-type buffalo samples per breeds when aligned to swamp or river buffalo reference genomes.


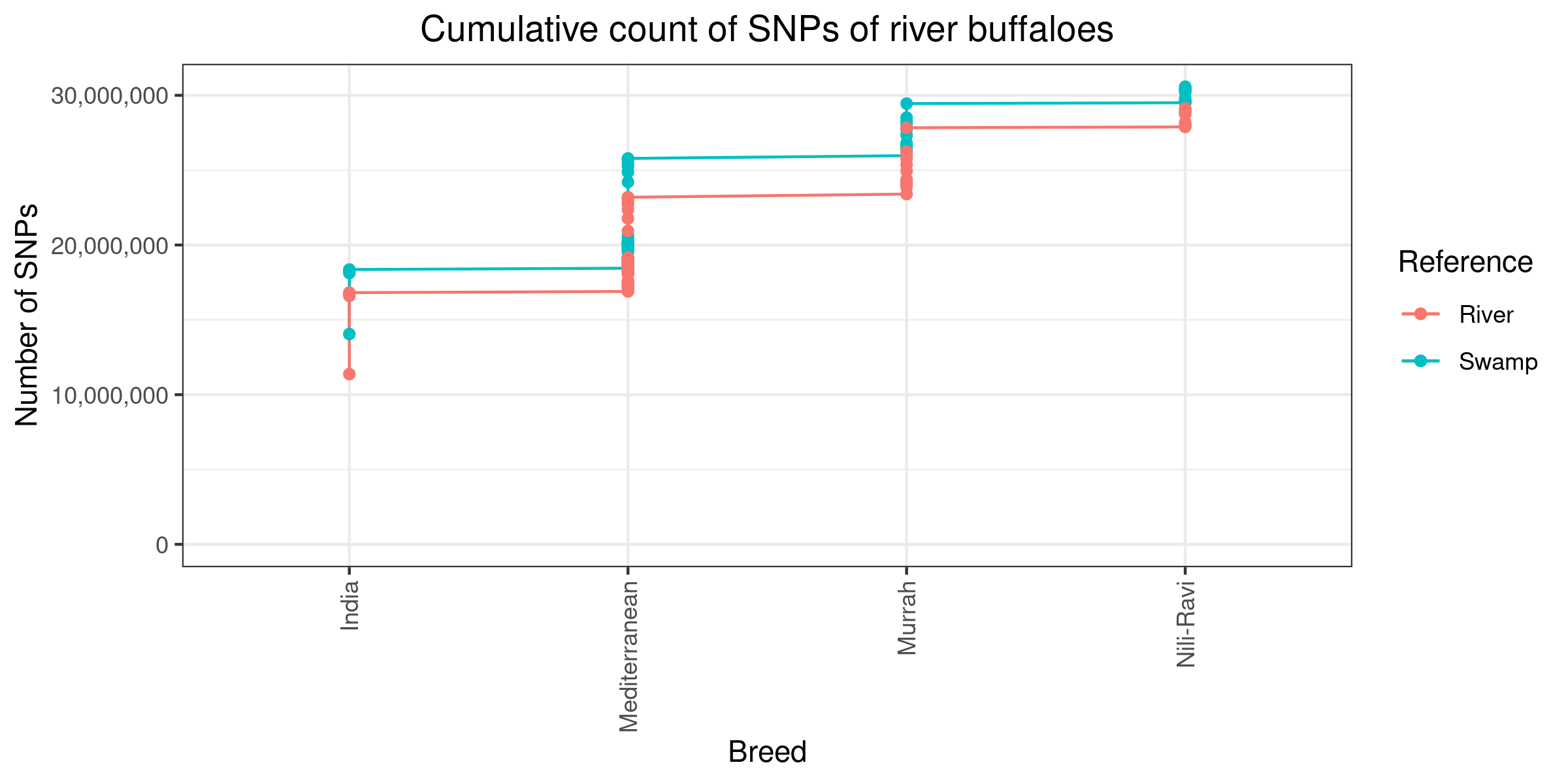


**Figure 7_Supplementary Material.** A line plot showing the cumulative number of SNPs of river type buffalo samples per breeds when aligned to swamp or river buffalo reference genomes.


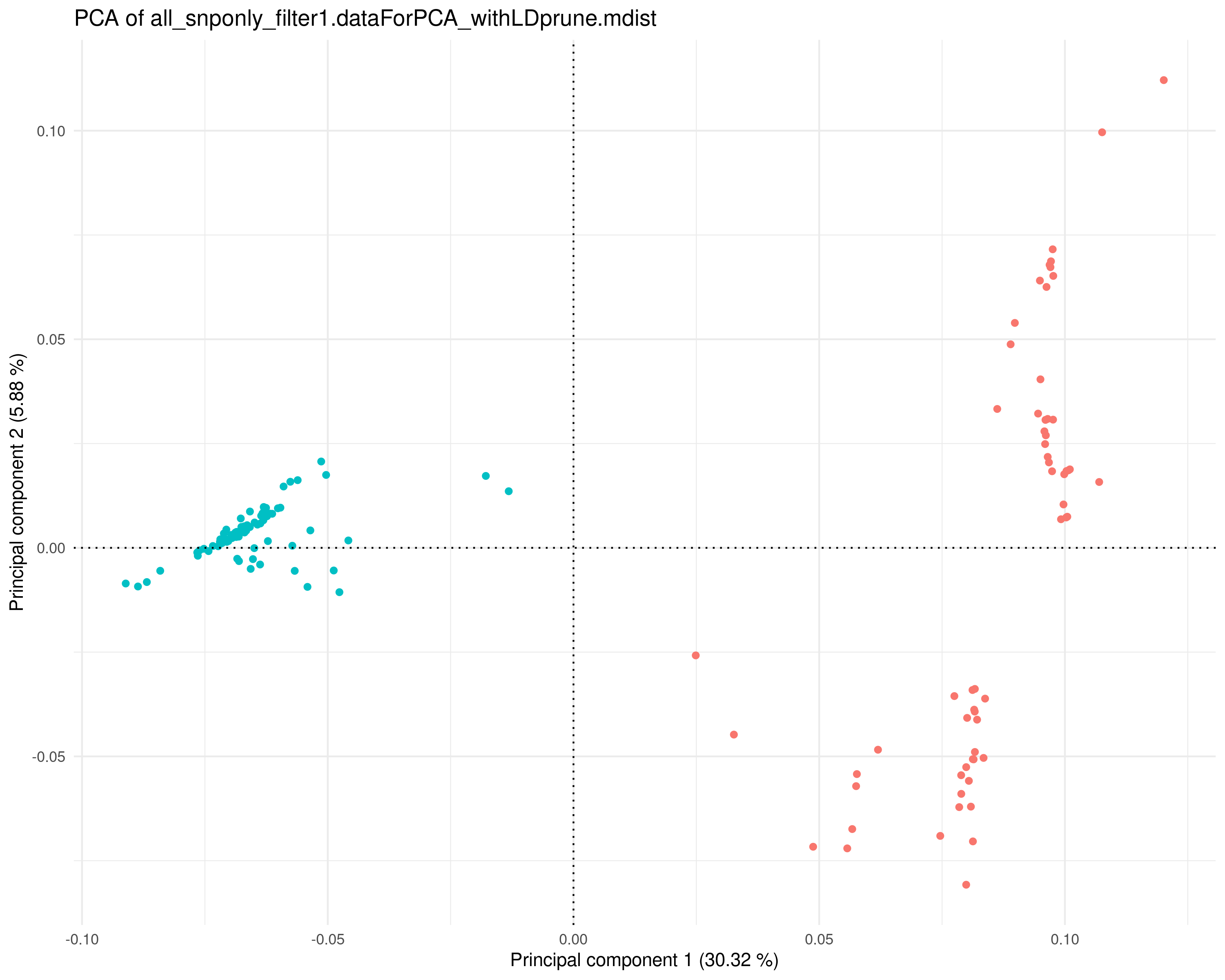
**Figure 8_Supplementary Material.** Principal component analysis (PCA) plot using the river buffalo reference genome (UOA_WB_1) shows clear clustering of the swamp and river buffaloes.
