# Supplementary material for: Disentangling river and swamp buffalo genetic diversity: initial insights from the 1000 Buffalo Genomes Project
Source: Gigascience. 2024 Sep 9;13:giae053. doi: 10.1093/gigascience/giae053 (PMC11382405; doi:10.1093/gigascience/giae053)
Supplement: giae053_Response_to_Reviewer_Comments_Original_Submission [file giae053_response_to_reviewer_comments_original_submission.pdf]

Author's Response To Reviewer Comments

Their reports, together with any other comments, are below.

The reviewers have a number of constructive (and overall relatively minor) comments, and I'm sure addressing these comments will make the paper stronger. I'd like to highlight comment #9 of reviewer 2 - submitting all data to relevant community-standard repositories is in line with GigaScience's policy. Please include respective accession numbers in the "data availability" section.

With respect to supporting data, please also note that we do not endorse the use of the phrase "data available on request" - all results and other supporting data or code needed to reproduce the analyses should be provided alongside the publication. We provide our database GigaDB for this purpose, and prior to publication our curators will work with you to make this data available.

Some other minor editorial points:
- Please cite data sources such as the figshare repo mentioned in the "availability" section via a numbered item in the bibliography, including the DOI.

Thank you for your pointing it out. We have included the reference for figshare repo mentioned.

- It's fine to include the "1000 buffaloes consortium" as a co-author, but please include at the end of the article a separate table that lists each individual consortium member's name and affiliation. The individuals in this list must all meet the ICMJE authorship guidelines.

We have added at the end of the article the list of 1000 buffalo genomes project consortium member and their affiliations. Thank you for your suggestion.

- Feel free to include a photo of the sequenced species as Fig.1 of the paper (if you do include this, you must have permission to publish the picture under creative commons (cc-by) terms).

Good suggestion! We have included the photo of the sequenced species as Figure 1 with permission under cc-by terms.

We have also made more rephrasing in the paper for clarity.

Reviewer 1

Pineda et al. present a new Swamp buffalo assembly, compare it to other existing water buffalo assemblies and call variants from 140 publicly available WGS samples against it and compare results to when aligning to a river buffalo.

It is great to see a new good quality water buffalo assembly. As the authors highlight, despite the global importance of the species, the availability of good quality assemblies is comparatively lacking. In particular good quality swamp buffalo assemblies. The authors make a good case for the quality of the assembly and it is good to see the authors have made a substantial effort to make the data and analyses available. I am less certain what to take from the variant calling section. I make some comments about this below but I think it would be good if made clearer the motivation and conclusions from this analysis. Just saying you get a different number of variant calls depending on the assembly used, swamp and river buffalo cluster separately etc is probably not completely surprising.

My comments in no particular order:

Why did the authors only take the sire haplotype from Ananthasayanam et al., 2020 and not the dam haplotype? They are of similar contiguity.

We chose representative breeds from available genome assemblies. Since both haplotype-resolved assemblies in Ananthasayanam et al., 2020 represent the Indian Murrah breed, we only need one of them. We chose the sire haplotype that has higher contiguity than the dam haplotype.

To say water buffalo are mainly found in Asia and Africa may make their presence in Africa sound bigger than it is, as in Africa they are only really found in Egypt and this makes up a very small proportion of the global population. So maybe reword?

We thank the reviewer for the suggestion. We have removed 'Africa' in the sentence because 97% of the water buffalo populations are found in Asia. This revision is at line 38.

Be good to mention Illumina sequencing amounts/coverages in methods, as do for the pac bio data.

Good suggestion. The coverage of Illumina short-reads was ~56x coverage, which we added at line 151. The coverage of raw PacBio HiFi was 29x as mentioned in line 125.

Can you give more details about how SNPs were defined from uniquely aligned sequences i.e. including how these regions were defined. I guess throughout this and similar sections also strictly speaking should be referring to SNVs not SNPs.

The reviewer is right to point out that SNV is more appropriate than SNP in this context. We have used the word 'SNV' as suggested at line 218. What we mean by uniquely aligned sequences are SNVs that do not fall in repeat region. These SNVs were found by using nucmer with `show-snps -Clr`. We removed the use of the term 'uniquely aligned sequences'. The revised sentence at line 223 is as follows: "SNVs were identified using the nucmer's 'show-snps –Clr' parameter to exclude SNVs within repeats."

Line 241. Describe how these 140 samples were selected. Given are not all of the high coverage buffalo samples that are publicly available.

The dataset is a publicly available dataset gathered by Yang Zhou of Huazhong Agricultural University, who is a member of the 1000 BGP. He analyzed the copy number variants and described this dataset in https://onlinelibrary.wiley.com/doi/full/10.1111/age.13288. Since this dataset has approximately half river- and half swamp-type buffalo, the 1000 BGP decided to start phase 1 with it. This dataset was almost all publicly available WGS data at the cut-off date for the phase 1 run. We have added a line describing how the samples were selected at line 230 with the following: "The chosen samples were based on submissions by members of the 1000 BGP and contained almost all publicly available WGS data on October 12, 2024".

Line 268. "covered more than 140 studies on water buffalo" should be precise. Ideally providing a list of studies as a supplementary table.

We have added Supplementary Table 5 to list the studies.

Line 296. Provide reference or ID of next best X chromosome.

We have added assembly name of the next best X-chromosome, which is UOA_WB_1 at line 288.

Line 323. Do the authors report where the candidate centromeres are? i.e. coordinates. I couldn't find this but may have missed it.

Yes, we provided the candidate centromeres in Supplementary Table 9, column "Position", sub-columns "start" and "end". However, our assembly has not fully resolved any centromere, which we have indicated at line 450 as follows: "There were no complete centromeres in any of our chromosomes, which was because the HiFi reads alone could not completely span the repeats in centromeres."

As chromosome 1 is a result of a relatively recent chromosomal fusion, how does the distribution of sat.673 relate to the fusion event?

Good question and we also wanted to analyse this in detail. However, as there were gaps in the putative centromeric region of chromosome 1, we do not have sufficient information to study the distribution of sat.673 at the fusion junction. We need a telomere-to-telomere assembly of chromosome 1 to answer this question.

Why do the authors think they don't get more indels when aligning the river WGS data to the swamp assembly than when aligning to the river assembly (Figure 4A). Wouldn't you expect to see more differences when aligning to the assembly from the other lineage? Is this indicative of a problem with the ability to call indels accurately? (Also assume is the 95% confidence interval of the mean, but good to clarify this)

We have also been puzzled by why we did not get a lot more indels when aligning river WGS to swamp reference. However, there are still more indels found when the river samples were aligned with the swamp buffalo reference. We double checked our scripts and found no errors. In fact, our pipeline is similar to the one used in our previous work on Brahman and Angus cattle that showed more SNPs and indels when aligning WGS data to the inappropriate reference (https://www.nature.com/articles/s41467-020-15848-y/tables/2). We indeed think one factor could be the inaccuracy in indel calling using short reads and GATK. It is known that indel calling is variable and have low concordance in different pipelines (see https://genomemedicine.biomedcentral.com/articles/10.1186/gm432) and efforts have been made to increase its accuracy (see https://genomemedicine.biomedcentral.com/articles/10.1186/s13073-014-0089-z). We reported the statistics as is for this work and did not use the indels for subsequent analysis. We have clarified the error bars of Figure 5A in its legend showing the standard deviation.

Figure 4B. Set y axis minimum to 0. Is this figure that useful? Surely depends on the order in which the samples are plotted. Beyond the impact of the first sample that clearly has less variants when aligned to swamp not sure how much can draw from this plot. The gap actually narrows even though say are all swamp samples.

Yes, our goal was to demonstrate that as more samples were added to the analysis, the number of new SNPs discovered decreased, showing a diminishing rate of novel SNP identification. We wanted to illustrate that with each additional sample, the incremental gain in detecting previously unseen SNPs became progressively smaller. This suggests that the majority of common SNPs were captured in the initial samples, and adding more samples yielded fewer and fewer unique SNPs, ultimately reaching a point of diminishing returns in terms of SNP discovery. However, after considering your point, we have moved this figure 4B to Supplementary Figure 6 instead.

Not sure I fully follow this but is Figure 4D suggesting there are no river specific variants in the larger set? In that the darker peach circle is fully within the lighter peach circle?

Yes, there are no water buffalo specific SNPs found in the lighter peach circle. Yes, the darker peach circle is fully within the lighter peach circle. Please refer to Table 3 if you need more information.

Obviously the blips in Figure 4E are odd. Is this just an artefact of the bins chosen and the fact only certain allele frequencies are possible. If so is there a way to change this e.g. by changing bin sizes.

Yes, it is an effect of the number of SNPs and binning size. However, changing the bin sizes gives the same effect. We have changed Figure 5D to a density plot to avoid the effect of binning size in the plot.

"There were ~5 million SNPs polymorphic SNPs in river that were fixed in swamp buffaloes" I couldn't find how this analysis was done. Is this when aligning to the swamp buffalo genome? If so obviously if aligned to the river buffalo genome a lot of the fixed sites will no longer be fixed. So this analysis, and its interpretation, depends on what genome are aligning to. Also should remove first "SNPs" from this sentence (and polymorphic SNPs is a tautology as by definition SNPs are polymorphic).

We have removed instances where we said ‘polymorphic SNPs’. Thanks for the suggestion. Regarding the ~5 million SNPs, we have now included it in the Methods at line 250 as follows: “We identified ~1.5 million

variants that are highly polymorphic in swamp (MAF > 0.2) but were fixed in river buffaloes (MAF < 0.01) and ~5 million SNPs in river that were fixed in swamp buffaloes."

What was the Ti/Tv ratios of the variant calls and does it suggest good final call sets.

The Ti/Tv ratio of the variant calls with swamp and river buffalo reference genome is 2.13 and 2.12, respectively. This suggests we have good final call sets since the optimal ratio is around ~2. We have added this information at line 385.

It's a bit odd that the authors effectively present results in the discussion e.g. presenting DGAT1 variant. Should this not be in results? Especially as is mentioned in the abstract. Would also be good to investigate this further i.e. is there actually evidence of a selective sweep at this location as hypothesised.

Our apology for including this in the wrong section. We have moved the DGAT1 variant result to the Result section. While we agree a selective sweep analysis will be nice, this is not the scope of this paper and others in the 1000BGP have planned for such analysis in the future.

I would remove the word "impressive" from line 564.

We have removed "impressive".

I am surprised that the authors seem to be suggesting that there is a correlation in Figure 2B (though no P value is actually reported). The two axes appear pretty uncorrelated. Would a Spearman's rank correlation be more appropriate here.

Yes, we agree that Spearman's rank would be a more appropriate analysis. Although there is a weak positive correlation, the result is not statistically significant as the p-value is 0.16. We initially conducted this analysis to see if the divergence of swamp-river is high enough that it is more like species level divergence rather than subspecies level divergence. However, based on your observation and the insignificant p-value, we now realized that our analysis cannot be used to meaningfully justify divergence level of the chosen species/subspecies pairs. We have decided to remove this figure 2B and associated results and discussion.

"There were 5,289 SVs that were shared by the three swamp buffalo assemblies when compared to the river buffalo reference (UOA_WB_1)." I cant see this bar in the plot? No bar seems to have this height and not the set they are referring to?

It is in Supplementary Figure 4 with group CUSA_SWP, Wang-2023 and PCC_UOA_SB_1v2 combined. We have revised the sentence at line 377 as follows: "There were 5,289 SVs that were shared by the three swamp buffalo assemblies when compared to the river buffalo reference (UOA_WB_1) (Figure 4_Supplementary Material)."

There seemed to be quite a few spelling/grammar mistakes. I list a few here but there were several more so suggest the authors go over it again.
Line 64 should be "resulting in a"

Our apology for the grammatical errors and typos. We changed "to" to "in" at line 62 as suggested.

Line 170. Should be were not where Line 225. Assume should be The not he Line 293 should be has not have

The corrections were made at line 167 and 285. We have removed the section containing Line 225.

Reviewer 2
This paper describes the generation of a new genome assembly (PCC_UOA_SB_1v2) for the swamp buffalo. A variety of metrics are used to show that the new assembly represents an improvement over

existing water buffalo assemblies. 140 additional buffalo genomes (short read datasets) are analyzed using the new assembly and that of a river buffalo (assembly UOA_WB_1), for the purpose of identifying sequence variants (SNPs, indels, and SVs).
The new assembly is used to estimate divergence time between swamp and river buffalo, and between buffaloes and other mammals.

Variants that are fixed or polymorphic within the two subspecies are identified. In the discussion, variants in DGAT1, KISS1, and KISS1R are highlighted as being of interest due to their known association with milk and reproductive traits.

General comments ---------------------

The paper describes a thorough and well-executed analysis that will be of interest to researchers working on buffalo genomics.

The rationale for carrying out the divergence estimate aspects isn't clear given the modest improvements in the assembly and the nature of the regions likely used in these analyses (i.e. regions are likely to have been well-characterized in the previous assembly). The authors should provide a more detailed justification for these analyses.

A valuable collection of sequence data, results files and scripts are being made available with the manuscript. However, it isn't clear whether or how certain data files will be made available to the public (see comment below).

We thank the reviewer for the comments. Regarding dataset and results, we will make them publicly available (see below for details). Regarding divergence estimates, we initially conducted this analysis to see if the divergence of swamp-river is high enough that it is more like species level divergence rather than subspecies level divergence. We re-analysed the divergence estimate result and found that the relationship between nucleotide substitution per site and divergence time (Mya) was not statistically significant. As pointed out by the reviewer, similar divergence estimates have been made available so the analysis is redundant. We have decided to remove Figure 2B and associated results and discussion.

Specific comments ---------------------

1. Minor grammatical errors are present throughout the manuscript. Inconsistent tense is used. The paper would benefit from thorough proofreading and editing.

We revised the manuscript to correct the tenses used.

2. The statement in the abstract "in genes such as DGAT1 and KISS1 that were associated with milk and reproductive traits, respectively" may incorrectly give the impression that these associations were established in the paper. Moreover the identification of these SNPs in this study doesn't warrant inclusion in the abstract in my opinion. Also the phrase "in genes such as" isn't meaningful.

Following your suggestion, we have removed the statement from the abstract.

3. The statement "38 researchers who work on water buffalo from 15 countries" should be revised for clarity unless the buffalo are from 15 countries.

We revised the statement to "38 researchers who have previous works on water buffalo from 15 countries at line 81.

4. I suggest stating the name of new assembly name in the abstract, and removing the name of the old assembly.

We have added the assembly name "PCC_UOA_SB_1" for clarity and removed UOA_WB_1 in the abstract.

5. Line 225, missing "T" in "he species/subspecies"

The results on species/subspecies divergence have all been removed.

6. Line 284, rephrase "137 contigs with assembly size of 2.90 Gb were retained and contig N50 of 91.17 Mb."

We thank the reviewer for the suggestion and we have rephased it to “137 contigs with assembly size of 2.90 Gb and a contig N50 of 91.17 Mb were retained” at line 275.

7. Line 417 Revise "which have minor impact" to indicate these are predictions in "Moreover ~99% were SNPs classified as modifiers by snpEff, which have minor impact and are often found in non-coding regions."

We rephrased it to “Moreover, ~99% were SNPs classified as modifiers by snpEff, that were predicted to have minor impact as they are often found in non-coding regions” at line 397.

8. In the "Repeats resolution" section of results, revise the first sentence to remove the parentheses around the statement after the "e.g.".

We rephrased the sentence to “for instance, total percentage of repeats” to avoid the parenthesis at line 298.

9. Will the sequence data, genotypes, or variant lists be made available for the 140 samples analyzed in this study? Typically the sequence data would be deposited in the SRA/ENA and the variants in the EVA.

Yes, all results of the first run of 1000 BGP will be publicly available. The genotypes etc have been uploaded to the GigaScience repository. The SRA accession number of the 140 samples can be found in Supplementary Table 5.

10. The folder of scripts and data files could be better organized through the use of subfolders.

We thank the reviewer for this suggestion. We have organized the scripts etc using sub-folders.
