# Supplementary material for: Disentangling river and swamp buffalo genetic diversity: initial insights from the 1000 Buffalo Genomes Project
Source: Gigascience. 2024 Sep 9;13:giae053. doi: 10.1093/gigascience/giae053 (PMC11382405; doi:10.1093/gigascience/giae053)
Supplement: giae053_Response_to_Reviewer_Comments_Revision_1 [file giae053_response_to_reviewer_comments_revision_1.pdf]

Author's Response To Reviewer Comments

Thank you very much for accepting our manuscript for publication. We also appreciate your pointers and have made the following revisions:

1. Added citations at REFERENCES section at number 91 and short description of the data at Line 677.
2. Moved the URL citations to the REFERENCE section, specifically at entries 20, 22, 36, 37, 45 and 48.
3. Included the DOI at entry 90 in the REFERENCES section.
4. Added the ORCID number of the authors at line 3-8.
5. Provided the NCBI taxon number for swamp and river buffalo at line 102, 169 and 171.
6. Restructured the abstract as suggested, now at line 22-38.

Thank you once again for your guidance and assistance!

Best regards,
Paulene S. Pineda
