# Supplementary material for: Disentangling river and swamp buffalo genetic diversity: initial insights from the 1000 Buffalo Genomes Project
Source: Gigascience. 2024 Sep 9;13:giae053. doi: 10.1093/gigascience/giae053 (PMC11382405; doi:10.1093/gigascience/giae053)
Supplement: giae053_Reviewer_1_Report_Original_Submission — James Prendergast -- 4/23/2024 Reviewed [file giae053_reviewer_1_report_original_submission.pdf]

Reviewer Report

Title: Disentangling river- and swamp-buffalo genetic diversity: Initial Insights from the 1000 Buffalo Genomes Project

Version: Original Submission **Date:** 4/23/2024

Reviewer name: James Prendergast

Reviewer Comments to Author:

Pineda et al. present a new Swamp buffalo assembly, compare it to other existing water buffalo assemblies and call variants from 140 publicly available WGS samples against it and compare results to when aligning to a river buffalo.

It is great to see a new good quality water buffalo assembly. As the authors highlight, despite the global importance of the species, the availability of good quality assemblies is comparatively lacking. In particular good quality swamp buffalo assemblies. The authors make a good case for the quality of the assembly and it is good to see the authors have made a substantial effort to make the data and analyses available. I am less certain what to take from the variant calling section. I make some comments about this below but I think it would be good if made clearer the motivation and conclusions from this analysis. Just saying you get a different number of variant calls depending on the assembly used, swamp and river buffalo cluster separately etc is probably not completely surprising.

My comments in no particular order:

Why did the authors only take the sire haplotype from Ananthasayanam et al., 2020 and not the dam haplotype? They are of similar contiguity.

To say water buffalo are mainly found in Asia and Africa may make their presence in Africa sound bigger than it is, as in Africa they are only really found in Egypt and this makes up a very small proportion of the global population. So maybe reword?

Be good to mention Illumina sequencing amounts/coverages in methods, as do for the pac bio data.

Can you give more details about how SNPs were defined from uniquely aligned sequences i.e. including how these regions were defined. I guess throughout this and similar sections also strictly speaking should be referring to SNVs not SNPs.

Line 241. Describe how these 140 samples were selected. Given are not all of the high coverage buffalo samples that are publicly available.

Line 268. "covered more than 140 studies on water buffalo" should be precise. Ideally providing a list of studies as a supplementary table.

Line 296. Provide reference or ID of next best X chromosome.

Line 323. Do the authors report where the candidate centromeres are? i.e. coordinates. I couldn't find this but may have missed it.

As chromosome 1 is a result of a relatively recent chromosomal fusion, how does the distribution of sat.673 relate to the fusion event?

Why do the authors think they don't get more indels when aligning the river WGS data to the swamp assembly than when aligning to the river assembly (Figure 4A). Wouldn't you expect to see more

differences when aligning to the assembly from the other lineage? Is this indicative of a problem with the ability to call indels accurately? (Also assume is the 95% confidence interval of the mean, but good to clarify this)
Figure 4B. Set y axis minimum to 0. Is this figure that useful? Surely depends on the order in which the samples are plotted. Beyond the impact of the first sample that clearly has less variants when aligned to swamp not sure how much can draw from this plot. The gap actually narrows even though say are all swamp samples.
Not sure I fully follow this but is Figure 4D suggesting there are no river specific variants in the larger set? In that the darker peach circle is fully within the lighter peach circle?
Obviously the blips in Figure 4E are odd. Is this just an artefact of the bins chosen and the fact only certain allele frequencies are possible. If so is there a way to change this e.g. by changing bin sizes.
"There were ~5 million SNPs polymorphic SNPs in river that were fixed in swamp buffaloes" I couldn't find how this analysis was done. Is this when aligning to the swamp buffalo genome? If so obviously if aligned to the river buffalo genome a lot of the fixed sites will no longer be fixed. So this analysis, and its interpretation, depends on what genome are aligning to. Also should remove first "SNPs" from this sentence (and polymorphic SNPs is a tautology as by definition SNPs are polymorphic).
What was the Ti/Tv ratios of the variant calls and does it suggest good final call sets.
It's a bit odd that the authors effectively present results in the discussion e.g. presenting DGAT1 variant. Should this not be in results? Especially as is mentioned in the abstract. Would also be good to investigate this further i.e. is there actually evidence of a selective sweep at this location as hypothesised.
I would remove the word "impressive" from line 564.
I am surprised that the authors seem to be suggesting that there is a correlation in Figure 2B (though no P value is actually reported). The two axes appear pretty uncorrelated. Would a Spearman's rank correlation be more appropriate here.
"There were 5,289 SVs that were shared by the three swamp buffalo assemblies when compared to the river buffalo reference (UOA_WB_1)." I cant see this bar in the plot? No bar seems to have this height and not the set they are referring to?
There seemed to be quite a few spelling/grammar mistakes. I list a few here but there were several more so suggest the authors go over it again.
Line 64 should be "resulting in a"
Line 170. Should be were not where
Line 225. Assume should be The not he
Line 293 should be has not have

Methods

Are the methods appropriate to the aims of the study, are they well described, and are necessary controls included? Choose an item.

Conclusions

Are the conclusions adequately supported by the data shown? Choose an item.

Reporting Standards

Does the manuscript adhere to the journal's guidelines on [minimum standards of reporting?] Choose an item.

Choose an item.

Statistics

Are you able to assess all statistics in the manuscript, including the appropriateness of statistical tests used? Choose an item.

Quality of Written English

Please indicate the quality of language in the manuscript: Choose an item.

Declaration of Competing Interests

Please complete a declaration of competing interests, considering the following questions:

- Have you in the past five years received reimbursements, fees, funding, or salary from an organisation that may in any way gain or lose financially from the publication of this manuscript, either now or in the future?
- Do you hold any stocks or shares in an organisation that may in any way gain or lose financially from the publication of this manuscript, either now or in the future?
- Do you hold or are you currently applying for any patents relating to the content of the manuscript?
- Have you received reimbursements, fees, funding, or salary from an organization that holds or has applied for patents relating to the content of the manuscript?
- Do you have any other financial competing interests?
- Do you have any non-financial competing interests in relation to this paper?

If you can answer no to all of the above, write 'I declare that I have no competing interests' below. If your reply is yes to any, please give details below.

I declare that I have no competing interests

I agree to the open peer review policy of the journal. I understand that my name will be included on my report to the authors and, if the manuscript is accepted for publication, my named report including any attachments I upload will be posted on the website along with the authors' responses. I agree for my report to be made available under an Open Access Creative Commons CC-BY license (http://creativecommons.org/licenses/by/4.0/). I understand that any comments which I do not wish to

be included in my named report can be included as confidential comments to the editors, which will not be published.

Choose an item.

To further support our reviewers, we have joined with Publons, where you can gain additional credit to further highlight your hard work (see: https://publons.com/journal/530/gigascience). On publication of this paper, your review will be automatically added to Publons, you can then choose whether or not to claim your Publons credit. I understand this statement.

Yes Choose an item.
