# Supplementary material for: Disentangling river and swamp buffalo genetic diversity: initial insights from the 1000 Buffalo Genomes Project
Source: Gigascience. 2024 Sep 9;13:giae053. doi: 10.1093/gigascience/giae053 (PMC11382405; doi:10.1093/gigascience/giae053)
Supplement: giae053_Reviewer_2_Report_Original_Submission — Paul Stothard -- 4/25/2024 Reviewed [file giae053_reviewer_2_report_original_submission.pdf]

Reviewer Report

Title: Disentangling river- and swamp-buffalo genetic diversity: Initial Insights from the 1000 Buffalo Genomes Project

Version: Original Submission **Date:** 4/25/2024

Reviewer name: Paul Stothard

Reviewer Comments to Author:

This paper describes the generation of a new genome assembly (PCC_UOA_SB_1v2) for the swamp buffalo. A variety of metrics are used to show that the new assembly represents an improvement over existing water buffalo assemblies. 140 additional buffalo genomes (short read datasets) are analyzed using the new assembly and that of a river buffalo (assembly UOA_WB_1), for the purpose of identifying sequence variants (SNPs, indels, and SVs).
The new assembly is used to estimate divergence time between swamp and river buffalo, and between buffaloes and other mammals.
Variants that are fixed or polymorphic within the two subspecies are identified. In the discussion, variants in DGAT1, KISS1, and KISS1R are highlighted as being of interest due to their known association with milk and reproductive traits.
General comments

The paper describes a thorough and well-executed analysis that will be of interest to researchers working on buffalo genomics.
The rationale for carrying out the divergence estimate aspects isn't clear given the modest improvements in the assembly and the nature of the regions likely used in these analyses (i.e. regions are likely to have been well-characterized in the previous assembly). The authors should provide a more detailed justification for these analyses.
A valuable collection of sequence data, results files and scripts are being made available with the manuscript. However, it isn't clear whether or how certain data files will be made available to the public (see comment below).
Specific comments
----
1. Minor grammatical errors are present throughout the manuscript. Inconsistent tense is used. The paper would benefit from thorough proofreading and editing.
2. The statement in the abstract "in genes such as DGAT1 and KISS1 that were associated with milk and reproductive traits, respectively" may incorrectly give the impression that these associations were established in the paper. Moreover the identification of these SNPs in this study doesn't warrant inclusion in the abstract in my opinion. Also the phrase "in genes such as" isn't meaningful.
3. The statement "38 researchers who work on water buffalo from 15 countries" should be revised for clarity unless the buffalo are from 15 countries.
4. I suggest stating the name of new assembly name in the abstract, and removing the name of the old

assembly.
5. Line 225, missing "T" in "he species/subspecies"
6. Line 284, rephrase "137 contigs with assembly size of 2.90 Gb were retained and contig N50 of 91.17 Mb."
7. Line 417 Revise "which have minor impact" to indicate these are predictions in "Moreover ~99% were SNPs classified as modifiers by snpEff, which have minor impact and are often found in non-coding regions."
8. In the "Repeats resolution" section of results, revise the first sentence to remove the parentheses around the statement after the "e.g.".
9. Will the sequence data, genotypes, or variant lists be made available for the 140 samples analyzed in this study? Typically the sequence data would be deposited in the SRA/ENA and the variants in the EVA.
10. The folder of scripts and data files could be better organized through the use of subfolders.

Methods

Are the methods appropriate to the aims of the study, are they well described, and are necessary controls included? Choose an item.

Conclusions

Are the conclusions adequately supported by the data shown? Choose an item.

Reporting Standards

Does the manuscript adhere to the journal's guidelines on minimum standards of reporting? Choose an item.

Choose an item.

Statistics

Are you able to assess all statistics in the manuscript, including the appropriateness of statistical tests used? Choose an item.

Quality of Written English

Please indicate the quality of language in the manuscript: Choose an item.

Declaration of Competing Interests

Please complete a declaration of competing interests, considering the following questions:

- Have you in the past five years received reimbursements, fees, funding, or salary from an organisation that may in any way gain or lose financially from the publication of this manuscript, either now or in the future?

- Do you hold any stocks or shares in an organisation that may in any way gain or lose financially from the publication of this manuscript, either now or in the future?
- Do you hold or are you currently applying for any patents relating to the content of the manuscript?
- Have you received reimbursements, fees, funding, or salary from an organization that holds or has applied for patents relating to the content of the manuscript?
- Do you have any other financial competing interests?
- Do you have any non-financial competing interests in relation to this paper?

If you can answer no to all of the above, write 'I declare that I have no competing interests' below. If your reply is yes to any, please give details below.

I declare that I have no competing interests

I agree to the open peer review policy of the journal. I understand that my name will be included on my report to the authors and, if the manuscript is accepted for publication, my named report including any attachments I upload will be posted on the website along with the authors' responses. I agree for my report to be made available under an Open Access Creative Commons CC-BY license (http://creativecommons.org/licenses/by/4.0/). I understand that any comments which I do not wish to be included in my named report can be included as confidential comments to the editors, which will not be published.

Choose an item.

To further support our reviewers, we have joined with Publons, where you can gain additional credit to further highlight your hard work (see: https://publons.com/journal/530/gigascience). On publication of this paper, your review will be automatically added to Publons, you can then choose whether or not to claim your Publons credit. I understand this statement.

Yes Choose an item.
